# Magma and tephra characteristics for the 17–25 May 2016 Mt Etna eruption

**DOI:** 10.1016/j.dib.2018.11.093

**Published:** 2018-11-26

**Authors:** M.J. Edwards, L. Pioli

**Affiliations:** Department of Earth Sciences, University of Geneva, 13 Rue des Maraîchers, 1205 Genève, Switzerland

## Abstract

We provide the dataset associated with the research data article “Shallow factors controlling the explosivity of basaltic magmas: The 17–25 May 2016 eruption of Etna Volcano (Italy)” Edwards et al. This dataset contains major element data for groundmass glass, plagioclase, olivine and clinopyroxene phenocrysts, and melt inclusions within these phenocrysts, found within tephra and lava from this eruption. We also provide the grain size dataset from the fallout deposits.

**Specifications table**

**Table:** 

Subject area	*Geology*
More specific subject area	*Volcanology; Geochemistry*
Type of data	*Tables and figures*
How data were acquired	*JEOL JXA 8200 Superprobe. Sieving.*
Data format	*Raw*
Experimental factors	*Melt inclusions free from any crystallisation or alteration selected in carbon-coated thin sections.*
Experimental features	*Operating conditions for electron microprobe were: accelerating voltage of 10 kV; beam current of 10–15 nA, beam diameter of 3–10 μm; counting times of 8–20 s on peak positions and 4–10 s on background positions.*
Data source location	*Samples were analysed at the University of Geneva, Geneva, Switzerland. Sampling locations are listed in*Tables 1 and 2.
Data accessibility	*With this data article.*
Related research article	*Edwards, M.J., Pioli, L., Andronico, D., Scollo, S., Ferrari, F., Cristaldi, A (2018). Shallow factors controlling the explosivity of basaltic magmas: The 17–25 May 2016 eruption of Etna Volcano (Italy). Journal of Volcanology and Geothermal Research, 357:425–436. doi:* 10.1016/j.jvolgeores.2018.05.015

**Value of the data**

• The grain size data could be used for comparison with other eruptions to evaluate a relationship between eruption parameters and total grain size distributions.
• Detailed compositional data for phenocrysts, groundmass glass and melt inclusions could be used in future studies of the plumbing system of Mount Etna or any other petrology investigation on basalt chemistry.

## 1 Data

**Fig. 1:**
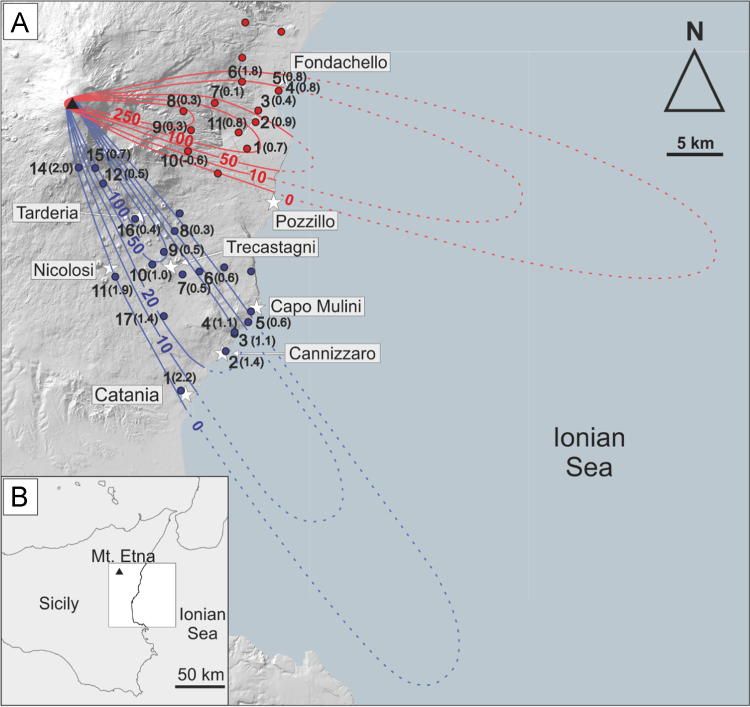
A) Sampling locations with Mdphi at each in parentheses. Isolines represent the mass load in g/m^2^ for the May 18 and 19 deposit in red, and the May 21 deposit in blue. B) Inset location relative to Sicily.

**Fig. 2:**
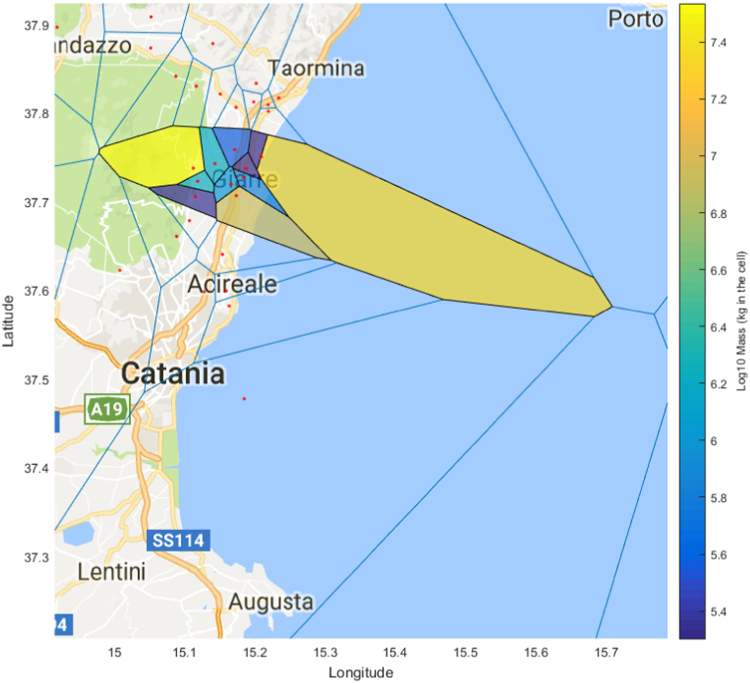
Voronoi polygons used for total grain size distribution of the 18 and 19 May 2016 deposit.

**Fig. 3:**
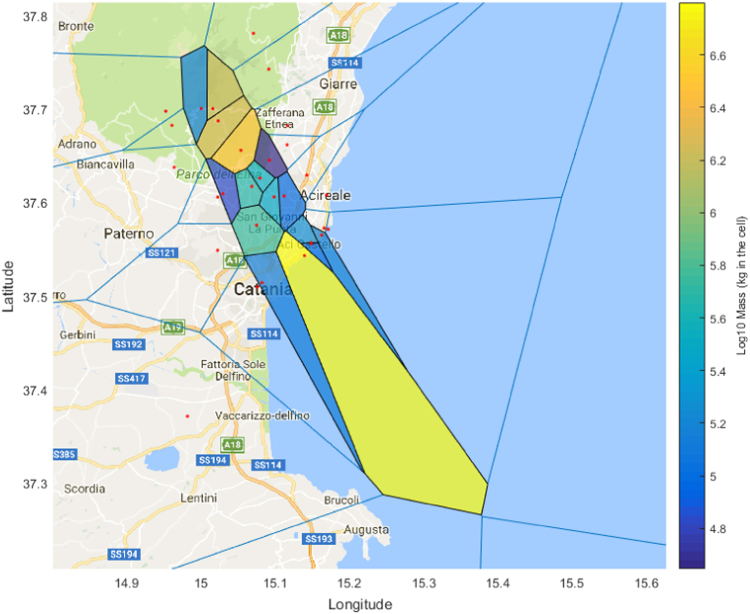
Voronoi polygons used for total grain size distribution of the 21 May 2016 deposit.

Tables 1 and 2 contain tephra grain size distribution data for each of the 11 and 16 locations sampled for the deposit of the 18 and 19 May 2016 eruption plume, and 21 May eruption plume respectively. Tables S1–S4 contain all of the raw elemental data for the plagioclase, clinopyroxene and olivine phenocrysts. Table S4 contains raw elemental data for the groundmass glasses and melt inclusions. Fig. 1 shows the Mdphi values measured at each sampling location; Figs. 2 and 3 show the distribution of the Voronoi polygons used for computation of the total grain size distribution of both deposits (as per [1]).

**Table 1:** 18 and 19 May 2016 deposit grain size data.

**Sample Number**	**GPS**		**Grain size (mm)**															
	**N**	**E**	**0.032**	**0.45**	**0.063**	**0.09**	**0.125**	**0.18**	**0.25**	**0.355**	**0.5**	**0.71**	**1**	**1.4**	**2**	**2.8**	**4**	**5.6**
1	37.70589	015.17334	0	0.1	0.214	0.77	1.23	1.22	1.872	4.262	6.348	5.255	3.284	1.519	0.359	0.149	0	0
2	37.72653	015.17353	0	0.042	0.087	0.224	0.41	0.6	2.252	4.942	4.826	2.786	1.306	0.506	0.138	0.052	0	0
3	37.73657	015.18671	0	0.122	0.234	0.378	0.5	0.69	0.895	0.946	2.014	2.732	1.901	1.038	0.618	0.315	0	0
4	37.74997	015.20913	0	0.041	0.12	0.407	0.47	0.74	0.807	1.703	2.334	1.427	0.551	0.333	0.504	0.571	0	0
5	37.75033	015.20819	0	0.003	0.09	0.281	0.26	0.37	0.571	1.842	3.384	2.06	0.801	0.309	0.093	0.035	0	0
6	37.75778	015.17101	0	0.097	0.321	0.612	0.44	0.53	0.477	0.709	0.647	0.349	0.152	0.07	0.011	0	0	0
7	37.74206	015.14308	0	0.082	0.197	0.411	0.55	0.67	0.799	2.069	4.972	5.281	4.371	2.934	1.931	1.329	0.453	0
8	37.73705	015.11236	0	0.322	0.654	1.366	2.7	3.4	5.78	14.864	34.258	30.854	21.656	14.5	8.872	6.86	3.654	0
9	37.72213	015.11852	0.01	0.038	0.112	0.375	0.97	2.02	2.579	5.258	9.172	11.248	8.758	5.871	2.781	1.143	0.347	0.266
10	37.70485	015.11537	0	0.135	0.236	0.379	0.5	0.29	0.083	0.04	0.148	0.41	0.841	1.071	1.114	0.889	0.383	0
11	37.71867	015.16584	0	0.054	0.128	0.378	1.82	2.69	3.562	8.094	13.505	8.047	3.282	1.151	0.294	0.089	0	0

**Table 2:** May 21 2016 deposit grain size data.

**Sample Number**	**GPS**		**Grain size (mm)**															
	**N**	**E**	**0.032**	**0.45**	**0.063**	**0.09**	**0.125**	**0.18**	**0.25**	**0.355**	**0.5**	**0.71**	**1**	**1.4**	**2**	**2.8**	**4**	**5.6**
1	37.51369	015.08199	0.122	0.064	0.083	0.216	0.28	0.7	0.646	0.18	0.027	0.007	0.004	0	0	0	0	0
2	37.54256	015.13931	0.009	0.018	0.02	0.034	0.08	0.6	2.14	2.153	0.956	0.218	0.051	0.027	0	0	0	0
3	37.5555	015.14852	0.017	0.027	0.03	0.042	0.06	0.35	1.622	3.35	2.891	0.99	0.34	0.071	0	0	0	0
4	37.55659	015.14851	0.001	0.005	0.007	0.013	0.04	0.25	1.263	2.762	2.383	0.751	0.21	0.033	0.003	0	0	0
5	37.56454	015.16251	0.002	0.011	0.015	0.021	0.02	0.02	0.075	0.269	0.593	0.424	0.203	0.052	0.019	0	0	0
6	37.60633	015.11188	0.028	0.024	0.017	0.013	0.02	0.01	0.029	0.133	0.326	0.29	0.153	0.037	0.02	0	0	0
7	37.60535	015.09850	0.047	0.038	0.049	0.061	0.08	0.15	0.936	3.528	7.626	6.347	3.332	1.494	0.373	0.082	0	0
8	37.64481	015.09222	0.012	0.047	0.079	0.069	0.06	0.04	0.055	0.116	0.438	0.554	0.333	0.237	0.112	0.044	0	0
9	37.62552	015.07937	0.019	0.142	0.119	0.178	0.08	0.14	0.701	2.952	5.915	4.831	2.766	1.23	0.427	0.092	0	0
10	37.61671	015.06844	0	0.006	0.093	0.142	0.09	0.34	1.702	3.586	3.016	1.686	0.553	0.148	0.073	0.004	0	0
11	37.60875	015.02958	0	0.089	0.119	0.14	0.12	0.25	0.447	0.283	0.137	0.006	0.001	0	0	0.033	0	0
12	37.68682	015.02307	0	0.204	0.327	0.503	0.56	0.97	2.144	5.511	8.092	8.179	5.268	2.692	1.192	0.709	0.106	0
14	37.69991	015.00049	0	0.09	0.063	0.078	0.04	0.07	0.141	0.106	0.095	0.007	0.001	0	0	0	0	0
15	37.69983	015.01597	0	0.212	0.315	0.421	0.47	0.83	1.523	2.798	3.951	3.957	1.918	1.093	0.423	0.256	0.092	0
16	37.65519	015.05367	0	0.129	0.148	0.181	0.13	0.25	1.254	5.242	12.492	11.239	5.436	4.815	1.672	0.601	0.1	0
17	37.57535	015.07484	0	0.387	0.399	0.376	0.11	0.54	2.187	2.792	1.387	0.339	0.077	0.019	0	0	0	0

## 2 Experimental design, materials, and methods

### 2.1 Sample preparation

Tephra samples were dried for 24 h at 105 °C prior to dry sieving at half phi intervals. The mass collected at each interval was measured using a Mettler PM100 precision balance. Grain size distribution data are presented in Tables 1 and 2.

Tephra and lava samples were prepared as carbon-coated 50 μm thin sections. Each section was first observed in a JEOL JSM-700IFA Scanning Electron Microscope to identify plagioclase, clinopyroxene and olivine phenocrysts for analysis. Melt inclusions greater than 10 μm and free from crystallisation or alteration within these phenocrysts were additionally identified.

### 2.2 Chemistry of mineral and glass phases

Electron microprobe analysis of the samples was undertaken using a JEOL JXA 8200 Superprobe at the University of Geneva. The operating conditions varied depending on the material analysed. For plagioclase, clinopyroxene and olivine phenocrysts the conditions used were: accelerating voltage of 10 kV; beam current of 15 nA, beam diameter of 3 μm; counting times of 8–20 s on peak positions and 4–10 s on background positions. For groundmass glass and melt inclusion the beam diameter was 10 μm and a defocused electron beam to limit loss of Cl, S and K. Elemental data are presented in Tables S1–S4.
