# Supplementary material for: Magma and tephra characteristics for the 17–25 May 2016 Mt Etna eruption
Source: Data Brief. 2018 Nov 26;22:65–71. doi: 10.1016/j.dib.2018.11.093 (PMC6297054; doi:10.1016/j.dib.2018.11.093)
Supplement: Supplementary file 2 — Supplementary material [file mmc2.docx]

| Analysis | SiO2 | Al2O3 | FeO | MnO | MgO | CaO | Na2O | K2O | SrO | Total | An | Ab | Or |
| --- | --- | --- | --- | --- | --- | --- | --- | --- | --- | --- | --- | --- | --- |
| Xenolith_01 | 46.05 | 34.21 | 0.4704 | - | 0.0067 | 18.21 | 1.2628 | 0.0571 | 0.1482 | 100.4151 | 88.56 | 11.11 | 0.33 |
| Xenolith_02 | 53.38 | 28.83 | 0.4724 | - | 0.0405 | 11.91 | 4.5 | 0.5247 | 0.3244 | 99.982 | 57.60 | 39.38 | 3.02 |
| Xenolith_03 | 53.67 | 28.78 | 0.5133 | - | 0.0608 | 11.74 | 4.47 | 0.5217 | 0.2778 | 100.0335 | 57.41 | 39.55 | 3.04 |
| Xenolith_04 | 46.33 | 33.68 | 0.4961 | - | 0.013 | 17.69 | 1.5 | 0.0797 | 0.1676 | 99.9565 | 86.30 | 13.24 | 0.46 |
| Xenolith_05 | 46.89 | 33.12 | 0.474 | - | 0.0039 | 17.02 | 1.6 | 0.0998 | 0.2783 | 99.486 | 84.95 | 14.45 | 0.59 |
| Xenolith_06 | 46.01 | 33.68 | 0.5228 | 0.0358 | 0.0103 | 17.66 | 1.3205 | 0.056 | 0.0568 | 99.3522 | 87.79 | 11.88 | 0.33 |
| Xenolith_07 | 46.33 | 33.59 | 0.5101 | 0.0147 | 0.0109 | 17.67 | 1.4 | 0.0752 | 0.2864 | 99.8874 | 87.07 | 12.48 | 0.44 |
| Xenolith_08 | 46.32 | 33.76 | 0.5175 | 0.0105 | 0.0377 | 17.63 | 1.3319 | 0.0729 | 0.0971 | 99.7776 | 87.59 | 11.98 | 0.43 |
| Xenolith_09 | 46.02 | 33.72 | 0.492 | 0.028 | 0.0213 | 17.62 | 1.3051 | 0.0665 | 0.3123 | 99.5853 | 87.83 | 11.77 | 0.39 |
| Xenolith_10 | 46.04 | 33.58 | 0.5253 | 0.0049 | 0.0119 | 17.8 | 1.2208 | 0.0673 | 0.2237 | 99.474 | 88.60 | 11.00 | 0.40 |
| Xenolith_11 | 46.01 | 33.86 | 0.4718 | 0.0183 | - | 17.83 | 1.38 | 0.0685 | 0.1142 | 99.7529 | 87.36 | 12.24 | 0.40 |
| Xenolith_12 | 46 | 33.7 | 0.48 | 0.0098 | 0.0213 | 17.8 | 1.3459 | 0.0569 | 0.0798 | 99.4938 | 87.67 | 12.00 | 0.33 |
| Xenolith_13 | 45.9 | 33.72 | 0.5121 | - | 0.021 | 17.76 | 1.285 | 0.0537 | 0.1933 | 99.4452 | 88.14 | 11.54 | 0.32 |
| Xenolith_14 | 45.96 | 33.8 | 0.4796 | - | 0.0092 | 17.91 | 1.23 | 0.06 | 0.2782 | 99.7271 | 88.63 | 11.01 | 0.35 |
| Xenolith_15 | 46.04 | 33.85 | 0.479 | 0.0168 | - | 17.78 | 1.2274 | 0.0541 | 0.0681 | 99.5154 | 88.61 | 11.07 | 0.32 |
| Xenolith_16 | 45.39 | 33.78 | 0.4996 | - | - | 17.84 | 1.1094 | 0.0508 | 0.209 | 98.8789 | 89.61 | 10.08 | 0.30 |
| Xenolith_17 | 45.91 | 33.73 | 0.5319 | 0.0196 | - | 17.62 | 1.1456 | 0.0541 | 0.1938 | 99.205 | 89.18 | 10.49 | 0.33 |
| Xenolith_18 | 45.41 | 33.97 | 0.4893 | 0.0161 | - | 17.91 | 1.0537 | 0.0664 | 0.2513 | 99.1668 | 90.02 | 9.58 | 0.40 |
| Xenolith_19 | 45.65 | 33.95 | 0.5053 | - | - | 17.94 | 1.0793 | 0.0483 | 0.2741 | 99.4471 | 89.92 | 9.79 | 0.29 |
| Xenolith_20 | 45.53 | 34.03 | 0.5054 | - | - | 18.08 | 1.0828 | 0.0545 | 0.174 | 99.4568 | 89.93 | 9.75 | 0.32 |
| Xenolith_21 | 45.67 | 33.82 | 0.5156 | - | - | 17.78 | 1.2449 | 0.0667 | 0.2081 | 99.3054 | 88.40 | 11.20 | 0.39 |
| Xenolith_22 | 45.97 | 33.69 | 0.5475 | 0.0056 | - | 17.69 | 1.2349 | 0.0687 | 0.0966 | 99.3034 | 88.42 | 11.17 | 0.41 |
| Xenolith_23 | 46.21 | 33.58 | 0.5784 | 0.0532 | - | 17.47 | 1.39 | 0.09 | 0.232 | 99.6037 | 86.95 | 12.52 | 0.53 |
| Xenolith_24 | 47.29 | 32.81 | 0.5182 | 0.0028 | 0.0053 | 16.7 | 2.02 | 0.1275 | 0.2448 | 99.7187 | 81.43 | 17.83 | 0.74 |
| Xenolith_25 | 47.45 | 32.94 | 0.5701 | 0.035 | - | 16.84 | 1.89 | 0.1199 | 0.1735 | 100.0184 | 82.54 | 16.76 | 0.70 |
| Xenolith_26 | 44.35 | 31.13 | 0.5222 | 0.0203 | 0.0005 | 16.26 | 1.6 | 0.105 | 0.2128 | 94.2009 | 84.33 | 15.02 | 0.65 |
| Xenolith_27 | 46.29 | 33.7 | 0.5417 | - | - | 17.69 | 1.35 | 0.0735 | 0.1831 | 99.8284 | 87.49 | 12.08 | 0.43 |
| Xenolith_28 | 46.16 | 33.63 | 0.5463 | - | - | 17.79 | 1.38 | 0.0916 | 0.0248 | 99.6228 | 87.22 | 12.24 | 0.53 |
| Xenolith_29 | 46.32 | 33.45 | 0.5528 | - | - | 17.39 | 1.52 | 0.1147 | 0.1714 | 99.519 | 85.76 | 13.57 | 0.67 |
| Xenolith_30 | 54.1 | 28.28 | 0.4753 | 0.0154 | 0.0391 | 11.31 | 4.78 | 0.6069 | 0.209 | 99.8158 | 54.68 | 41.82 | 3.49 |
| Xenolith_31 | 54.38 | 28.08 | 0.5373 | - | 0.0625 | 11.2 | 4.85 | 0.642 | 0.2362 | 99.9881 | 54.00 | 42.32 | 3.69 |

| Analysis | SiO2 | Al2O3 | FeO | MgO | CaO | Na2O | K2O | Total | An | Ab | Or |
| --- | --- | --- | --- | --- | --- | --- | --- | --- | --- | --- | --- |
| Tephra_01 | 47.06 | 32.4 | 0.6876 | 0.0735 | 16.89 | 2.1 | 0.143 | 99.3929 | 80.97 | 18.22 | 0.82 |
| Tephra_02 | 46.29 | 32.35 | 0.6648 | 0.0679 | 16.71 | 1.8 | 0.1263 | 98.0676 | 83.06 | 16.19 | 0.75 |
| Tephra_03 | 46.66 | 31.77 | 0.6644 | 0.0731 | 16.28 | 2.24 | 0.1694 | 97.8764 | 79.28 | 19.74 | 0.98 |
| Tephra_04 | 47.49 | 31.55 | 0.6658 | 0.0786 | 16.06 | 2.4 | 0.1796 | 98.4241 | 77.90 | 21.07 | 1.04 |
| Tephra_05 | 49.1 | 30.27 | 0.7189 | 0.1032 | 14.54 | 3.14 | 0.3091 | 98.3044 | 70.62 | 27.60 | 1.79 |
| Tephra_06 | 48.52 | 31.11 | 0.7577 | 0.1155 | 15.37 | 2.82 | 0.2517 | 98.9966 | 73.99 | 24.57 | 1.44 |
| Tephra_07 | 47.93 | 31.74 | 0.7177 | 0.0864 | 15.92 | 2.36 | 0.2087 | 98.9887 | 77.89 | 20.89 | 1.22 |
| Tephra_08 | 51.79 | 28.09 | 0.7901 | 0.118 | 12.01 | 4.35 | 0.505 | 97.8941 | 58.63 | 38.43 | 2.94 |
| Tephra_09 | 52.35 | 28.72 | 0.8192 | 0.0939 | 12.46 | 4.24 | 0.4717 | 99.3405 | 60.21 | 37.08 | 2.71 |
| Tephra_10 | 49.26 | 31.02 | 0.8697 | 0.0958 | 15.05 | 3 | 0.2637 | 99.6486 | 72.38 | 26.11 | 1.51 |
| Tephra_11 | 48.11 | 31.31 | 0.8476 | 0.1085 | 15.49 | 2.63 | 0.2372 | 98.8066 | 75.44 | 23.18 | 1.38 |
| Tephra_12 | 47.77 | 30.48 | 1.67 | 0.4043 | 15.4 | 2.55 | 0.3559 | 98.7029 | 75.35 | 22.58 | 2.07 |
| Tephra_13 | 52.78 | 27.84 | 0.7396 | 0.1063 | 11.61 | 4.67 | 0.5525 | 98.3951 | 56.04 | 40.79 | 3.18 |
| Tephra_14 | 47.87 | 31.33 | 0.61 | 0.1173 | 15.88 | 2.51 | 0.1743 | 98.6686 | 76.98 | 22.02 | 1.01 |
| Tephra_15 | 47.25 | 31.92 | 0.6015 | 0.0795 | 16.11 | 2.27 | 0.1477 | 98.4256 | 79.00 | 20.14 | 0.86 |
| Tephra_16 | 46.92 | 31.98 | 0.6166 | 0.0791 | 16.63 | 1.97 | 0.1133 | 98.3828 | 81.80 | 17.54 | 0.66 |
| Tephra_17 | 47.67 | 31.32 | 0.5915 | 0.0814 | 15.72 | 2.4 | 0.1682 | 98.1476 | 77.58 | 21.43 | 0.99 |
| Tephra_18 | 47.24 | 31.52 | 0.5814 | 0.079 | 16.11 | 2.27 | 0.1699 | 98.097 | 78.89 | 20.12 | 0.99 |
| Tephra_19 | 47.8 | 32.04 | 0.687 | 0.0857 | 16.19 | 2.23 | 0.1831 | 99.2401 | 79.19 | 19.74 | 1.07 |
| Tephra_20 | 47.93 | 31.76 | 0.6985 | 0.075 | 16.07 | 2.23 | 0.1719 | 98.9758 | 79.12 | 19.87 | 1.01 |
| Tephra_21 | 48.56 | 31.21 | 0.7578 | 0.1172 | 15.5 | 2.63 | 0.2544 | 99.046 | 75.38 | 23.15 | 1.47 |
| Tephra_22 | 49.07 | 30.56 | 0.6903 | 0.1112 | 14.72 | 2.94 | 0.2715 | 98.4259 | 72.29 | 26.13 | 1.59 |
| Tephra_23 | 49.52 | 30.9 | 0.6916 | 0.089 | 15.09 | 2.9 | 0.2471 | 99.4958 | 73.14 | 25.44 | 1.43 |
| Tephra_24 | 48.55 | 31.31 | 0.6862 | 0.0884 | 15.41 | 2.47 | 0.2182 | 98.7906 | 76.52 | 22.19 | 1.29 |
| Tephra_25 | 52.83 | 28.22 | 0.6697 | 0.1197 | 11.8 | 4.51 | 0.5137 | 98.7911 | 57.36 | 39.67 | 2.97 |
| Tephra_26 | 50.99 | 29.55 | 0.7571 | 0.1169 | 13.38 | 3.77 | 0.3612 | 99.0457 | 64.85 | 33.07 | 2.08 |
| Tephra_27 | 52.27 | 28.28 | 0.8122 | 0.1453 | 12.09 | 4.24 | 0.4984 | 98.5876 | 59.39 | 37.69 | 2.92 |
| Tephra_28 | 51.53 | 28.98 | 0.7029 | 0.1106 | 12.87 | 3.86 | 0.4092 | 98.5047 | 63.27 | 34.34 | 2.40 |
| Tephra_29 | 49.16 | 30.51 | 0.6527 | 0.0872 | 14.69 | 3.11 | 0.2651 | 98.5274 | 71.19 | 27.28 | 1.53 |
| Tephra_30 | 52.18 | 28.3 | 0.7159 | 0.1003 | 12.19 | 4.37 | 0.4997 | 98.4188 | 58.91 | 38.22 | 2.88 |
| Tephra_31 | 52.37 | 28.17 | 0.6759 | 0.1013 | 12.08 | 4.49 | 0.5152 | 98.4202 | 58.03 | 39.03 | 2.95 |
| Tephra_32 | 50.54 | 28.78 | 0.6749 | 0.1038 | 13.04 | 3.91 | 0.4111 | 97.5659 | 63.29 | 34.34 | 2.38 |
| Tephra_33 | 52.54 | 28.64 | 0.7596 | 0.1424 | 12.46 | 4.28 | 0.4367 | 99.3426 | 60.12 | 37.37 | 2.51 |
| Tephra_34 | 47.59 | 31.86 | 0.6436 | 0.0778 | 16.12 | 2.28 | 0.1767 | 98.7869 | 78.80 | 20.17 | 1.03 |
| Tephra_35 | 46.19 | 32.92 | 0.6701 | 0.0689 | 17.38 | 1.63 | 0.1175 | 99.0135 | 84.91 | 14.41 | 0.68 |
| Tephra_36 | 47.76 | 31.56 | 0.6588 | 0.0809 | 16.1 | 2.23 | 0.1692 | 98.5648 | 79.17 | 19.84 | 0.99 |
| Tephra_37 | 46.45 | 32.71 | 0.6333 | 0.0727 | 17.02 | 1.76 | 0.1301 | 98.7761 | 83.60 | 15.64 | 0.76 |
| Tephra_38 | 48.78 | 31.22 | 0.8003 | 0.1157 | 15.25 | 2.59 | 0.2443 | 99.0806 | 75.39 | 23.17 | 1.44 |
| Tephra_39 | 46.47 | 32.82 | 0.648 | 0.0695 | 17.01 | 1.7 | 0.1197 | 98.8645 | 84.09 | 15.21 | 0.70 |
| Tephra_40 | 47.32 | 31.97 | 0.7124 | 0.0672 | 16.31 | 2.12 | 0.1522 | 98.6648 | 80.24 | 18.87 | 0.89 |
| Tephra_41 | 52.99 | 28.09 | 0.6198 | 0.0794 | 11.75 | 4.59 | 0.5337 | 98.7255 | 56.79 | 40.14 | 3.07 |
| Tephra_42 | 51.84 | 28.85 | 0.6766 | 0.1066 | 12.67 | 4.08 | 0.4444 | 98.7659 | 61.56 | 35.87 | 2.57 |
| Tephra_43 | 51.57 | 28.55 | 0.6609 | 0.109 | 12.38 | 4.19 | 0.4693 | 98.0786 | 60.33 | 36.95 | 2.72 |
| Tephra_44 | 51.99 | 28.82 | 0.6808 | 0.113 | 12.44 | 4.33 | 0.4633 | 98.8372 | 59.73 | 37.62 | 2.65 |
| Tephra_45 | 46.71 | 32.35 | 0.693 | 0.0726 | 17.04 | 1.89 | 0.1459 | 98.927 | 82.58 | 16.58 | 0.84 |
| Analysis | SiO2 | Al2O3 | FeO | MgO | CaO | Na2O | K2O | Total | An | Ab | Or |
| Tephra_46 | 47.55 | 31.11 | 1.0053 | 0.2127 | 15.91 | 2.39 | 0.2551 | 98.4816 | 77.46 | 21.06 | 1.48 |
| Tephra_47 | 46.3 | 32.75 | 0.6313 | 0.0585 | 17.22 | 1.71 | 0.1147 | 98.8315 | 84.20 | 15.13 | 0.67 |
| Tephra_48 | 46.18 | 32.41 | 0.6816 | 0.0744 | 17.02 | 1.86 | 0.1232 | 98.3605 | 82.89 | 16.39 | 0.71 |
| Tephra_49 | 46.5 | 32.35 | 0.6407 | 0.0722 | 16.74 | 1.94 | 0.1371 | 98.3976 | 82.00 | 17.20 | 0.80 |
| Tephra_50 | 46.64 | 32.16 | 0.6424 | 0.0775 | 16.72 | 1.95 | 0.1474 | 98.3665 | 81.86 | 17.28 | 0.86 |
| Tephra_51 | 45.94 | 32.24 | 0.6709 | 0.1055 | 17.09 | 1.79 | 0.1183 | 98.01 | 83.49 | 15.82 | 0.69 |
| Tephra_52 | 46.25 | 32.55 | 0.6722 | 0.0546 | 17.11 | 1.66 | 0.1219 | 98.4528 | 84.46 | 14.83 | 0.72 |
| Tephra_53 | 46.42 | 31.95 | 0.6722 | 0.0736 | 16.76 | 1.97 | 0.13 | 97.9824 | 81.84 | 17.41 | 0.76 |
| Tephra_54 | 47.12 | 31.55 | 0.6559 | 0.0689 | 16.29 | 2.23 | 0.1818 | 98.0983 | 79.30 | 19.64 | 1.05 |
| Tephra_55 | 47.19 | 31.14 | 0.6576 | 0.0661 | 15.83 | 2.35 | 0.1973 | 97.4392 | 77.91 | 20.93 | 1.16 |
| Tephra_56 | 50.71 | 29.66 | 0.6956 | 0.084 | 13.56 | 3.71 | 0.3519 | 98.905 | 65.53 | 32.44 | 2.02 |
| Tephra_57 | 51.62 | 28.94 | 0.6966 | 0.0991 | 12.91 | 4.02 | 0.4208 | 98.803 | 62.41 | 35.17 | 2.42 |
| Tephra_58 | 51.23 | 29.13 | 0.717 | 0.089 | 13.08 | 3.95 | 0.3878 | 98.6745 | 63.22 | 34.55 | 2.23 |
| Tephra_59 | 51.86 | 28.6 | 0.7146 | 0.0955 | 12.46 | 4.25 | 0.4821 | 98.565 | 60.12 | 37.11 | 2.77 |
| Tephra_60 | 52.66 | 28.08 | 0.7274 | 0.1073 | 11.91 | 4.59 | 0.5055 | 98.637 | 57.21 | 39.90 | 2.89 |
| Tephra_61 | 51.51 | 28.89 | 0.733 | 0.1128 | 12.83 | 4.06 | 0.4064 | 98.6046 | 62.10 | 35.56 | 2.34 |
| Tephra_62 | 51.41 | 28.28 | 0.961 | 0.2106 | 12.47 | 4.32 | 0.4432 | 98.267 | 59.91 | 37.56 | 2.54 |
| Tephra_63 | 48.79 | 31.97 | 0.5444 | 0.083 | 16.07 | 2.56 | 0.1604 | 100.2755 | 76.91 | 22.17 | 0.91 |
| Tephra_64 | 48.4 | 31.81 | 0.5557 | 0.093 | 15.87 | 2.61 | 0.1652 | 99.6252 | 76.34 | 22.72 | 0.95 |
| Tephra_65 | 48.12 | 31.66 | 0.6268 | 0.0941 | 15.99 | 2.59 | 0.1645 | 99.344 | 76.61 | 22.45 | 0.94 |
| Tephra_66 | 48.82 | 31.14 | 0.6825 | 0.1031 | 15.51 | 2.84 | 0.2098 | 99.3555 | 74.21 | 24.59 | 1.20 |
| Tephra_67 | 49.07 | 31.14 | 0.7041 | 0.1074 | 15.4 | 2.87 | 0.226 | 99.5448 | 73.82 | 24.89 | 1.29 |
| Tephra_68 | 49.04 | 31.18 | 0.7078 | 0.0995 | 15.41 | 2.86 | 0.2148 | 99.5806 | 73.94 | 24.83 | 1.23 |
| Tephra_69 | 52.49 | 28.88 | 0.6625 | 0.1298 | 12.38 | 4.51 | 0.447 | 99.6794 | 58.75 | 38.73 | 2.53 |
| Tephra_70 | 52.55 | 28.82 | 0.715 | 0.0858 | 12.63 | 4.41 | 0.4552 | 99.756 | 59.71 | 37.73 | 2.56 |
| Tephra_71 | 47.39 | 32.65 | 0.6685 | 0.0763 | 16.77 | 1.99 | 0.1281 | 99.7069 | 81.71 | 17.55 | 0.74 |
| Tephra_72 | 47.04 | 32.61 | 0.6869 | 0.0563 | 16.92 | 1.92 | 0.1209 | 99.3659 | 82.38 | 16.92 | 0.70 |
| Tephra_73 | 47.06 | 32.65 | 0.664 | 0.0671 | 16.8 | 1.96 | 0.1294 | 99.3403 | 81.95 | 17.30 | 0.75 |
| Tephra_74 | 51.15 | 29.51 | 0.698 | 0.0809 | 13.34 | 3.95 | 0.3549 | 99.221 | 63.80 | 34.18 | 2.02 |
| Tephra_75 | 52.06 | 28.61 | 0.6625 | 0.1056 | 12.67 | 4.43 | 0.4727 | 99.1706 | 59.63 | 37.73 | 2.65 |
| Tephra_76 | 47.71 | 32.45 | 0.6471 | 0.0875 | 16.67 | 2.19 | 0.1517 | 99.9998 | 80.09 | 19.04 | 0.87 |
| Tephra_77 | 46.4 | 32.65 | 0.6517 | 0.0713 | 17.2 | 1.81 | 0.1336 | 98.9364 | 83.36 | 15.87 | 0.77 |
| Tephra_78 | 46.76 | 32.46 | 0.6734 | 0.0488 | 16.89 | 1.87 | 0.1166 | 98.855 | 82.74 | 16.58 | 0.68 |
| Tephra_79 | 46.83 | 32.62 | 0.6603 | 0.0614 | 17 | 1.92 | 0.1059 | 99.2329 | 82.52 | 16.87 | 0.61 |
| Tephra_80 | 47.01 | 32.77 | 0.7245 | 0.0586 | 17.2 | 1.86 | 0.1011 | 99.7418 | 83.15 | 16.27 | 0.58 |
| Tephra_81 | 51.77 | 28.8 | 0.7231 | 0.0939 | 12.77 | 4.25 | 0.4534 | 98.9243 | 60.81 | 36.62 | 2.57 |
| Tephra_82 | 53.09 | 28.33 | 0.7141 | 0.0992 | 12.02 | 4.59 | 0.5096 | 99.5432 | 57.42 | 39.68 | 2.90 |
| Tephra_83 | 48.49 | 32.15 | 0.7395 | 0.0878 | 16.18 | 2.45 | 0.2073 | 100.4093 | 77.56 | 21.25 | 1.18 |
| Tephra_84 | 50.33 | 30.62 | 0.6774 | 0.0875 | 14.54 | 3.2 | 0.2791 | 99.8082 | 70.37 | 28.02 | 1.61 |
| Tephra_85 | 52.49 | 29.19 | 0.7232 | 0.1124 | 12.8 | 4.13 | 0.4344 | 100.0837 | 61.57 | 35.95 | 2.49 |
| Tephra_86 | 50.72 | 30.26 | 0.7515 | 0.1006 | 14.13 | 3.47 | 0.3283 | 99.8252 | 67.93 | 30.19 | 1.88 |
| Tephra_87 | 51.46 | 30.11 | 0.8039 | 0.1222 | 13.66 | 3.77 | 0.3469 | 100.3606 | 65.37 | 32.65 | 1.98 |
| Tephra_88 | 51.65 | 29.79 | 0.7901 | 0.1212 | 13.46 | 3.77 | 0.3545 | 99.9922 | 65.01 | 32.95 | 2.04 |
| Tephra_89 | 45.95 | 31.57 | 0.6207 | 0.0683 | 16 | 1.91 | 0.1591 | 96.3121 | 81.44 | 17.59 | 0.96 |
| Tephra_90 | 48.37 | 32.38 | 0.6444 | 0.0586 | 16.03 | 2.37 | 0.1944 | 100.1409 | 78.00 | 20.87 | 1.13 |
| Tephra_91 | 47.99 | 32.6 | 0.6149 | 0.0684 | 16.35 | 2.16 | 0.148 | 100.0453 | 80.01 | 19.13 | 0.86 |
| Tephra_92 | 52.5 | 29.43 | 0.6132 | 0.0824 | 12.86 | 4.09 | 0.4094 | 100.0982 | 61.98 | 35.67 | 2.35 |
| Tephra_93 | 52.41 | 29.27 | 0.6123 | 0.0861 | 12.7 | 4.08 | 0.4427 | 99.7243 | 61.62 | 35.82 | 2.56 |
| Tephra_94 | 54.46 | 27.84 | 0.6096 | 0.0889 | 11.09 | 5.05 | 0.623 | 99.9216 | 52.88 | 43.58 | 3.54 |
| Tephra_95 | 53.47 | 28.58 | 0.6377 | 0.0878 | 11.96 | 4.47 | 0.508 | 99.9144 | 57.91 | 39.16 | 2.93 |
| Tephra_96 | 53.33 | 28.4 | 0.6329 | 0.092 | 11.86 | 4.51 | 0.5303 | 99.4391 | 57.43 | 39.52 | 3.06 |
| Tephra_97 | 52.54 | 29.27 | 0.6615 | 0.1137 | 12.75 | 4.21 | 0.3954 | 99.9923 | 61.18 | 36.56 | 2.26 |
| Tephra_98 | 47.94 | 32.31 | 0.6583 | 0.0777 | 16.29 | 2.19 | 0.1809 | 99.7882 | 79.59 | 19.36 | 1.05 |
| Tephra_99 | 50.13 | 30.58 | 0.7558 | 0.0775 | 14.33 | 3.27 | 0.3128 | 99.5825 | 69.50 | 28.70 | 1.81 |
| Tephra_100 | 52.63 | 28.68 | 0.6824 | 0.0844 | 12.3 | 4.41 | 0.5179 | 99.3596 | 58.86 | 38.19 | 2.95 |
| Tephra_101 | 51.05 | 29.89 | 0.7679 | 0.0967 | 13.51 | 3.67 | 0.3379 | 99.3511 | 65.73 | 32.31 | 1.96 |
| Tephra_102 | 47.59 | 32.7 | 0.7618 | 0.0688 | 16.8 | 1.94 | 0.1349 | 100.0593 | 82.07 | 17.15 | 0.78 |
| Tephra_103 | 52.95 | 29.34 | 0.7466 | 0.1018 | 12.47 | 4.18 | 0.5081 | 100.4582 | 60.42 | 36.65 | 2.93 |
| Tephra_104 | 47.23 | 32.45 | 0.7324 | 0.0509 | 16.71 | 2.02 | 0.1479 | 99.3413 | 81.35 | 17.80 | 0.86 |
| Tephra_105 | 53.19 | 28.63 | 0.7531 | 0.0985 | 12.04 | 4.42 | 0.5153 | 99.7836 | 58.30 | 38.73 | 2.97 |
| Tephra_106 | 52.67 | 29.01 | 0.7049 | 0.0902 | 12.51 | 4.3 | 0.475 | 99.8682 | 59.98 | 37.31 | 2.71 |
| Tephra_107 | 52.92 | 29.11 | 0.6795 | 0.1015 | 12.53 | 4.28 | 0.5063 | 100.2726 | 60.02 | 37.10 | 2.89 |
| Tephra_108 | 53.64 | 28.48 | 0.6914 | 0.1056 | 11.88 | 4.67 | 0.5462 | 100.1307 | 56.62 | 40.28 | 3.10 |
| Tephra_109 | 52.88 | 28.89 | 0.6942 | 0.079 | 12.33 | 4.49 | 0.5137 | 99.9573 | 58.53 | 38.57 | 2.90 |
| Tephra_110 | 52.76 | 29.01 | 0.655 | 0.1056 | 12.42 | 4.29 | 0.4983 | 99.7938 | 59.78 | 37.37 | 2.86 |
| Tephra_111 | 53.47 | 28.56 | 0.6676 | 0.0834 | 12.15 | 4.57 | 0.5268 | 100.1715 | 57.73 | 39.29 | 2.98 |
| Tephra_112 | 52.53 | 28.97 | 0.7024 | 0.0913 | 12.65 | 4.08 | 0.4843 | 99.6425 | 61.38 | 35.82 | 2.80 |
| Tephra_113 | 53.64 | 28.15 | 0.6889 | 0.0915 | 11.58 | 4.85 | 0.6055 | 99.7293 | 54.94 | 41.64 | 3.42 |
| Tephra_114 | 54.01 | 28.24 | 0.6198 | 0.0841 | 11.38 | 4.98 | 0.6156 | 100.0245 | 53.87 | 42.66 | 3.47 |
| Tephra_115 | 51.98 | 29.7 | 0.6299 | 0.091 | 13.09 | 4.09 | 0.4251 | 100.1793 | 62.34 | 35.25 | 2.41 |
| Tephra_116 | 53.62 | 28.32 | 0.6367 | 0.0824 | 11.73 | 4.69 | 0.6021 | 99.7215 | 56.03 | 40.54 | 3.42 |
| Tephra_117 | 53.66 | 28.53 | 0.613 | 0.0923 | 11.68 | 4.7 | 0.5822 | 99.8974 | 55.94 | 40.74 | 3.32 |
| Tephra_118 | 54.83 | 27.79 | 0.5855 | 0.0973 | 11.01 | 5.16 | 0.6799 | 100.2375 | 52.04 | 44.13 | 3.83 |
| Tephra_119 | 53.79 | 28.55 | 0.6182 | 0.0864 | 11.74 | 4.58 | 0.5774 | 99.9975 | 56.67 | 40.01 | 3.32 |
| Tephra_120 | 54.22 | 27.93 | 0.6492 | 0.0933 | 11.31 | 4.89 | 0.6201 | 99.922 | 54.12 | 42.35 | 3.53 |
| Tephra_121 | 53.26 | 29.06 | 0.6648 | 0.0968 | 12.43 | 4.36 | 0.4947 | 100.4436 | 59.45 | 37.73 | 2.82 |
| Tephra_122 | 53.44 | 28.87 | 0.6861 | 0.0864 | 12.16 | 4.57 | 0.5698 | 100.4378 | 57.61 | 39.18 | 3.21 |
| Tephra_123 | 47.26 | 31.98 | 0.7343 | 0.0854 | 16.19 | 2.25 | 0.1773 | 98.7465 | 79.08 | 19.89 | 1.03 |
| Tephra_124 | 46.75 | 31.91 | 0.6922 | 0.0707 | 16.47 | 2.05 | 0.155 | 98.1545 | 80.88 | 18.22 | 0.91 |
| Tephra_125 | 47.17 | 31.98 | 0.6809 | 0.0691 | 16.31 | 2.05 | 0.1627 | 98.4227 | 80.69 | 18.35 | 0.96 |
| Tephra_126 | 47.1 | 32.04 | 0.7573 | 0.0919 | 16.46 | 2.14 | 0.1829 | 98.8154 | 80.10 | 18.84 | 1.06 |
| Tephra_127 | 46.86 | 32.44 | 0.7266 | 0.0679 | 16.58 | 1.78 | 0.1356 | 98.6225 | 83.06 | 16.14 | 0.81 |
| Tephra_128 | 46.83 | 32.51 | 0.7323 | 0.0723 | 16.96 | 1.85 | 0.1321 | 99.1005 | 82.87 | 16.36 | 0.77 |
| Tephra_129 | 46.99 | 32.56 | 0.7224 | 0.0645 | 16.88 | 1.83 | 0.1323 | 99.2326 | 82.95 | 16.27 | 0.77 |
| Tephra_130 | 53.05 | 28.45 | 0.7504 | 0.11 | 11.88 | 4.45 | 0.5252 | 99.2689 | 57.79 | 39.17 | 3.04 |
| Tephra_131 | 47.62 | 31.79 | 0.6425 | 0.0226 | 15.87 | 1.95 | 0.1468 | 98.0546 | 81.08 | 18.03 | 0.89 |
| Tephra_132 | 48.3 | 31.4 | 0.7212 | 0.0232 | 15.41 | 2.21 | 0.2024 | 98.3045 | 78.42 | 20.35 | 1.23 |
| Tephra_133 | 48.05 | 31.53 | 0.6115 | 0.0118 | 15.62 | 2.06 | 0.1766 | 98.0709 | 79.86 | 19.06 | 1.08 |
| Tephra_134 | 48.13 | 31.41 | 0.6675 | 0.0181 | 15.58 | 2.13 | 0.1904 | 98.1605 | 79.24 | 19.60 | 1.15 |
| Tephra_135 | 47.82 | 31.7 | 0.5883 | 0.0033 | 15.95 | 1.99 | 0.1666 | 98.3114 | 80.76 | 18.23 | 1.00 |
| Tephra_136 | 47.09 | 32.33 | 0.5899 | - | 16.54 | 1.64 | 0.1324 | 98.3334 | 84.11 | 15.09 | 0.80 |
| Analysis | SiO2 | Al2O3 | FeO | MgO | CaO | Na2O | K2O | Total | An | Ab | Or |
| Tephra_137 | 47.36 | 31.89 | 0.638 | 0.0071 | 16.02 | 1.85 | 0.1578 | 98.003 | 81.92 | 17.12 | 0.96 |
| Tephra_138 | 53.03 | 28.08 | 0.5939 | 0.0174 | 11.49 | 4.43 | 0.5421 | 98.2179 | 57.02 | 39.78 | 3.20 |
| Tephra_139 | 49.71 | 30.26 | 0.7129 | 0.0075 | 14.24 | 3.17 | 0.3018 | 98.485 | 70.02 | 28.21 | 1.77 |
| Tephra_140 | 49.08 | 30.59 | 0.7195 | 0.0107 | 14.59 | 2.85 | 0.3162 | 98.2323 | 72.50 | 25.63 | 1.87 |
| Tephra_141 | 51.2 | 28.73 | 0.9314 | 0.1166 | 12.8 | 3.83 | 0.4554 | 98.1541 | 63.14 | 34.19 | 2.67 |
| Tephra_142 | 46.45 | 32.82 | 0.7055 | - | 17.05 | 1.65 | 0.1213 | 98.8377 | 84.49 | 14.80 | 0.72 |
| Tephra_143 | 48.16 | 31.25 | 0.6885 | 0.0092 | 14.92 | 2.73 | 0.2504 | 98.072 | 74.01 | 24.51 | 1.48 |
| Tephra_144 | 46.48 | 32.46 | 0.7092 | - | 16.37 | 1.89 | 0.1375 | 98.0935 | 82.04 | 17.14 | 0.82 |
| Tephra_145 | 46.08 | 32.56 | 0.7115 | - | 16.7 | 1.8 | 0.1154 | 98.046 | 83.11 | 16.21 | 0.68 |
| Tephra_146 | 52.37 | 28.4 | 0.7036 | 0.0136 | 11.79 | 4.29 | 0.5199 | 98.1693 | 58.45 | 38.48 | 3.07 |
| Tephra_147 | 52.4 | 28.46 | 0.6678 | 0.0305 | 11.57 | 4.4 | 0.5549 | 98.1775 | 57.30 | 39.43 | 3.27 |
| Tephra_148 | 50.11 | 30.75 | 1.3 | 0.0409 | 14.27 | 3.12 | 0.2903 | 99.8978 | 70.43 | 27.87 | 1.71 |
| Tephra_149 | 53.17 | 28.68 | 1.2797 | 0.0511 | 11.58 | 4.46 | 0.554 | 99.8033 | 57.01 | 39.74 | 3.25 |
| Tephra_150 | 53.03 | 28.65 | 1.2138 | 0.0534 | 11.84 | 4.32 | 0.5132 | 99.6674 | 58.42 | 38.57 | 3.01 |
| Tephra_151 | 46.31 | 33.52 | 1.0179 | - | 17.07 | 1.49 | 0.1065 | 99.5336 | 85.81 | 13.55 | 0.64 |
| Tephra_152 | 52.42 | 29.25 | 1.2426 | 0.0614 | 12.38 | 4.15 | 0.4484 | 100.0087 | 60.62 | 36.77 | 2.61 |
| Tephra_153 | 47.51 | 32.42 | 1.2552 | 0.0266 | 16.33 | 1.97 | 0.1118 | 99.631 | 81.54 | 17.80 | 0.66 |
| Tephra_154 | 49.88 | 30.57 | 1.2239 | 0.0402 | 14.33 | 3 | 0.2352 | 99.2931 | 71.51 | 27.09 | 1.40 |
| Tephra_155 | 47.3 | 32.78 | 1.2426 | - | 16.61 | 1.81 | 0.136 | 99.8857 | 82.85 | 16.34 | 0.81 |
| Tephra_156 | 53.2 | 28.53 | 1.2422 | 0.0692 | 11.77 | 4.42 | 0.5379 | 99.8143 | 57.67 | 39.19 | 3.14 |
| Tephra_157 | 51.23 | 29.28 | 2.8 | 0.2488 | 12.95 | 3.82 | 0.4604 | 100.8826 | 63.45 | 33.87 | 2.69 |
| Tephra_158 | 50.75 | 30.45 | 1.1802 | 0.0336 | 13.46 | 3.41 | 0.3706 | 99.6736 | 67.06 | 30.74 | 2.20 |
| Tephra_159 | 52 | 29.4 | 1.48 | 0.0658 | 12.52 | 4.08 | 0.4201 | 100.0474 | 61.36 | 36.19 | 2.45 |
| Tephra_160 | 48.34 | 32.33 | 1.2588 | 0.0128 | 15.78 | 2.15 | 0.1905 | 100.0752 | 79.31 | 19.55 | 1.14 |
| Tephra_161 | 52.19 | 29.25 | 1.1818 | 0.04 | 12.65 | 4.2 | 0.4572 | 100.0071 | 60.83 | 36.55 | 2.62 |
| Tephra_162 | 53.09 | 28.74 | 1.1761 | 0.0694 | 11.72 | 4.29 | 0.5088 | 99.6432 | 58.34 | 38.64 | 3.02 |
| Tephra_163 | 49.84 | 30.84 | 1.34 | 0.0373 | 13.93 | 3.18 | 0.3264 | 99.6026 | 69.40 | 28.67 | 1.94 |
| Tephra_164 | 53.34 | 28.62 | 1.2147 | 0.049 | 11.23 | 4.43 | 0.5884 | 99.5072 | 56.30 | 40.19 | 3.51 |
| Tephra_165 | 48.65 | 32.03 | 1.38 | 0.0181 | 14.93 | 2.56 | 0.21 | 99.812 | 75.36 | 23.38 | 1.26 |
| Tephra_166 | 52.94 | 29.1 | 1.2473 | 0.041 | 11.82 | 4.41 | 0.2973 | 99.8834 | 58.65 | 39.60 | 1.76 |
| Tephra_167 | 47.06 | 32.71 | 1.2722 | 0.0115 | 15.8 | 2.03 | 0.1564 | 99.0977 | 80.37 | 18.69 | 0.95 |
| Tephra_168 | 46.44 | 33.05 | 1.1475 | 0.0215 | 16.09 | 1.91 | 0.1454 | 98.8052 | 81.59 | 17.53 | 0.88 |
| Tephra_169 | 46.36 | 33.18 | 1.34 | - | 16.24 | 1.74 | 0.1355 | 99.0489 | 83.07 | 16.11 | 0.83 |
| Tephra_170 | 51.31 | 29.45 | 1.1734 | 0.0418 | 12.19 | 3.98 | 0.4409 | 98.5961 | 61.20 | 36.16 | 2.64 |
| Tephra_171 | 47.26 | 32.76 | 1.2549 | 0.0191 | 15.95 | 2.03 | 0.1577 | 99.4672 | 80.51 | 18.54 | 0.95 |
| Tephra_172 | 52.81 | 28.67 | 1.282 | 0.0547 | 11.38 | 4.29 | 0.5314 | 99.0525 | 57.54 | 39.26 | 3.20 |
| Tephra_173 | 52.88 | 28.44 | 1.0577 | 0.0494 | 10.89 | 4.57 | 0.5831 | 98.5501 | 54.85 | 41.65 | 3.50 |
| Tephra_174 | 47.98 | 32.61 | 1.2037 | - | 15.88 | 2.23 | 0.1693 | 100.1112 | 78.94 | 20.06 | 1.00 |
| Tephra_175 | 47.5 | 32.55 | 1.2556 | 0.0043 | 16.04 | 2.06 | 0.1498 | 99.6199 | 80.42 | 18.69 | 0.89 |
| Tephra_176 | 48.22 | 31.83 | 1.2342 | 0.0108 | 15.04 | 2.51 | 0.2017 | 99.0952 | 75.87 | 22.91 | 1.21 |
| Tephra_177 | 48.78 | 31.86 | 1.2548 | 0.0078 | 15.05 | 2.51 | 0.2183 | 99.7131 | 75.81 | 22.88 | 1.31 |
| Tephra_178 | 47.91 | 32.42 | 1.2212 | 0.0105 | 15.73 | 2.26 | 0.1762 | 99.7299 | 78.53 | 20.42 | 1.05 |
| Tephra_179 | 47.81 | 32.52 | 1.3 | - | 15.86 | 2.18 | 0.1694 | 99.908 | 79.27 | 19.72 | 1.01 |
| Tephra_180 | 52.02 | 29.72 | 1.1849 | 0.0456 | 12.58 | 4.01 | 0.4184 | 100.0203 | 61.86 | 35.69 | 2.45 |
| Tephra_181 | 48.25 | 32.07 | 1.26 | 0.013 | 15.41 | 2.43 | 0.1962 | 99.6497 | 76.89 | 21.94 | 1.17 |
| Analysis | SiO2 | Al2O3 | FeO | MgO | CaO | Na2O | K2O | Total | An | Ab | Or |
| Tephra_182 | 49.73 | 31.27 | 1.2071 | 0.0293 | 14.57 | 2.94 | 0.2573 | 100.0406 | 72.14 | 26.34 | 1.52 |
| Tephra_183 | 48.74 | 31.95 | 1.1691 | 0.0132 | 15.33 | 2.57 | 0.2196 | 100.0058 | 75.73 | 22.98 | 1.29 |
| Tephra_184 | 48.89 | 31.95 | 1.2668 | 0.0276 | 15.04 | 2.62 | 0.2165 | 100.051 | 75.05 | 23.66 | 1.29 |
| Tephra_185 | 50.05 | 30.66 | 1.1978 | 0.025 | 13.99 | 2.75 | 0.2753 | 98.9974 | 72.51 | 25.79 | 1.70 |
| Tephra_186 | 49.85 | 31.24 | 1.2696 | 0.0251 | 14.18 | 2.86 | 0.2715 | 99.7196 | 72.06 | 26.30 | 1.64 |
| Tephra_187 | 49.09 | 31.02 | 1.2139 | 0.0028 | 14.49 | 2.56 | 0.2508 | 98.6731 | 74.61 | 23.85 | 1.54 |
| Tephra_188 | 49.55 | 31.04 | 1.2166 | 0.0205 | 14.31 | 2.76 | 0.2598 | 99.2013 | 72.96 | 25.46 | 1.58 |
| Tephra_189 | 48.51 | 31.65 | 1.2102 | 0 | 15.03 | 2.35 | 0.1892 | 98.9844 | 77.05 | 21.80 | 1.15 |
| Tephra_190 | 48.67 | 31.67 | 1.1913 | 0.0215 | 15.09 | 2.3 | 0.2144 | 99.172 | 77.36 | 21.34 | 1.31 |
| Tephra_191 | 48.31 | 32.15 | 1.2777 | 0 | 15.44 | 2.19 | 0.1841 | 99.6175 | 78.69 | 20.20 | 1.12 |
| Tephra_192 | 47.63 | 32.51 | 1.254 | 0.0112 | 15.85 | 2.03 | 0.166 | 99.4761 | 80.37 | 18.63 | 1.00 |
| Tephra_193 | 47.95 | 30.97 | 3.14 | 0.3092 | 14.81 | 2.32 | 0.4128 | 100.002 | 75.95 | 21.53 | 2.52 |
| Tephra_194 | 48.6 | 31.85 | 1.0931 | 0.0165 | 15.19 | 2.26 | 0.1914 | 99.2011 | 77.87 | 20.96 | 1.17 |
| Tephra_195 | 47.55 | 32.64 | 1.1785 | 0.007 | 16.14 | 1.94 | 0.1559 | 99.6316 | 81.37 | 17.70 | 0.94 |
| Tephra_196 | 47.8 | 32.38 | 1.1428 | 0.0178 | 15.69 | 2.07 | 0.1549 | 99.2577 | 79.97 | 19.09 | 0.94 |
| Tephra_197 | 48.11 | 32.08 | 1.1259 | 0.0152 | 15.6 | 2.17 | 0.1649 | 99.3074 | 79.09 | 19.91 | 1.00 |
| Tephra_198 | 47.73 | 32.59 | 1.2089 | 0.0098 | 15.79 | 2.04 | 0.1619 | 99.5683 | 80.26 | 18.76 | 0.98 |
| Tephra_199 | 48.1 | 32.14 | 1.2231 | - | 15.61 | 2.18 | 0.1718 | 99.4335 | 79.00 | 19.96 | 1.04 |
| Tephra_200 | 47.76 | 32.32 | 1.2283 | 0.0105 | 15.7 | 2.13 | 0.1684 | 99.3173 | 79.47 | 19.51 | 1.01 |
| Tephra_201 | 47.91 | 32.28 | 1.1563 | 0.0133 | 15.65 | 2.06 | 0.1648 | 99.2729 | 79.95 | 19.04 | 1.00 |
| Tephra_202 | 47.18 | 32.7 | 1.1841 | 0.0174 | 16.08 | 1.84 | 0.133 | 99.157 | 82.17 | 17.02 | 0.81 |
| Tephra_203 | 49.26 | 31.42 | 1.2147 | 0.0172 | 15.17 | 2.51 | 0.2308 | 99.8276 | 75.90 | 22.73 | 1.37 |
| Tephra_204 | 48.72 | 32 | 1.2037 | 0.0195 | 15.54 | 2.23 | 0.2197 | 99.9602 | 78.34 | 20.34 | 1.32 |
| Tephra_205 | 47.02 | 33.15 | 1.2302 | 0.0167 | 17.01 | 1.51 | 0.1273 | 100.1124 | 85.50 | 13.74 | 0.76 |
| Tephra_206 | 48.14 | 32.38 | 1.1684 | - | 16.08 | 1.91 | 0.1779 | 99.9075 | 81.43 | 17.50 | 1.07 |
| Tephra_207 | 48.93 | 31.88 | 1.2861 | 0.0005 | 15.46 | 2.19 | 0.205 | 99.9985 | 78.61 | 20.15 | 1.24 |
| Tephra_208 | 53.62 | 28.49 | 1.2064 | 0.0457 | 11.66 | 3.9 | 0.5565 | 99.535 | 60.16 | 36.42 | 3.42 |
| Tephra_209 | 54.05 | 28.12 | 1.1529 | 0.0612 | 11.15 | 4.1 | 0.6174 | 99.3141 | 57.76 | 38.43 | 3.81 |
| Tephra_210 | 53.2 | 28.56 | 1.1434 | 0.0395 | 11.72 | 3.81 | 0.5221 | 99.0578 | 60.93 | 35.84 | 3.23 |

| analysis | SiO2 | Al2O3 | FeO | MgO | CaO | Na2O | K2O | Total | An | Ab | Or |
| --- | --- | --- | --- | --- | --- | --- | --- | --- | --- | --- | --- |
| STROMBOLIAN_1 | 47.19 | 33 | 0.6113 | 0.0383 | 17.08 | 1.74 | 0.1184 | 99.8299 | 83.85 | 15.46 | 0.69 |
| STROMBOLIAN_2 | 46.99 | 33.27 | 0.6153 | 0.0407 | 17.22 | 1.65 | 0.124 | 99.9531 | 84.60 | 14.67 | 0.73 |
| STROMBOLIAN_3 | 47.79 | 32.88 | 0.5941 | 0.0449 | 16.61 | 2.12 | 0.1598 | 100.273 | 80.49 | 18.59 | 0.92 |
| STROMBOLIAN_4 | 51.55 | 29.22 | 0.7363 | 0.0547 | 12.84 | 3.99 | 0.4448 | 99.0003 | 62.36 | 35.07 | 2.57 |
| STROMBOLIAN_5 | 49.73 | 31.15 | 0.7499 | 0.0745 | 14.95 | 2.96 | 0.2813 | 99.9974 | 72.43 | 25.95 | 1.62 |
| STROMBOLIAN_6 | 46.78 | 32.69 | 0.6411 | 0.057 | 16.96 | 1.82 | 0.1266 | 99.1475 | 83.12 | 16.14 | 0.74 |
| STROMBOLIAN_7 | 47.14 | 33.28 | 0.6725 | 0.0385 | 17.04 | 1.79 | 0.1262 | 100.1844 | 83.41 | 15.86 | 0.74 |
| STROMBOLIAN_8 | 48.35 | 32.02 | 0.7128 | 0.0681 | 16.07 | 2.38 | 0.1774 | 99.8305 | 78.05 | 20.92 | 1.03 |
| STROMBOLIAN_9 | 53.81 | 28.38 | 0.6366 | 0.0928 | 11.66 | 4.76 | 0.5445 | 100.0142 | 55.73 | 41.17 | 3.10 |
| STROMBOLIAN_10 | 49.06 | 30.62 | 0.7505 | 0.0942 | 14.79 | 3.07 | 0.2786 | 98.7256 | 71.53 | 26.87 | 1.60 |
| STROMBOLIAN_11 | 51.97 | 28.84 | 0.6544 | 0.0692 | 12.38 | 4.19 | 0.4565 | 98.7692 | 60.37 | 36.98 | 2.65 |
| STROMBOLIAN_12 | 50.87 | 29.17 | 0.7699 | 0.1157 | 13.33 | 3.8 | 0.3542 | 98.4689 | 64.62 | 33.34 | 2.04 |
| STROMBOLIAN_13 | 48.61 | 31.34 | 0.7607 | 0.0909 | 15.46 | 2.57 | 0.2076 | 99.0884 | 75.94 | 22.84 | 1.21 |
| STROMBOLIAN_14 | 47.54 | 31.7 | 0.7783 | 0.0994 | 16.05 | 2.32 | 0.1657 | 98.6583 | 78.50 | 20.53 | 0.96 |
| STROMBOLIAN_15 | 52.34 | 28.1 | 0.7925 | 0.1063 | 12.28 | 4.38 | 0.5161 | 98.5775 | 58.98 | 38.07 | 2.95 |
| STROMBOLIAN_16 | 52.88 | 27.75 | 0.8081 | 0.0999 | 11.72 | 4.54 | 0.6055 | 98.4983 | 56.74 | 39.77 | 3.49 |
| STROMBOLIAN_17 | 47.24 | 31.71 | 0.8323 | 0.0729 | 16.19 | 2.21 | 0.1644 | 98.4357 | 79.42 | 19.62 | 0.96 |
| STROMBOLIAN_18 | 48.1 | 31.06 | 0.7643 | 0.0976 | 15.54 | 2.6 | 0.1898 | 98.4596 | 75.91 | 22.98 | 1.10 |
| STROMBOLIAN_19 | 48.19 | 30.82 | 0.9983 | 0.1792 | 15.46 | 2.7 | 0.2235 | 98.624 | 75.00 | 23.70 | 1.29 |
| STROMBOLIAN_20 | 48.67 | 31.67 | 1.1608 | 0.2347 | 15.9 | 2.46 | 0.2777 | 100.4841 | 76.88 | 21.52 | 1.60 |
| STROMBOLIAN_21 | 48.02 | 31.87 | 0.8228 | 0.0924 | 16.31 | 2.22 | 0.171 | 99.537 | 79.44 | 19.57 | 0.99 |
| STROMBOLIAN_22 | 48.23 | 30.96 | 0.8197 | 0.0831 | 15.3 | 2.7 | 0.2136 | 98.3194 | 74.85 | 23.90 | 1.24 |
| STROMBOLIAN_23 | 47.52 | 31.46 | 0.8914 | 0.0787 | 15.92 | 2.39 | 0.1907 | 98.4509 | 77.76 | 21.13 | 1.11 |
| STROMBOLIAN_24 | 50.24 | 29.62 | 0.8574 | 0.1098 | 13.72 | 3.47 | 0.3538 | 98.4254 | 67.19 | 30.75 | 2.06 |
| STROMBOLIAN_25 | 52.15 | 28.4 | 0.8317 | 0.11 | 12.4 | 4.37 | 0.4643 | 98.8406 | 59.44 | 37.91 | 2.65 |
| STROMBOLIAN_26 | 51.99 | 28.22 | 0.8869 | 0.2009 | 12.17 | 4.42 | 0.521 | 98.5311 | 58.54 | 38.47 | 2.98 |
| STROMBOLIAN_27 | 46.59 | 32.59 | 0.7229 | 0.0612 | 16.87 | 1.78 | 0.1243 | 98.7612 | 83.35 | 15.92 | 0.73 |
| STROMBOLIAN_28 | 52.68 | 28.45 | 0.6992 | 0.0813 | 11.96 | 4.39 | 0.5463 | 98.9459 | 58.19 | 38.65 | 3.16 |
| STROMBOLIAN_29 | 53.16 | 28.21 | 0.7036 | 0.0964 | 11.85 | 4.43 | 0.5523 | 99.1525 | 57.74 | 39.06 | 3.20 |
| STROMBOLIAN_30 | 47.43 | 31.94 | 0.7033 | 0.0595 | 16.24 | 2.19 | 0.1741 | 98.7772 | 79.57 | 19.42 | 1.02 |
| STROMBOLIAN_31 | 47.88 | 31.76 | 0.66 | 0.0651 | 16.18 | 2.33 | 0.185 | 99.0968 | 78.48 | 20.45 | 1.07 |
| STROMBOLIAN_32 | 47.5 | 31.86 | 0.6768 | 0.0667 | 16.1 | 2.29 | 0.1816 | 98.6752 | 78.69 | 20.25 | 1.06 |
| STROMBOLIAN_33 | 47.5 | 31.82 | 0.6993 | 0.0641 | 16.21 | 2.2 | 0.1755 | 98.7216 | 79.46 | 19.52 | 1.02 |
| STROMBOLIAN_34 | 47.85 | 31.66 | 0.649 | 0.0639 | 15.88 | 2.28 | 0.1945 | 98.6126 | 78.47 | 20.39 | 1.14 |
| STROMBOLIAN_35 | 47.91 | 31.44 | 0.7 | 0.0817 | 15.85 | 2.39 | 0.208 | 98.7235 | 77.61 | 21.18 | 1.21 |
| STROMBOLIAN_36 | 48.67 | 31.1 | 0.6604 | 0.0703 | 15.33 | 2.69 | 0.2465 | 98.8706 | 74.81 | 23.76 | 1.43 |
| STROMBOLIAN_37 | 49.38 | 30.65 | 0.6888 | 0.0717 | 14.68 | 2.98 | 0.2714 | 98.7726 | 71.98 | 26.44 | 1.58 |
| STROMBOLIAN_38 | 53.38 | 27.64 | 0.6614 | 0.0861 | 11.32 | 4.8 | 0.6582 | 98.796 | 54.45 | 41.78 | 3.77 |
| STROMBOLIAN_39 | 53.61 | 27.69 | 0.6603 | 0.0781 | 11.23 | 4.75 | 0.661 | 98.8401 | 54.48 | 41.70 | 3.82 |
| STROMBOLIAN_40 | 54.1 | 27.32 | 0.649 | 0.0989 | 10.85 | 5.01 | 0.7241 | 98.7962 | 52.22 | 43.63 | 4.15 |
| STROMBOLIAN_41 | 53.77 | 27.39 | 0.6979 | 0.1058 | 10.97 | 4.92 | 0.6982 | 98.8101 | 52.98 | 43.00 | 4.02 |
| STROMBOLIAN_42 | 53.9 | 27.52 | 0.7037 | 0.0983 | 11.04 | 4.95 | 0.6817 | 99.021 | 53.05 | 43.05 | 3.90 |
| STROMBOLIAN_43 | 53.39 | 27.75 | 0.6739 | 0.0994 | 11.44 | 4.84 | 0.6318 | 98.8954 | 54.60 | 41.81 | 3.59 |
| STROMBOLIAN_44 | 52.6 | 28.36 | 0.7417 | 0.1075 | 12 | 4.39 | 0.5351 | 98.858 | 58.31 | 38.60 | 3.10 |
| STROMBOLIAN_45 | 52.62 | 27.64 | 1.29 | 0.2666 | 11.78 | 4.51 | 0.6864 | 98.9699 | 56.75 | 39.32 | 3.94 |
| analysis | SiO2 | Al2O3 | FeO | MgO | CaO | Na2O | K2O | Total | An | Ab | Or |
| STROMBOLIAN_46 | 44.84 | 33.48 | 0.6095 | 0.0494 | 18.16 | 1.1064 | 0.0731 | 98.3507 | 89.68 | 9.89 | 0.43 |
| STROMBOLIAN_47 | 46.56 | 32.41 | 0.6084 | 0.0558 | 16.79 | 1.79 | 0.1437 | 98.4025 | 83.12 | 16.04 | 0.85 |
| STROMBOLIAN_48 | 45.58 | 33.13 | 0.6174 | 0.0619 | 17.49 | 1.45 | 0.0958 | 98.4543 | 86.46 | 12.97 | 0.56 |
| STROMBOLIAN_49 | 46.78 | 32.25 | 0.6378 | 0.0602 | 16.79 | 2.01 | 0.1456 | 98.7113 | 81.50 | 17.66 | 0.84 |
| STROMBOLIAN_50 | 46.7 | 32.4 | 0.5843 | 0.0644 | 16.9 | 1.88 | 0.1258 | 98.7014 | 82.63 | 16.63 | 0.73 |
| STROMBOLIAN_51 | 46.57 | 32.32 | 0.5777 | 0.0549 | 16.64 | 1.92 | 0.1347 | 98.2432 | 82.07 | 17.14 | 0.79 |
| STROMBOLIAN_52 | 45.78 | 33.2 | 0.5797 | 0.0551 | 17.45 | 1.46 | 0.0856 | 98.6397 | 86.41 | 13.08 | 0.50 |
| STROMBOLIAN_53 | 45.84 | 32.96 | 0.5904 | 0.0541 | 17.51 | 1.51 | 0.0928 | 98.5574 | 86.03 | 13.43 | 0.54 |
| STROMBOLIAN_54 | 47.68 | 31.61 | 0.6347 | 0.0711 | 15.91 | 2.4 | 0.1663 | 98.5015 | 77.80 | 21.24 | 0.97 |
| STROMBOLIAN_55 | 46.7 | 32.24 | 0.7099 | 0.0602 | 16.73 | 1.88 | 0.1266 | 98.4597 | 82.48 | 16.77 | 0.74 |
| STROMBOLIAN_56 | 47.04 | 32.09 | 0.7407 | 0.0846 | 16.58 | 2 | 0.1332 | 98.6686 | 81.44 | 17.78 | 0.78 |
| STROMBOLIAN_57 | 49.01 | 31.2 | 0.7685 | 0.082 | 15.41 | 2.74 | 0.2298 | 99.4633 | 74.65 | 24.02 | 1.33 |
| STROMBOLIAN_58 | 45.56 | 33.69 | 0.5904 | 0.0532 | 17.94 | 1.2725 | 0.0762 | 99.1824 | 88.23 | 11.32 | 0.45 |
| STROMBOLIAN_59 | 45.64 | 33.28 | 0.6223 | 0.0609 | 17.76 | 1.43 | 0.0766 | 98.8699 | 86.89 | 12.66 | 0.45 |
| STROMBOLIAN_60 | 45.61 | 33.4 | 0.5736 | 0.0628 | 17.84 | 1.3384 | 0.0809 | 98.909 | 87.63 | 11.90 | 0.47 |
| STROMBOLIAN_61 | 45.4 | 33.71 | 0.5898 | 0.0559 | 18.23 | 1.1259 | 0.0682 | 99.214 | 89.59 | 10.01 | 0.40 |
| STROMBOLIAN_62 | 45.32 | 33.67 | 0.5387 | 0.0517 | 18.04 | 1.1566 | 0.0608 | 98.8753 | 89.28 | 10.36 | 0.36 |
| STROMBOLIAN_63 | 45.47 | 33.47 | 0.6205 | 0.0502 | 17.84 | 1.2763 | 0.0772 | 98.8419 | 88.14 | 11.41 | 0.45 |
| STROMBOLIAN_64 | 45.33 | 33.5 | 0.5621 | 0.0446 | 17.88 | 1.2176 | 0.0848 | 98.6256 | 88.58 | 10.92 | 0.50 |
| STROMBOLIAN_65 | 45.34 | 33.65 | 0.5984 | 0.0472 | 18.25 | 1.2575 | 0.0583 | 99.229 | 88.61 | 11.05 | 0.34 |
| STROMBOLIAN_66 | 45.41 | 33.62 | 0.6153 | 0.0354 | 17.98 | 1.3222 | 0.073 | 99.0625 | 87.88 | 11.69 | 0.42 |
| STROMBOLIAN_67 | 45.85 | 33.3 | 0.5931 | 0.0606 | 17.73 | 1.47 | 0.0835 | 99.2012 | 86.53 | 12.98 | 0.49 |
| STROMBOLIAN_68 | 45.05 | 33.68 | 0.5812 | 0.0582 | 18.34 | 1.0892 | 0.0597 | 98.8843 | 89.98 | 9.67 | 0.35 |
| STROMBOLIAN_69 | 45.12 | 33.75 | 0.6266 | 0.0472 | 18.25 | 1.0474 | 0.0565 | 98.9809 | 90.29 | 9.38 | 0.33 |
| STROMBOLIAN_70 | 45.56 | 34.25 | 0.5964 | 0.0493 | 18.31 | 1.1212 | 0.0583 | 99.9958 | 89.72 | 9.94 | 0.34 |
| STROMBOLIAN_71 | 48.23 | 32.25 | 0.6825 | 0.084 | 16.44 | 2.23 | 0.1647 | 100.0812 | 79.53 | 19.52 | 0.95 |
| STROMBOLIAN_72 | 48.55 | 32.09 | 0.7267 | 0.0971 | 15.93 | 2.46 | 0.1773 | 100.031 | 77.36 | 21.62 | 1.03 |
| STROMBOLIAN_73 | 48.47 | 32.13 | 0.6591 | 0.0814 | 16.01 | 2.44 | 0.1589 | 100.0203 | 77.66 | 21.42 | 0.92 |
| STROMBOLIAN_74 | 48.34 | 32.09 | 0.6596 | 0.0795 | 16.06 | 2.35 | 0.1693 | 99.7646 | 78.29 | 20.73 | 0.98 |
| STROMBOLIAN_75 | 48.26 | 32.23 | 0.7307 | 0.0838 | 16.17 | 2.21 | 0.1502 | 99.8975 | 79.47 | 19.65 | 0.88 |
| STROMBOLIAN_76 | 48.45 | 30.76 | 1.37 | 0.41 | 15.8 | 2.42 | 0.4456 | 99.6832 | 76.29 | 21.15 | 2.56 |
| STROMBOLIAN_77 | 47.42 | 32.75 | 0.752 | 0.0589 | 16.92 | 1.91 | 0.1194 | 99.9499 | 82.46 | 16.85 | 0.69 |
| STROMBOLIAN_78 | 47.81 | 32.36 | 0.7895 | 0.0698 | 16.56 | 2.21 | 0.1573 | 99.9987 | 79.82 | 19.28 | 0.90 |
| STROMBOLIAN_79 | 53.74 | 28.42 | 0.7992 | 0.1192 | 11.69 | 4.73 | 0.5407 | 100.0923 | 55.95 | 40.97 | 3.08 |
| STROMBOLIAN_80 | 51.56 | 29.06 | 0.8531 | 0.0933 | 12.78 | 4.24 | 0.44 | 99.1875 | 60.92 | 36.58 | 2.50 |
| STROMBOLIAN_81 | 51.2 | 30.02 | 0.9928 | 0.1177 | 13.63 | 3.67 | 0.3894 | 100.0673 | 65.73 | 32.03 | 2.24 |
| STROMBOLIAN_82 | 52.99 | 29.08 | 0.938 | 0.1247 | 12.44 | 4.21 | 0.4967 | 100.4588 | 60.24 | 36.89 | 2.86 |
| STROMBOLIAN_83 | 51.4 | 28.68 | 1.0677 | 0.1204 | 12.83 | 4.07 | 0.4773 | 98.7661 | 61.79 | 35.47 | 2.74 |
| STROMBOLIAN_84 | 51.95 | 28.69 | 0.9792 | 0.1184 | 12.57 | 4.13 | 0.4754 | 98.9775 | 60.99 | 36.26 | 2.75 |
| STROMBOLIAN_85 | 53.2 | 27.2 | 0.9325 | 0.1422 | 11.13 | 4.88 | 0.6266 | 98.3356 | 53.75 | 42.65 | 3.60 |
| STROMBOLIAN_86 | 52.99 | 28.08 | 1.0136 | 0.1229 | 11.63 | 4.47 | 0.6013 | 99.2019 | 56.91 | 39.58 | 3.50 |
| STROMBOLIAN_87 | 44.57 | 34.61 | 1.38 | - | 17.98 | 0.9734 | 0.0758 | 99.5909 | 90.66 | 8.88 | 0.46 |
| STROMBOLIAN_88 | 44.84 | 34.32 | 1.2613 | - | 17.62 | 1.2269 | 0.08 | 99.3483 | 88.39 | 11.14 | 0.48 |
| STROMBOLIAN_89 | 46.73 | 33.69 | 1.2068 | - | 17.32 | 1.55 | 0.1051 | 100.6342 | 85.53 | 13.85 | 0.62 |
| STROMBOLIAN_90 | 46.73 | 33.63 | 1.2135 | - | 17.26 | 1.6 | 0.1058 | 100.5473 | 85.10 | 14.28 | 0.62 |
| analysis | SiO2 | Al2O3 | FeO | MgO | CaO | Na2O | K2O | Total | An | Ab | Or |
| STROMBOLIAN_91 | 45.4 | 34.38 | 1.36 | - | 18.14 | 1.0367 | 0.0805 | 100.3971 | 90.20 | 9.33 | 0.48 |
| STROMBOLIAN_92 | 47.38 | 32.77 | 0.5219 | - | 16.59 | 2.17 | 0.1381 | 99.8443 | 80.22 | 18.99 | 0.80 |
| STROMBOLIAN_93 | 45.81 | 33.57 | 0.5197 | - | 17.43 | 1.55 | 0.089 | 99.1432 | 85.69 | 13.79 | 0.52 |
| STROMBOLIAN_94 | 45.31 | 33.81 | 0.5234 | 0.0007 | 17.55 | 1.3228 | 0.0921 | 98.7558 | 87.52 | 11.94 | 0.55 |
| STROMBOLIAN_95 | 45.05 | 33.41 | 0.5989 | 0.0044 | 17.59 | 1.4 | 0.1404 | 98.3706 | 86.69 | 12.49 | 0.82 |
| STROMBOLIAN_96 | 45.03 | 33.63 | 0.4917 | 0.0001 | 17.62 | 1.42 | 0.076 | 98.4603 | 86.88 | 12.67 | 0.45 |
| STROMBOLIAN_97 | 46.19 | 32.84 | 0.4585 | 0.0124 | 16.56 | 1.9 | 0.1222 | 98.3442 | 82.21 | 17.07 | 0.72 |
| STROMBOLIAN_98 | 45.72 | 33.2 | 0.5025 | - | 17.06 | 1.61 | 0.1075 | 98.4063 | 84.87 | 14.49 | 0.64 |
| STROMBOLIAN_99 | 45.56 | 33.54 | 0.4757 | 0.0088 | 17.39 | 1.46 | 0.0891 | 98.7888 | 86.35 | 13.12 | 0.53 |
| STROMBOLIAN_100 | 45.64 | 33.54 | 0.5085 | 0.0314 | 17.28 | 1.49 | 0.1008 | 98.822 | 85.99 | 13.42 | 0.60 |
| STROMBOLIAN_101 | 45.14 | 33.52 | 0.486 | - | 17.59 | 1.41 | 0.0972 | 98.3935 | 86.83 | 12.60 | 0.57 |
| STROMBOLIAN_102 | 45.38 | 33.63 | 0.5175 | - | 17.29 | 1.44 | 0.0889 | 98.5559 | 86.44 | 13.03 | 0.53 |
| STROMBOLIAN_103 | 46.98 | 32.25 | 0.5269 | 0.0302 | 16.01 | 2.31 | 0.1538 | 98.4822 | 78.58 | 20.52 | 0.90 |
| STROMBOLIAN_104 | 48.46 | 31.2 | 0.5607 | 0.0325 | 14.72 | 2.89 | 0.2232 | 98.1707 | 72.82 | 25.87 | 1.31 |
| STROMBOLIAN_105 | 49.46 | 30.43 | 0.5756 | 0.0501 | 13.84 | 3.42 | 0.2953 | 98.3731 | 67.91 | 30.37 | 1.73 |
| STROMBOLIAN_106 | 47.55 | 31.6 | 0.5237 | 0.0387 | 15.24 | 2.64 | 0.1954 | 98.0558 | 75.26 | 23.59 | 1.15 |
| STROMBOLIAN_107 | 48.27 | 31.28 | 0.5195 | 0.0242 | 14.66 | 2.9 | 0.2316 | 98.1296 | 72.63 | 26.00 | 1.37 |
| STROMBOLIAN_108 | 47.68 | 31.87 | 0.4713 | 0.0196 | 15.46 | 2.49 | 0.1769 | 98.4874 | 76.62 | 22.33 | 1.04 |
| STROMBOLIAN_109 | 51.39 | 29.04 | 0.4978 | 0.0429 | 12.33 | 4.06 | 0.441 | 98.0524 | 61.03 | 36.37 | 2.60 |
| STROMBOLIAN_110 | 51.23 | 29 | 0.5313 | 0.0771 | 12.2 | 4.27 | 0.4791 | 98.0911 | 59.52 | 37.70 | 2.78 |
| STROMBOLIAN_111 | 52.1 | 29.18 | 0.4978 | 0.0346 | 12.23 | 4.26 | 0.4468 | 98.9545 | 59.74 | 37.66 | 2.60 |
| STROMBOLIAN_112 | 49.55 | 31.16 | 0.4887 | 0.0237 | 14.64 | 3.08 | 0.2498 | 99.4409 | 71.38 | 27.17 | 1.45 |
| STROMBOLIAN_113 | 47.51 | 32.32 | 0.5267 | - | 16.07 | 2.3 | 0.171 | 99.1498 | 78.64 | 20.37 | 1.00 |
| STROMBOLIAN_114 | 46.28 | 32.96 | 0.4798 | 0.0074 | 16.95 | 1.75 | 0.1266 | 98.7268 | 83.63 | 15.63 | 0.74 |
| STROMBOLIAN_115 | 47.36 | 32.42 | 0.5067 | - | 16.08 | 2.19 | 0.1808 | 99.0464 | 79.37 | 19.56 | 1.06 |
| STROMBOLIAN_116 | 46.96 | 32.72 | 0.4869 | 0.0108 | 16.34 | 2 | 0.1427 | 98.8799 | 81.18 | 17.98 | 0.84 |
| STROMBOLIAN_117 | 47.61 | 32.7 | 0.5064 | - | 16.38 | 2.12 | 0.1492 | 99.6662 | 80.32 | 18.81 | 0.87 |
| STROMBOLIAN_118 | 47.28 | 32.9 | 0.5122 | 0.0109 | 16.26 | 2.09 | 0.1552 | 99.46 | 80.39 | 18.70 | 0.91 |
| STROMBOLIAN_119 | 47.05 | 32.82 | 0.4959 | 0.0015 | 16.47 | 1.88 | 0.1319 | 98.946 | 82.23 | 16.99 | 0.78 |
| STROMBOLIAN_120 | 45.81 | 32.93 | 0.529 | 0.0063 | 16.73 | 1.77 | 0.1274 | 98.0968 | 83.30 | 15.95 | 0.76 |
| STROMBOLIAN_121 | 51.9 | 28.74 | 0.5079 | 0.0333 | 11.84 | 4.54 | 0.513 | 98.3773 | 57.29 | 39.75 | 2.96 |
| STROMBOLIAN_122 | 52.14 | 28.82 | 0.5266 | 0.0551 | 12.15 | 4.3 | 0.5048 | 98.7943 | 59.17 | 37.90 | 2.93 |
| STROMBOLIAN_123 | 53.22 | 28.05 | 0.5129 | 0.04 | 10.91 | 4.78 | 0.6154 | 98.4226 | 53.76 | 42.63 | 3.61 |
| STROMBOLIAN_124 | 50.33 | 30.1 | 0.5258 | 0.0077 | 13.67 | 3.55 | 0.3622 | 98.736 | 66.60 | 31.30 | 2.10 |
| STROMBOLIAN_125 | 51.7 | 29.26 | 0.4821 | 0.0404 | 12.37 | 4.08 | 0.4492 | 98.6713 | 60.97 | 36.39 | 2.64 |
| STROMBOLIAN_126 | 51.47 | 29.08 | 0.5621 | 0.0499 | 12.12 | 4.16 | 0.4771 | 98.2896 | 59.95 | 37.24 | 2.81 |
| STROMBOLIAN_127 | 53.02 | 28.11 | 0.4983 | 0.0274 | 10.97 | 4.96 | 0.6192 | 98.499 | 53.04 | 43.40 | 3.56 |
| STROMBOLIAN_128 | 51.48 | 29.18 | 0.5691 | 0.0479 | 12.22 | 4.23 | 0.4485 | 98.4928 | 59.88 | 37.51 | 2.62 |
| STROMBOLIAN_129 | 51.03 | 29.16 | 0.5339 | 0.0365 | 12.43 | 4.16 | 0.4547 | 98.0496 | 60.64 | 36.72 | 2.64 |
| STROMBOLIAN_130 | 51.35 | 29.03 | 0.6533 | 0.0423 | 12.25 | 4.13 | 0.4763 | 98.248 | 60.37 | 36.83 | 2.79 |
| STROMBOLIAN_131 | 47.17 | 32.68 | 0.4036 | - | 16.07 | 2.11 | 0.1633 | 98.8763 | 80.02 | 19.01 | 0.97 |
| STROMBOLIAN_132 | 46.86 | 32.56 | 0.3837 | - | 16.16 | 2.28 | 0.1751 | 98.5791 | 78.85 | 20.13 | 1.02 |
| STROMBOLIAN_133 | 46.93 | 32.72 | 0.3589 | - | 16.17 | 2.14 | 0.1715 | 98.7464 | 79.86 | 19.13 | 1.01 |
| STROMBOLIAN_134 | 47.25 | 32.65 | 0.3119 | - | 16.26 | 2.23 | 0.1522 | 99.1843 | 79.41 | 19.71 | 0.88 |
| STROMBOLIAN_135 | 47.75 | 32.87 | 0.3482 | - | 16.18 | 2.2 | 0.143 | 99.7485 | 79.58 | 19.58 | 0.84 |
| analysis | SiO2 | Al2O3 | FeO | MgO | CaO | Na2O | K2O | Total | An | Ab | Or |
| STROMBOLIAN_136 | 48.85 | 31.54 | 0.5175 | 0.0334 | 15.02 | 2.9 | 0.2737 | 99.403 | 72.93 | 25.48 | 1.58 |
| STROMBOLIAN_137 | 53.13 | 28.12 | 0.4158 | 0.0045 | 10.92 | 4.86 | 0.6108 | 98.3291 | 53.42 | 43.02 | 3.56 |
| STROMBOLIAN_138 | 46.82 | 32.65 | 0.5253 | - | 16.44 | 1.97 | 0.1309 | 98.7937 | 81.54 | 17.68 | 0.77 |
| STROMBOLIAN_139 | 46.4 | 33.21 | 0.5686 | - | 17.25 | 1.71 | 0.1045 | 99.4677 | 84.27 | 15.12 | 0.61 |
| STROMBOLIAN_140 | 45.69 | 33.83 | 0.5376 | - | 17.68 | 1.43 | 0.0739 | 99.4566 | 86.86 | 12.71 | 0.43 |
| STROMBOLIAN_141 | 47.14 | 32.78 | 0.604 | 0.0071 | 16.52 | 1.99 | 0.144 | 99.4564 | 81.41 | 17.75 | 0.84 |
| STROMBOLIAN_142 | 52.6 | 28.18 | 0.6315 | 0.061 | 11.28 | 4.68 | 0.5647 | 98.2771 | 55.24 | 41.47 | 3.29 |
| STROMBOLIAN_143 | 45.57 | 33.31 | 0.5101 | - | 17.09 | 1.69 | 0.1016 | 98.3904 | 84.32 | 15.09 | 0.60 |
| STROMBOLIAN_144 | 48.41 | 31.09 | 0.6273 | 0.0558 | 14.52 | 2.89 | 0.29 | 98.0844 | 72.26 | 26.03 | 1.72 |
| STROMBOLIAN_145 | 48.6 | 31.09 | 0.5861 | 0.0455 | 14.83 | 2.85 | 0.2612 | 98.5141 | 73.06 | 25.41 | 1.53 |
| STROMBOLIAN_146 | 51.92 | 28.88 | 0.6001 | 0.0695 | 11.82 | 4.38 | 0.5046 | 98.4512 | 58.09 | 38.95 | 2.95 |
| STROMBOLIAN_147 | 47.32 | 32.34 | 0.4569 | - | 16.22 | 2.15 | 0.1505 | 98.6375 | 79.94 | 19.18 | 0.88 |
| STROMBOLIAN_148 | 46.31 | 32.82 | 0.5158 | - | 16.76 | 1.81 | 0.1212 | 98.3979 | 83.05 | 16.23 | 0.72 |
| STROMBOLIAN_149 | 47.47 | 32.29 | 0.462 | - | 16.04 | 2.23 | 0.1565 | 98.7543 | 79.16 | 19.92 | 0.92 |
| STROMBOLIAN_150 | 46.55 | 32.94 | 0.4588 | - | 16.97 | 1.74 | 0.1291 | 98.8214 | 83.71 | 15.53 | 0.76 |
| STROMBOLIAN_151 | 47.26 | 32.63 | 0.4736 | - | 16.28 | 2.06 | 0.1357 | 98.8871 | 80.72 | 18.48 | 0.80 |
| STROMBOLIAN_152 | 46.76 | 32.97 | 0.4445 | - | 16.94 | 1.74 | 0.1199 | 98.9983 | 83.73 | 15.56 | 0.71 |
| STROMBOLIAN_153 | 48.16 | 31.94 | 0.6249 | - | 15.66 | 2.44 | 0.2109 | 99.0876 | 77.04 | 21.72 | 1.24 |
| STROMBOLIAN_154 | 48.32 | 32.26 | 0.4191 | - | 15.69 | 2.31 | 0.1943 | 99.1987 | 78.05 | 20.80 | 1.15 |
| STROMBOLIAN_155 | 48 | 32.23 | 0.4482 | - | 16.03 | 2.25 | 0.1601 | 99.1909 | 79.00 | 20.06 | 0.94 |
| STROMBOLIAN_156 | 48.21 | 31.49 | 0.826 | 0.0058 | 15.44 | 2.51 | 0.3902 | 98.9365 | 75.51 | 22.21 | 2.27 |
| STROMBOLIAN_157 | 49.15 | 31.37 | 0.4701 | - | 15.14 | 2.85 | 0.2476 | 99.3063 | 73.52 | 25.05 | 1.43 |
| STROMBOLIAN_158 | 46.12 | 29.72 | 0.4061 | - | 14.44 | 2.43 | 0.2258 | 93.3803 | 75.58 | 23.02 | 1.41 |
| STROMBOLIAN_159 | 47.68 | 32.01 | 0.4461 | - | 15.73 | 2.4 | 0.2082 | 98.5134 | 77.41 | 21.37 | 1.22 |
| STROMBOLIAN_160 | 53.13 | 30.23 | 0.3921 | - | 12.82 | 4.22 | 0.4596 | 101.3728 | 61.04 | 36.36 | 2.61 |
| STROMBOLIAN_161 | 49.88 | 30.88 | 0.4265 | - | 14.43 | 3 | 0.2577 | 98.8751 | 71.56 | 26.92 | 1.52 |
| STROMBOLIAN_162 | 48.55 | 31.05 | 0.9751 | 0.0136 | 14.98 | 2.61 | 0.4112 | 98.5984 | 74.19 | 23.39 | 2.42 |
| STROMBOLIAN_163 | 48.81 | 31.47 | 0.4868 | - | 15.38 | 2.55 | 0.2098 | 98.9503 | 75.97 | 22.79 | 1.23 |
| STROMBOLIAN_164 | 47.97 | 31.96 | 0.4585 | - | 16.04 | 2.18 | 0.1783 | 98.8349 | 79.42 | 19.53 | 1.05 |
| STROMBOLIAN_165 | 46.78 | 33.05 | 0.4871 | - | 17.03 | 1.73 | 0.1322 | 99.2508 | 83.82 | 15.41 | 0.77 |
| STROMBOLIAN_166 | 52.83 | 28.74 | 0.5926 | - | 12.02 | 4.25 | 0.5152 | 99.021 | 59.14 | 37.84 | 3.02 |
| STROMBOLIAN_167 | 53.16 | 27.63 | 1.0209 | 0.0917 | 11.44 | 4.29 | 0.8525 | 98.5731 | 56.58 | 38.40 | 5.02 |
| STROMBOLIAN_168 | 53.18 | 28.3 | 0.8152 | 0.0224 | 11.71 | 4.21 | 0.6255 | 99.0174 | 58.34 | 37.95 | 3.71 |
| STROMBOLIAN_169 | 51.69 | 28.97 | 0.939 | 0.0307 | 12.72 | 3.82 | 0.4619 | 98.7337 | 63.02 | 34.25 | 2.72 |
| STROMBOLIAN_170 | 46.52 | 33.08 | 0.628 | - | 17.31 | 1.51 | 0.0905 | 99.1413 | 85.90 | 13.56 | 0.53 |
| STROMBOLIAN_171 | 53.69 | 27.98 | 0.733 | 0.0326 | 11.31 | 4.5 | 0.566 | 98.8714 | 56.19 | 40.46 | 3.35 |
| STROMBOLIAN_172 | 46.93 | 32.47 | 0.7204 | 0.0046 | 16.74 | 1.87 | 0.1126 | 98.8625 | 82.63 | 16.70 | 0.66 |
| STROMBOLIAN_173 | 49.25 | 30.8 | 0.709 | 0.0013 | 14.68 | 2.75 | 0.2074 | 98.4479 | 73.76 | 25.00 | 1.24 |
| STROMBOLIAN_174 | 47.93 | 31.89 | 0.7307 | 0.0111 | 15.9 | 2.18 | 0.177 | 98.8798 | 79.28 | 19.67 | 1.05 |
| STROMBOLIAN_175 | 52.47 | 28.78 | 0.8335 | 0.0405 | 12.3 | 4.06 | 0.4426 | 98.9854 | 60.97 | 36.42 | 2.61 |
| STROMBOLIAN_176 | 49.96 | 30.72 | 0.94 | 0.0047 | 14.31 | 3.05 | 0.3162 | 99.3372 | 70.82 | 27.32 | 1.86 |
| STROMBOLIAN_177 | 53.08 | 28.03 | 0.9434 | 0.0389 | 11.38 | 4.33 | 0.6118 | 98.4705 | 57.06 | 39.29 | 3.65 |
| STROMBOLIAN_178 | 48.34 | 32.39 | 1.1799 | 0.0081 | 15.46 | 2.31 | 0.2201 | 99.9373 | 77.68 | 21.00 | 1.32 |
| STROMBOLIAN_179 | 49.44 | 31.36 | 1.0588 | 0.023 | 14.56 | 2.95 | 0.2914 | 99.7208 | 71.92 | 26.37 | 1.71 |
| STROMBOLIAN_180 | 47.49 | 32.52 | 1.1697 | - | 15.82 | 2.16 | 0.1796 | 99.3777 | 79.33 | 19.60 | 1.07 |
| analysis | SiO2 | Al2O3 | FeO | MgO | CaO | Na2O | K2O | Total | An | Ab | Or |
| STROMBOLIAN_181 | 48.5 | 31.94 | 1.1035 | 0.0172 | 15.22 | 2.55 | 0.2117 | 99.5424 | 75.77 | 22.97 | 1.25 |
| STROMBOLIAN_182 | 47.52 | 32.73 | 1.1539 | 0.0055 | 15.93 | 2.16 | 0.1662 | 99.7136 | 79.50 | 19.51 | 0.99 |
| STROMBOLIAN_183 | 47.89 | 32.53 | 1.1277 | 0.0007 | 15.89 | 2.24 | 0.1871 | 99.8656 | 78.79 | 20.10 | 1.10 |
| STROMBOLIAN_184 | 46.75 | 33.19 | 1.2138 | 0.0047 | 16.54 | 1.79 | 0.1444 | 99.6908 | 82.90 | 16.24 | 0.86 |
| STROMBOLIAN_185 | 47.21 | 33.09 | 1.2107 | 0.0005 | 16.12 | 2 | 0.1607 | 99.8191 | 80.88 | 18.16 | 0.96 |
| STROMBOLIAN_186 | 47.8 | 32.53 | 1.1918 | 0.0082 | 15.75 | 2.25 | 0.1912 | 99.7466 | 78.56 | 20.31 | 1.14 |
| STROMBOLIAN_187 | 47.6 | 32.64 | 1.1807 | 0.0044 | 15.86 | 2.19 | 0.1728 | 99.6804 | 79.19 | 19.79 | 1.03 |
| STROMBOLIAN_188 | 46.99 | 32.65 | 1.1527 | - | 16.15 | 1.94 | 0.1513 | 99.0979 | 81.40 | 17.69 | 0.91 |
| STROMBOLIAN_189 | 47.36 | 33 | 1.1692 | - | 16.1 | 1.96 | 0.159 | 99.786 | 81.16 | 17.88 | 0.95 |
| STROMBOLIAN_190 | 47.99 | 32.56 | 1.1035 | - | 15.83 | 2.16 | 0.1746 | 99.8275 | 79.36 | 19.60 | 1.04 |
| STROMBOLIAN_191 | 45.76 | 32.9 | 1.3 | - | 16.61 | 1.62 | 0.1439 | 98.3783 | 84.26 | 14.87 | 0.87 |
| STROMBOLIAN_192 | 52.66 | 28.95 | 1.31 | 0.0237 | 11.76 | 4.22 | 0.5491 | 99.5489 | 58.65 | 38.09 | 3.26 |
| STROMBOLIAN_193 | 47.24 | 32.84 | 1.1749 | - | 16.22 | 1.93 | 0.1671 | 99.5912 | 81.46 | 17.54 | 1.00 |
| STROMBOLIAN_194 | 47.31 | 31.35 | 2.5 | 0.2786 | 15.25 | 2.2 | 0.5398 | 99.4596 | 76.73 | 20.03 | 3.23 |
| STROMBOLIAN_195 | 47.35 | 32.92 | 1.0918 | - | 16.14 | 2.02 | 0.1546 | 99.6989 | 80.78 | 18.30 | 0.92 |
| STROMBOLIAN_196 | 47.05 | 33.19 | 1.1555 | - | 16.39 | 1.83 | 0.135 | 99.7531 | 82.52 | 16.67 | 0.81 |
| STROMBOLIAN_197 | 53.88 | 28.25 | 1.1926 | 0.0446 | 11 | 4.8 | 0.588 | 99.7553 | 53.96 | 42.61 | 3.43 |
| STROMBOLIAN_198 | 52.99 | 29.14 | 1.1802 | 0.04 | 11.99 | 4.32 | 0.4933 | 100.2157 | 58.79 | 38.33 | 2.88 |
| STROMBOLIAN_199 | 53.39 | 28.47 | 1.2339 | 0.0493 | 11.33 | 4.65 | 0.5726 | 99.782 | 55.47 | 41.20 | 3.34 |
| STROMBOLIAN_200 | 53.03 | 28.88 | 1.1709 | 0.0552 | 11.63 | 4.27 | 0.5495 | 99.6502 | 58.12 | 38.61 | 3.27 |
| STROMBOLIAN_201 | 51.13 | 30.22 | 1.31 | 0.0452 | 12.98 | 3.75 | 0.4123 | 99.8529 | 64.08 | 33.50 | 2.42 |
| STROMBOLIAN_202 | 54.98 | 27.49 | 1.41 | 0.0583 | 9.98 | 5.27 | 0.7654 | 100.0524 | 48.85 | 46.68 | 4.46 |

| analysis | SiO2 | Al2O3 | FeO | MnO | MgO | CaO | Na2O | K2O | Total | An | Ab | Or |
| --- | --- | --- | --- | --- | --- | --- | --- | --- | --- | --- | --- | --- |
| Lava_1 | 45.59 | 33.23 | 0.5514 | 0.0106 | - | 16.9 | 1.7 | 0.1046 | 98.2512 | 84.08 | 15.30 | 0.62 |
| Lava_2 | 46.4 | 32.5 | 0.6082 | - | 0.0143 | 16.04 | 2.1 | 0.1577 | 98.0794 | 80.09 | 18.97 | 0.94 |
| Lava_3 | 53.57 | 27.72 | 0.4974 | 0.0316 | 0.0537 | 10.73 | 4.88 | 0.6484 | 98.4192 | 52.77 | 43.43 | 3.80 |
| Lava_4 | 52.63 | 28.13 | 0.5199 | 0.0288 | 0.0457 | 11.25 | 4.73 | 0.5648 | 98.2109 | 54.93 | 41.79 | 3.28 |
| Lava_5 | 52.46 | 28.34 | 0.5217 | - | 0.0706 | 11.42 | 4.5 | 0.5409 | 98.1703 | 56.51 | 40.30 | 3.19 |
| Lava_6 | 51.37 | 29.14 | 0.534 | 0.0028 | 0.0396 | 12.26 | 4.21 | 0.4547 | 98.3625 | 60.04 | 37.31 | 2.65 |
| Lava_7 | 52.36 | 28.48 | 0.4696 | 0.0021 | 0.0254 | 11.68 | 4.37 | 0.5132 | 98.1796 | 57.82 | 39.15 | 3.03 |
| Lava_8 | 51.29 | 28.84 | 0.6148 | 0.0007 | 0.0567 | 12.26 | 4.21 | 0.5008 | 98.079 | 59.88 | 37.21 | 2.91 |
| Lava_9 | 46.57 | 32.56 | 0.5339 | 0.0105 | 0.0108 | 16.53 | 1.9 | 0.1639 | 98.5022 | 81.98 | 17.05 | 0.97 |
| Lava_10 | 47.01 | 33.28 | 0.5155 | - | 0.0102 | 16.86 | 1.77 | 0.1358 | 99.9242 | 83.36 | 15.84 | 0.80 |
| Lava_11 | 50.9 | 29.08 | 1.0436 | 0.033 | 0.1775 | 12.72 | 3.92 | 0.5854 | 98.7773 | 62.02 | 34.59 | 3.40 |
| Lava_12 | 52.16 | 28.97 | 0.6583 | 0.0014 | 0.0573 | 12.33 | 4.12 | 0.4787 | 99.1064 | 60.57 | 36.63 | 2.80 |
| Lava_13 | 51.33 | 29.84 | 0.5241 | 0.0014 | 0.0307 | 13.31 | 3.74 | 0.2975 | 99.3568 | 65.14 | 33.12 | 1.73 |
| Lava_14 | 51.28 | 30.24 | 0.5434 | 0.0063 | 0.0593 | 13.55 | 3.73 | 0.2817 | 99.9152 | 65.66 | 32.71 | 1.63 |
| Lava_15 | 50.92 | 28.15 | 1.52 | 0.0309 | 0.5524 | 13.12 | 3.68 | 0.641 | 98.8873 | 63.87 | 32.42 | 3.72 |
| Lava_16 | 49.99 | 30.86 | 0.5566 | 0.0056 | - | 14.36 | 3.21 | 0.2187 | 99.4176 | 70.29 | 28.43 | 1.27 |
| Lava_17 | 51.24 | 30.29 | 0.5637 | - | 0.0425 | 13.65 | 3.7 | 0.2917 | 100.0998 | 65.96 | 32.36 | 1.68 |
| Lava_18 | 50.99 | 30.28 | 0.5247 | - | 0.0372 | 13.71 | 3.56 | 0.2813 | 99.7244 | 66.92 | 31.45 | 1.63 |
| Lava_19 | 52.07 | 29.62 | 0.5194 | 0.0091 | 0.0287 | 12.85 | 4.09 | 0.3465 | 99.7285 | 62.19 | 35.82 | 2.00 |
| Lava_20 | 51.28 | 30.38 | 0.503 | - | 0.063 | 13.74 | 3.7 | 0.3036 | 100.2302 | 66.07 | 32.19 | 1.74 |
| Lava_21 | 51.3 | 30.15 | 0.5025 | 0.0112 | 0.0536 | 13.67 | 3.55 | 0.3168 | 99.7582 | 66.78 | 31.38 | 1.84 |
| Lava_22 | 51.96 | 29.74 | 0.5635 | 0.019 | 0.0693 | 13.05 | 3.91 | 0.3488 | 99.919 | 63.53 | 34.45 | 2.02 |
| Lava_23 | 52.93 | 29.07 | 0.567 | 0.0309 | 0.0876 | 12.53 | 4.18 | 0.3998 | 100.0685 | 60.91 | 36.77 | 2.31 |
| Lava_24 | 53.05 | 29.23 | 0.5783 | - | 0.0609 | 12.43 | 4.2 | 0.3769 | 100.1782 | 60.70 | 37.11 | 2.19 |
| Lava_25 | 52.77 | 29.12 | 0.5599 | - | 0.0623 | 12.48 | 4.27 | 0.3652 | 99.9317 | 60.46 | 37.43 | 2.11 |
| Lava_26 | 52.36 | 29.65 | 0.5604 | - | 0.0757 | 12.8 | 3.96 | 0.355 | 100.1205 | 62.78 | 35.15 | 2.07 |
| Lava_27 | 49.12 | 31.92 | 0.5256 | 0.033 | 0.0345 | 15.5 | 2.74 | 0.1888 | 100.1606 | 74.94 | 23.97 | 1.09 |
| Lava_28 | 48.99 | 31.42 | 0.5707 | - | 0.0395 | 15.16 | 2.69 | 0.1961 | 99.2421 | 74.82 | 24.03 | 1.15 |
| Lava_29 | 48.07 | 32.31 | 0.6225 | 0.0203 | 0.0334 | 16.31 | 2.16 | 0.1737 | 99.9558 | 79.85 | 19.14 | 1.01 |
| Lava_30 | 54.68 | 27.71 | 0.6362 | 0.0162 | 0.1178 | 10.86 | 4.91 | 0.6826 | 99.7788 | 52.83 | 43.22 | 3.95 |
| Lava_31 | 48.16 | 31.39 | 0.5212 | - | 0.0015 | 15.07 | 2.64 | 0.21 | 98.159 | 74.98 | 23.77 | 1.24 |
| Lava_32 | 49.19 | 31.04 | 0.5191 | 0.0056 | 0.0259 | 14.55 | 2.98 | 0.2902 | 98.7989 | 71.72 | 26.58 | 1.70 |
| Lava_33 | 51.73 | 28.91 | 0.5104 | 0.0211 | 0.0234 | 12.15 | 4.22 | 0.4854 | 98.2827 | 59.66 | 37.50 | 2.84 |
| Lava_34 | 51.36 | 29.42 | 0.5164 | - | 0.0279 | 12.57 | 4.04 | 0.4269 | 98.6101 | 61.65 | 35.86 | 2.49 |
| Lava_35 | 51.22 | 29.4 | 0.5156 | - | 0.0257 | 12.71 | 4.08 | 0.4474 | 98.6217 | 61.62 | 35.80 | 2.58 |
| Lava_36 | 51.53 | 29.17 | 0.4774 | 0.0197 | 0.0497 | 12.49 | 4.09 | 0.4506 | 98.3431 | 61.14 | 36.23 | 2.63 |
| Lava_37 | 52.98 | 28.16 | 0.4828 | 0.0175 | 0.0578 | 11.38 | 4.76 | 0.5848 | 98.6556 | 55.00 | 41.63 | 3.37 |
| Lava_38 | 52.28 | 28.89 | 0.4857 | 0.0162 | 0.0453 | 11.97 | 4.4 | 0.4704 | 98.8242 | 58.41 | 38.85 | 2.73 |
| Lava_39 | 52.25 | 28.89 | 0.5214 | 0.0267 | 0.0566 | 12.23 | 4.24 | 0.4589 | 98.8861 | 59.81 | 37.52 | 2.67 |
| Lava_40 | 52.26 | 29.02 | 0.5258 | - | 0.0188 | 12.17 | 4.36 | 0.4758 | 99.1367 | 59.00 | 38.25 | 2.75 |
| Lava_41 | 51.88 | 28.86 | 0.5567 | - | 0.0786 | 12.16 | 4.18 | 0.4631 | 98.5193 | 59.97 | 37.31 | 2.72 |
| Lava_42 | 51.26 | 29.3 | 0.5605 | 0.0162 | 0.0403 | 12.71 | 3.92 | 0.4619 | 98.4431 | 62.45 | 34.85 | 2.70 |
| Lava_43 | 52.14 | 28.41 | 0.7713 | 0.0147 | 0.1201 | 11.61 | 4.41 | 0.6002 | 98.419 | 57.18 | 39.30 | 3.52 |
| Lava_44 | 50.33 | 29.85 | 0.6663 | - | 0.0685 | 13.29 | 3.55 | 0.4168 | 98.5714 | 65.76 | 31.79 | 2.46 |
| Lava_45 | 45.83 | 33.76 | 0.5183 | 0.014 | - | 17.67 | 1.44 | 0.0902 | 99.5475 | 86.69 | 12.78 | 0.53 |
| analysis | SiO2 | Al2O3 | FeO | MnO | MgO | CaO | Na2O | K2O | Total | An | Ab | Or |
| Lava_46 | 45.35 | 33.58 | 0.4801 | - | 0.0192 | 17.88 | 1.333 | 0.0569 | 98.8746 | 87.82 | 11.85 | 0.33 |
| Lava_47 | 44.91 | 33.88 | 0.4916 | 0.0168 | 0.0027 | 17.92 | 1.1704 | 0.0719 | 98.6121 | 89.05 | 10.52 | 0.43 |
| Lava_48 | 45.87 | 33.24 | 0.5894 | - | - | 17.15 | 1.61 | 0.1345 | 98.7862 | 84.80 | 14.41 | 0.79 |
| Lava_49 | 52.94 | 28.52 | 0.5015 | 0.0014 | 0.0504 | 11.55 | 4.64 | 0.5438 | 99.021 | 56.08 | 40.77 | 3.14 |
| Lava_50 | 45.49 | 33.81 | 0.4726 | 0.0056 | 0.007 | 18.08 | 1.244 | 0.0539 | 99.452 | 88.65 | 11.04 | 0.31 |
| Lava_51 | 45.81 | 33.81 | 0.4521 | 0.0393 | - | 17.97 | 1.37 | 0.0511 | 99.8053 | 87.62 | 12.09 | 0.30 |
| Lava_52 | 45.82 | 33.94 | 0.4799 | 0.0218 | - | 17.92 | 1.3249 | 0.0533 | 99.7324 | 87.92 | 11.76 | 0.31 |
| Lava_53 | 45.54 | 33.85 | 0.4915 | 0.0126 | 0.0028 | 17.78 | 1.3148 | 0.0735 | 99.2626 | 87.82 | 11.75 | 0.43 |
| Lava_54 | 47.01 | 33.02 | 0.4911 | - | 0.0196 | 16.77 | 1.85 | 0.1121 | 99.4527 | 82.81 | 16.53 | 0.66 |
| Lava_55 | 45.64 | 33.78 | 0.4812 | - | 0.0094 | 17.88 | 1.3107 | 0.0529 | 99.3271 | 88.01 | 11.68 | 0.31 |
| Lava_56 | 47.14 | 32.79 | 0.5173 | 0.0197 | 0.0177 | 16.75 | 1.9 | 0.0955 | 99.4985 | 82.50 | 16.94 | 0.56 |
| Lava_57 | 46.7 | 33.26 | 0.4795 | 0.0351 | - | 17.04 | 1.68 | 0.1076 | 99.565 | 84.32 | 15.04 | 0.63 |
| Lava_58 | 46.13 | 33.67 | 0.4586 | 0.04 | 0.0059 | 17.46 | 1.61 | 0.0698 | 99.6898 | 85.35 | 14.24 | 0.41 |
| Lava_59 | 45.74 | 34.18 | 0.4637 | - | 0.0014 | 18.24 | 1.2236 | 0.0512 | 100.132 | 88.91 | 10.79 | 0.30 |
| Lava_60 | 45.45 | 34.06 | 0.5155 | 0.0379 | 0.0057 | 18.02 | 1.186 | 0.0655 | 99.5331 | 89.01 | 10.60 | 0.39 |
| Lava_61 | 46.82 | 33.3 | 0.4999 | - | 0.0048 | 16.85 | 1.79 | 0.1049 | 99.6057 | 83.36 | 16.02 | 0.62 |
| Lava_62 | 46.66 | 33.08 | 0.4848 | - | 0.0194 | 16.99 | 1.76 | 0.0874 | 99.3535 | 83.78 | 15.71 | 0.51 |
| Lava_63 | 46.61 | 33.18 | 0.5007 | - | 0.0119 | 17.25 | 1.71 | 0.0865 | 99.6122 | 84.36 | 15.13 | 0.50 |
| Lava_64 | 46.02 | 33.56 | 0.4868 | 0.0042 | 0.0191 | 17.6 | 1.48 | 0.0833 | 99.4885 | 86.37 | 13.14 | 0.49 |
| Lava_65 | 45.48 | 34.05 | 0.5127 | 0.0007 | - | 18.05 | 1.1809 | 0.0636 | 99.5125 | 89.08 | 10.55 | 0.37 |
| Lava_66 | 45.55 | 33.59 | 0.5253 | - | - | 17.86 | 1.1585 | 0.0685 | 98.9008 | 89.13 | 10.46 | 0.41 |
| Lava_67 | 46.03 | 33.57 | 0.5337 | 0.0155 | 0.0035 | 17.4 | 1.42 | 0.1129 | 99.3062 | 86.55 | 12.78 | 0.67 |
| Lava_68 | 51.76 | 29.14 | 0.538 | - | 0.0356 | 12.57 | 3.95 | 0.4187 | 98.7429 | 62.18 | 35.36 | 2.47 |
| Lava_69 | 52.2 | 29.29 | 0.5819 | 0.0155 | 0.0392 | 12.48 | 4.09 | 0.4384 | 99.4376 | 61.17 | 36.28 | 2.56 |
| Lava_70 | 52.61 | 28.62 | 0.5757 | 0.0056 | 0.0679 | 12.11 | 4.23 | 0.5027 | 98.8822 | 59.47 | 37.59 | 2.94 |
| Lava_71 | 46.15 | 33.14 | 0.4818 | 0.0204 | - | 17.16 | 1.63 | 0.0928 | 98.8932 | 84.87 | 14.59 | 0.55 |
| Lava_72 | 51.82 | 29.04 | 0.4794 | 0.0239 | 0.0619 | 12.32 | 4.14 | 0.4465 | 98.6576 | 60.56 | 36.83 | 2.61 |
| Lava_73 | 48 | 31.91 | 0.5582 | 0.0337 | - | 15.69 | 2.36 | 0.2086 | 98.922 | 77.64 | 21.13 | 1.23 |
| Lava_74 | 52.78 | 28.43 | 0.5319 | 0.0436 | 0.0582 | 11.85 | 4.37 | 0.4618 | 98.757 | 58.35 | 38.94 | 2.71 |
| Lava_75 | 55.28 | 27.27 | 0.7728 | 0.0224 | 0.082 | 10.31 | 5.24 | 0.7806 | 100.0891 | 49.75 | 45.76 | 4.49 |
| Lava_76 | 53.41 | 28.4 | 0.7671 | 0.0168 | 0.0819 | 11.89 | 4.43 | 0.572 | 99.8048 | 57.75 | 38.94 | 3.31 |
| Lava_77 | 57.21 | 26.22 | 0.6092 | 0.0126 | 0.0379 | 9.02 | 5.72 | 1.0004 | 100.0892 | 43.87 | 50.34 | 5.79 |
| Lava_78 | 52.56 | 26.3 | 2.57 | 0.0322 | 0.2129 | 10.52 | 4.58 | 1.1533 | 98.1489 | 52.13 | 41.07 | 6.80 |
| Lava_79 | 46.86 | 33.43 | 0.5434 | - | 0.0048 | 17.32 | 1.66 | 0.1174 | 100.179 | 84.64 | 14.68 | 0.68 |
| Lava_80 | 47.52 | 32.84 | 0.5931 | - | 0.0154 | 16.78 | 1.95 | 0.1231 | 100.01 | 82.03 | 17.25 | 0.72 |
| Lava_81 | 47.46 | 32.93 | 0.5817 | - | 0.0292 | 16.77 | 1.82 | 0.1272 | 99.9336 | 82.96 | 16.29 | 0.75 |
| Lava_82 | 47.63 | 32.36 | 0.7065 | 0.0224 | 0.0829 | 16.6 | 1.93 | 0.1926 | 99.725 | 81.69 | 17.19 | 1.13 |
| Lava_83 | 47.29 | 33.31 | 0.5582 | 0.0203 | - | 16.72 | 1.78 | 0.1184 | 100.0106 | 83.26 | 16.04 | 0.70 |
| Lava_84 | 47.04 | 32.88 | 0.604 | 0.0063 | 0.0097 | 17.05 | 1.72 | 0.1307 | 99.5665 | 83.92 | 15.32 | 0.77 |
| Lava_85 | 48.35 | 32.27 | 0.561 | - | 0.0248 | 16.02 | 2.15 | 0.1719 | 99.7419 | 79.64 | 19.34 | 1.02 |
| Lava_86 | 52.95 | 29.19 | 0.5414 | 0.014 | 0.0357 | 12.33 | 4.08 | 0.4277 | 99.7342 | 60.97 | 36.51 | 2.52 |
| Lava_87 | 53.14 | 29.11 | 0.5502 | 0.0042 | 0.0534 | 12.43 | 4.25 | 0.4543 | 100.1909 | 60.16 | 37.22 | 2.62 |
| Lava_88 | 53.68 | 28.64 | 0.5259 | 0.0021 | 0.0678 | 11.98 | 4.43 | 0.4874 | 100.0349 | 58.22 | 38.96 | 2.82 |
| Lava_89 | 52.73 | 29.16 | 0.4872 | - | 0.044 | 12.58 | 4.02 | 0.4436 | 99.7092 | 61.72 | 35.69 | 2.59 |
| Lava_90 | 53.18 | 28.99 | 0.5071 | 0.0028 | 0.0506 | 12.19 | 4.41 | 0.5088 | 100.0656 | 58.67 | 38.41 | 2.92 |
| analysis | SiO2 | Al2O3 | FeO | MnO | MgO | CaO | Na2O | K2O | Total | An | Ab | Or |
| Lava_91 | 52.37 | 29.48 | 0.5749 | 0.028 | 0.0558 | 12.79 | 3.91 | 0.3994 | 99.9319 | 62.88 | 34.78 | 2.34 |
| Lava_92 | 53.24 | 28.86 | 0.56 | 0.0204 | 0.0875 | 12.11 | 4.27 | 0.4871 | 99.7936 | 59.31 | 37.85 | 2.84 |
| Lava_93 | 51.59 | 29.61 | 0.5486 | 0.0147 | 0.0317 | 13.3 | 3.75 | 0.3923 | 99.4223 | 64.71 | 33.02 | 2.27 |
| Lava_94 | 52.96 | 28.97 | 0.5577 | 0.0232 | 0.0534 | 12.15 | 4.2 | 0.4628 | 99.6297 | 59.85 | 37.44 | 2.71 |
| Lava_95 | 52.5 | 29.34 | 0.6251 | - | 0.0278 | 12.47 | 4.15 | 0.4559 | 99.7706 | 60.76 | 36.59 | 2.64 |

Table S1. Plagioclase Elemental Data.

| Analysis | SiO2 | TiO2 | Al2O3 | Cr2O3 | FeO | MnO | MgO | CaO | Na2O | NiO | Total | Fo |
| --- | --- | --- | --- | --- | --- | --- | --- | --- | --- | --- | --- | --- |
| Tephra_1 | 38.01 | 0.04 | 0.03 | 0.00 | 23.45 | 0.58 | 36.31 | 0.38 | 0.02 | 0.00 | 98.82 | 0.73 |
| Tephra_2 | 38.09 | 0.02 | 0.02 | 0.02 | 24.83 | 0.56 | 36.18 | 0.34 | 0.00 | 0.00 | 100.07 | 0.72 |
| Tephra_3 | 37.89 | 0.05 | 0.02 | 0.00 | 25.37 | 0.58 | 36.50 | 0.37 | 0.02 | 0.00 | 100.80 | 0.72 |
| Tephra_4 | 37.68 | 0.06 | 0.04 | 0.01 | 25.55 | 0.60 | 35.77 | 0.45 | 0.02 | 0.00 | 100.18 | 0.71 |
| Tephra_5 | 37.43 | 0.03 | 0.04 | 0.02 | 25.40 | 0.56 | 35.98 | 0.38 | 0.01 | 0.00 | 99.86 | 0.72 |
| Tephra_6 | 37.82 | 0.03 | 0.04 | 0.01 | 24.11 | 0.53 | 36.98 | 0.48 | 0.01 | 0.00 | 100.02 | 0.73 |
| Tephra_7 | 38.08 | 0.05 | 0.03 | 0.01 | 23.94 | 0.52 | 36.83 | 0.52 | 0.00 | 0.00 | 99.98 | 0.73 |
| Tephra_8 | 37.83 | 0.04 | 0.01 | 0.00 | 25.30 | 0.60 | 36.36 | 0.40 | 0.02 | 0.00 | 100.56 | 0.72 |
| Tephra_9 | 37.70 | 0.03 | 0.02 | 0.00 | 24.96 | 0.54 | 35.98 | 0.38 | 0.02 | 0.00 | 99.65 | 0.72 |
| Tephra_10 | 38.08 | 0.00 | 0.04 | 0.00 | 25.65 | 0.58 | 36.06 | 0.44 | 0.01 | 0.00 | 100.90 | 0.71 |
| Tephra_11 | 37.64 | 0.02 | 0.02 | 0.02 | 27.22 | 0.63 | 34.25 | 0.41 | 0.00 | 0.00 | 100.22 | 0.69 |
| Tephra_12 | 37.84 | 0.05 | 0.05 | 0.00 | 25.71 | 0.64 | 35.57 | 0.42 | 0.00 | 0.00 | 100.28 | 0.71 |
| Tephra_13 | 37.85 | 0.05 | 0.04 | 0.00 | 24.45 | 0.61 | 36.53 | 0.46 | 0.00 | 0.00 | 100.00 | 0.73 |
| Tephra_14 | 38.07 | 0.05 | 0.03 | 0.00 | 25.35 | 0.58 | 35.26 | 0.42 | 0.03 | 0.00 | 99.80 | 0.71 |
| Tephra_15 | 38.07 | 0.05 | 0.04 | 0.00 | 23.92 | 0.54 | 36.46 | 0.43 | 0.01 | 0.00 | 99.53 | 0.73 |
| Tephra_16 | 37.59 | 0.02 | 0.03 | 0.00 | 26.64 | 0.64 | 34.23 | 0.44 | 0.01 | 0.00 | 99.61 | 0.70 |
| Tephra_17 | 37.53 | 0.02 | 0.04 | 0.00 | 26.71 | 0.64 | 34.21 | 0.46 | 0.03 | 0.00 | 99.65 | 0.70 |
| Tephra_18 | 37.69 | 0.02 | 0.02 | 0.00 | 26.41 | 0.64 | 34.11 | 0.50 | 0.01 | 0.00 | 99.40 | 0.70 |
| Tephra_19 | 38.10 | 0.03 | 0.00 | 0.00 | 24.81 | 0.59 | 36.07 | 0.44 | 0.04 | 0.00 | 100.09 | 0.72 |
| Tephra_20 | 38.23 | 0.03 | 0.02 | 0.02 | 23.44 | 0.50 | 36.97 | 0.28 | 0.03 | 0.00 | 99.52 | 0.74 |
| Tephra_21 | 37.81 | 0.02 | 0.05 | 0.02 | 24.48 | 0.58 | 35.62 | 0.43 | 0.01 | 0.00 | 99.03 | 0.72 |
| Tephra_22 | 37.85 | 0.05 | 0.04 | 0.02 | 24.95 | 0.60 | 35.51 | 0.43 | 0.01 | 0.00 | 99.45 | 0.72 |
| Tephra_23 | 37.12 | 0.04 | 0.03 | 0.02 | 26.70 | 0.65 | 34.64 | 0.44 | 0.01 | 0.01 | 99.67 | 0.70 |
| Tephra_24 | 37.39 | 0.04 | 0.04 | 0.00 | 26.75 | 0.62 | 34.74 | 0.43 | 0.02 | 0.00 | 100.04 | 0.70 |
| Tephra_25 | 37.34 | 0.01 | 0.04 | 0.02 | 26.68 | 0.61 | 34.73 | 0.42 | 0.03 | 0.01 | 99.91 | 0.70 |
| Tephra_26 | 37.41 | 0.04 | 0.03 | 0.02 | 26.61 | 0.59 | 34.59 | 0.42 | 0.03 | 0.00 | 99.74 | 0.70 |
| Tephra_27 | 37.48 | 0.01 | 0.05 | 0.00 | 26.74 | 0.55 | 34.61 | 0.39 | 0.00 | 0.01 | 99.84 | 0.70 |
| Tephra_28 | 37.12 | 0.09 | 0.03 | 0.00 | 26.72 | 0.60 | 34.74 | 0.46 | 0.00 | 0.00 | 99.76 | 0.70 |
| Analysis | SiO2 | TiO2 | Al2O3 | Cr2O3 | FeO | MnO | MgO | CaO | Na2O | NiO | Total | Fo |
| Tephra_29 | 37.45 | 0.03 | 0.04 | 0.00 | 26.86 | 0.58 | 35.15 | 0.37 | 0.01 | 0.01 | 100.48 | 0.70 |
| Tephra_30 | 37.41 | 0.05 | 0.04 | 0.00 | 25.70 | 0.59 | 35.15 | 0.46 | 0.02 | 0.01 | 99.44 | 0.71 |
| Tephra_31 | 37.46 | 0.03 | 0.03 | 0.00 | 26.77 | 0.65 | 34.83 | 0.36 | 0.00 | 0.00 | 100.14 | 0.70 |
| Tephra_32 | 37.57 | 0.00 | 0.01 | 0.00 | 26.97 | 0.59 | 34.80 | 0.39 | 0.02 | 0.02 | 100.38 | 0.70 |
| Tephra_33 | 37.20 | 0.02 | 0.03 | 0.00 | 25.46 | 0.56 | 35.92 | 0.38 | 0.02 | 0.00 | 99.58 | 0.72 |
| Tephra_34 | 37.92 | 0.03 | 0.04 | 0.00 | 23.85 | 0.53 | 37.17 | 0.44 | 0.00 | 0.00 | 99.99 | 0.74 |
| Tephra_35 | 37.68 | 0.03 | 0.02 | 0.01 | 24.81 | 0.54 | 36.49 | 0.43 | 0.01 | 0.04 | 100.05 | 0.72 |
| Tephra_36 | 37.63 | 0.02 | 0.02 | 0.00 | 25.32 | 0.53 | 36.19 | 0.38 | 0.00 | 0.00 | 100.09 | 0.72 |
| Tephra_37 | 37.50 | 0.02 | 0.04 | 0.00 | 26.38 | 0.60 | 35.01 | 0.43 | 0.02 | 0.01 | 100.01 | 0.70 |
| Tephra_38 | 37.60 | 0.02 | 0.04 | 0.00 | 25.85 | 0.55 | 35.62 | 0.41 | 0.03 | 0.00 | 100.12 | 0.71 |
| Tephra_39 | 37.63 | 0.00 | 0.04 | 0.01 | 25.29 | 0.60 | 36.18 | 0.31 | 0.02 | 0.00 | 100.08 | 0.72 |
| Tephra_40 | 38.07 | 0.00 | 0.02 | 0.00 | 24.58 | 0.48 | 36.88 | 0.33 | 0.00 | 0.04 | 100.41 | 0.73 |
| Tephra_41 | 37.53 | 0.03 | 0.04 | 0.01 | 25.89 | 0.60 | 35.65 | 0.42 | 0.01 | 0.03 | 100.21 | 0.71 |
| Tephra_42 | 37.47 | 0.05 | 0.03 | 0.00 | 27.44 | 0.58 | 34.18 | 0.43 | 0.00 | 0.03 | 100.22 | 0.69 |
| Tephra_43 | 37.17 | 0.03 | 0.05 | 0.01 | 27.11 | 0.60 | 34.12 | 0.44 | 0.02 | 0.02 | 99.57 | 0.69 |
| Tephra_44 | 37.04 | 0.01 | 0.01 | 0.01 | 28.62 | 0.62 | 34.72 | 0.33 | 0.01 | 0.04 | 101.41 | 0.68 |
| Tephra_45 | 37.60 | 0.02 | 0.01 | 0.00 | 27.10 | 0.60 | 36.15 | 0.38 | 0.02 | 0.03 | 101.91 | 0.70 |
| Lava_1 | 38.21 | 0.02 | 0.04 | 0.00 | 25.21 | 0.54 | 36.10 | 0.39 | 0.04 | 0.00 | 100.56 | 0.72 |
| Lava_2 | 38.07 | 0.03 | 0.03 | 0.00 | 25.26 | 0.56 | 35.96 | 0.42 | 0.03 | 0.00 | 100.37 | 0.72 |
| Lava_3 | 37.98 | 0.02 | 0.03 | 0.04 | 25.35 | 0.55 | 35.96 | 0.41 | 0.01 | 0.00 | 100.35 | 0.72 |
| Lava_4 | 38.02 | 0.04 | 0.02 | 0.00 | 25.73 | 0.57 | 35.35 | 0.49 | 0.00 | 0.00 | 100.24 | 0.71 |
| Lava_5 | 38.31 | 0.05 | 0.03 | 0.00 | 25.32 | 0.56 | 35.77 | 0.41 | 0.03 | 0.00 | 100.49 | 0.72 |
| Lava_6 | 38.10 | 0.03 | 0.05 | 0.00 | 25.18 | 0.59 | 35.61 | 0.43 | 0.02 | 0.00 | 100.01 | 0.72 |
| Lava_7 | 37.87 | 0.04 | 0.04 | 0.00 | 25.32 | 0.58 | 35.99 | 0.42 | 0.02 | 0.00 | 100.29 | 0.72 |
| Lava_8 | 38.08 | 0.06 | 0.03 | 0.03 | 25.94 | 0.59 | 35.10 | 0.48 | 0.03 | 0.00 | 100.36 | 0.71 |
| Lava_9 | 37.59 | 0.02 | 0.04 | 0.00 | 27.35 | 0.64 | 34.41 | 0.40 | 0.01 | 0.00 | 100.46 | 0.69 |
| Lava_10 | 37.73 | 0.04 | 0.05 | 0.00 | 27.00 | 0.62 | 34.77 | 0.45 | 0.02 | 0.00 | 100.68 | 0.70 |
| Lava_11 | 38.12 | 0.02 | 0.02 | 0.00 | 25.48 | 0.61 | 36.16 | 0.40 | 0.06 | 0.00 | 100.87 | 0.72 |
| Lava_12 | 37.99 | 0.02 | 0.04 | 0.00 | 25.44 | 0.61 | 35.61 | 0.44 | 0.03 | 0.00 | 100.20 | 0.71 |
| Analysis | SiO2 | TiO2 | Al2O3 | Cr2O3 | FeO | MnO | MgO | CaO | Na2O | NiO | Total | Fo |
| Lava_13 | 37.83 | 0.01 | 0.04 | 0.01 | 25.26 | 0.58 | 36.02 | 0.40 | 0.01 | 0.00 | 100.16 | 0.72 |
| Lava_14 | 37.84 | 0.02 | 0.05 | 0.00 | 25.36 | 0.62 | 35.96 | 0.43 | 0.00 | 0.00 | 100.28 | 0.72 |
| Lava_15 | 37.92 | 0.05 | 0.05 | 0.02 | 25.39 | 0.57 | 35.79 | 0.45 | 0.01 | 0.00 | 100.26 | 0.72 |
| Lava_16 | 38.11 | 0.03 | 0.03 | 0.01 | 23.76 | 0.59 | 36.95 | 0.41 | 0.02 | 0.00 | 99.90 | 0.73 |
| Lava_17 | 38.30 | 0.04 | 0.05 | 0.01 | 23.97 | 0.58 | 37.06 | 0.44 | 0.02 | 0.00 | 100.46 | 0.73 |
| Lava_18 | 39.07 | 0.02 | 0.05 | 0.00 | 20.97 | 0.43 | 40.38 | 0.30 | - | 0.04 | 101.26 | 0.77 |
| Lava_19 | 38.90 | 0.05 | 0.03 | 0.01 | 20.69 | 0.42 | 40.65 | 0.31 | 0.02 | 0.03 | 101.10 | 0.78 |
| Lava_20 | 38.49 | - | 0.04 | - | 22.50 | 0.46 | 38.89 | 0.32 | 0.00 | 0.04 | 100.74 | 0.75 |
| Lava_21 | 39.06 | 0.02 | 0.02 | 0.02 | 21.11 | 0.44 | 40.32 | 0.38 | 0.02 | 0.03 | 101.42 | 0.77 |
| Lava_22 | 38.43 | 0.03 | 0.03 | 0.00 | 24.33 | 0.54 | 37.58 | 0.31 | 0.00 | 0.03 | 101.29 | 0.73 |
| Lava_23 | 38.30 | 0.03 | 0.03 | - | 25.34 | 0.57 | 36.84 | 0.39 | 0.02 | 0.03 | 101.55 | 0.72 |
| Lava_24 | 38.12 | 0.11 | 0.03 | - | 25.70 | 0.57 | 36.32 | 0.43 | 0.03 | 0.02 | 101.32 | 0.72 |
| Lava_25 | 38.10 | 0.05 | 0.03 | 0.00 | 25.07 | 0.61 | 36.66 | 0.42 | 0.02 | - | 100.97 | 0.72 |
| Lava_26 | 39.86 | - | 0.04 | - | 17.63 | 0.29 | 42.99 | 0.26 | 0.02 | 0.05 | 101.13 | 0.81 |
| Lava_27 | 39.41 | 0.02 | 0.04 | 0.01 | 18.69 | 0.33 | 42.22 | 0.25 | 0.021 | 0.06 | 101.05 | 0.80 |
| Lava_28 | 38.93 | 0.02 | 0.01 | 0.01 | 21.35 | 0.43 | 40.36 | 0.22 | 0.006 | 0.03 | 101.37 | 0.77 |
| Lava_29 | 39.34 | - | 0.02 | 0.01 | 18.87 | 0.35 | 42.41 | 0.24 | 0.007 | 0.07 | 101.32 | 0.80 |
| Lava_30 | 39.77 | 0.01 | 0.01 | - | 17.34 | 0.30 | 43.68 | 0.24 | 0.015 | 0.06 | 101.42 | 0.82 |
| Lava_31 | 39.21 | 0.03 | 0.04 | 0.01 | 19.63 | 0.37 | 41.59 | 0.27 | 0.013 | 0.05 | 101.21 | 0.79 |
| Lava_32 | 38.63 | - | 0.02 | - | 24.67 | 0.54 | 37.42 | 0.39 | - | 0.02 | 101.69 | 0.73 |
| Lava_33 | 38.54 | 0.02 | 0.01 | 0.00 | 24.69 | 0.55 | 37.45 | 0.36 | - | - | 101.63 | 0.73 |
| Lava_34 | 38.42 | 0.04 | 0.04 | 0.01 | 25.33 | 0.60 | 36.58 | 0.38 | 0.014 | 0.01 | 101.42 | 0.72 |
| Lava_35 | 39.63 | 0.01 | 0.01 | 0.02 | 17.33 | 0.27 | 43.33 | 0.24 | - | 0.05 | 100.90 | 0.82 |
| Lava_36 | 38.28 | 0.05 | 0.04 | - | 25.45 | 0.63 | 36.29 | 0.36 | 0.009 | 0.03 | 101.13 | 0.72 |
| Lava_37 | 39.64 | 0.01 | 0.02 | - | 17.78 | 0.31 | 43.15 | 0.26 | - | 0.06 | 101.22 | 0.81 |
| Lava_38 | 39.45 | 0.02 | 0.02 | - | 17.93 | 0.31 | 42.97 | 0.24 | 0.002 | 0.05 | 101.00 | 0.81 |
| Lava_39 | 38.63 | 0.06 | 0.04 | - | 22.55 | 0.46 | 38.93 | 0.26 | 0.006 | 0.02 | 100.95 | 0.75 |
| Lava_40 | 39.52 | 0.00 | 0.03 | 0.00 | 18.12 | 0.32 | 42.59 | 0.23 | 0.002 | 0.06 | 100.88 | 0.81 |
| Lava_41 | 39.19 | 0.02 | 0.01 | 0.02 | 20.37 | 0.38 | 40.77 | 0.25 | - | 0.04 | 101.05 | 0.78 |
| Analysis | SiO2 | TiO2 | Al2O3 | Cr2O3 | FeO | MnO | MgO | CaO | Na2O | NiO | Total | Fo |
| Lava_42 | 38.08 | 0.05 | 0.04 | - | 25.70 | 0.64 | 35.82 | 0.36 | 0.002 | 0.03 | 100.72 | 0.71 |
| Lava_43 | 38.04 | 0.05 | 0.03 | 0.00 | 25.66 | 0.63 | 36.41 | 0.40 | 0.014 | 0.01 | 101.24 | 0.72 |
| Lava_44 | 38.05 | 0.06 | 0.04 | 0.00 | 25.84 | 0.58 | 36.33 | 0.41 | 0.027 | 0.03 | 101.37 | 0.71 |
| Lava_45 | 38.01 | 0.05 | 0.04 | 0.00 | 25.74 | 0.63 | 36.52 | 0.43 | 0.018 | 0.02 | 101.46 | 0.72 |
| Lava_46 | 38.04 | 0.08 | 0.04 | 0.00 | 25.56 | 0.59 | 36.58 | 0.43 | 0.000 | 0.01 | 101.33 | 0.72 |
| Strombolian_1 | 38.12 | 0.03 | 0.03 | 0.00 | 22.07 | 0.36 | 40.68 | 0.27 | 0.000 | 0.03 | 101.58 | 0.77 |
| Strombolian_2 | 38.04 | 0.02 | 0.03 | 0.01 | 20.28 | 0.31 | 42.09 | 0.31 | 0.000 | 0.06 | 101.16 | 0.79 |
| Strombolian_3 | 37.39 | 0.02 | 0.04 | 0.00 | 22.54 | 0.44 | 40.24 | 0.28 | 0.009 | 0.04 | 101.00 | 0.76 |
| Strombolian_4 | 37.36 | 0.02 | 0.03 | 0.01 | 21.95 | 0.43 | 40.91 | 0.27 | 0.012 | 0.07 | 101.05 | 0.77 |
| Strombolian_5 | 38.18 | 0.00 | 0.02 | 0.00 | 20.33 | 0.35 | 41.72 | 0.25 | 0.010 | 0.07 | 100.93 | 0.79 |
| Strombolian_6 | 37.85 | 0.03 | 0.03 | 0.00 | 21.08 | 0.39 | 41.18 | 0.27 | 0.030 | 0.07 | 100.94 | 0.78 |
| Strombolian_7 | 37.63 | 0.04 | 0.035 | 0.014 | 26.54 | 0.57 | 36.48 | 0.42 | 0.015 | 0.009 | 101.74 | 0.71 |
| Strombolian_8 | 37.00 | 0.01 | 0.028 | 0.000 | 26.26 | 0.59 | 36.98 | 0.40 | 0.010 | 0.014 | 101.30 | 0.72 |
| Strombolian_9 | 37.03 | 0.06 | 0.047 | 0.000 | 26.43 | 0.52 | 36.50 | 0.44 | 0.010 | 0.028 | 101.07 | 0.71 |
| Strombolian_10 | 37.45 | 0.02 | 0.027 | 0.003 | 21.31 | 0.38 | 41.47 | 0.28 | 0.000 | 0.024 | 100.96 | 0.78 |
| Strombolian_11 | 37.94 | 0.00 | 0.035 | 0.000 | 21.47 | 0.43 | 40.53 | 0.27 | 0.010 | 0.047 | 100.73 | 0.77 |
| Strombolian_12 | 37.95 | 0.03 | 0.039 | 0.000 | 26.13 | 0.59 | 35.65 | 0.39 | 0.016 | 0.005 | 100.80 | 0.71 |
| Strombolian_13 | 37.72 | 0.07 | 0.073 | 0.025 | 26.38 | 0.55 | 35.18 | 0.41 | 0.038 | 0.000 | 100.47 | 0.70 |
| Strombolian_14 | 37.06 | 0.06 | 0.058 | 0.000 | 28.43 | 0.69 | 32.99 | 0.49 | 0.025 | 0.000 | 99.83 | 0.67 |
| Strombolian_15 | 37.77 | 0.02 | 0.030 | 0.007 | 26.11 | 0.57 | 35.27 | 0.40 | 0.036 | 0.019 | 100.24 | 0.71 |
| Strombolian_16 | 37.39 | 0.05 | 0.071 | 0.006 | 27.15 | 0.59 | 34.39 | 0.40 | 0.015 | 0.020 | 100.08 | 0.69 |
| Strombolian_17 | 38.37 | 0.03 | 0.043 | 0.000 | 22.96 | 0.46 | 37.04 | 0.38 | 0.019 | 0.012 | 99.32 | 0.74 |
| Strombolian_18 | 38.29 | 0.03 | 0.042 | 0.021 | 23.34 | 0.47 | 36.37 | 0.41 | 0.004 | 0.010 | 98.99 | 0.74 |
| Strombolian_19 | 37.58 | 0.02 | 0.009 | 0.000 | 26.92 | 0.61 | 33.66 | 0.41 | 0.042 | 0.000 | 99.26 | 0.69 |
| Strombolian_20 | 37.46 | 0.01 | 0.050 | 0.000 | 27.09 | 0.61 | 34.02 | 0.39 | 0.016 | 0.032 | 99.68 | 0.69 |
| Strombolian_21 | 37.55 | 0.05 | 0.048 | 0.008 | 26.89 | 0.60 | 34.04 | 0.43 | 0.006 | 0.010 | 99.64 | 0.69 |
| Strombolian_22 | 37.69 | 0.05 | 0.025 | 0.000 | 26.98 | 0.57 | 33.38 | 0.43 | 0.008 | 0.000 | 99.14 | 0.69 |
| Strombolian_23 | 37.81 | 0.04 | 0.037 | 0.000 | 25.56 | 0.54 | 34.81 | 0.44 | 0.030 | 0.016 | 99.27 | 0.71 |
| Strombolian_24 | 37.64 | 0.01 | 0.014 | 0.031 | 25.11 | 0.56 | 36.34 | 0.34 | 0.000 | 0.015 | 100.07 | 0.72 |
| Analysis | SiO2 | TiO2 | Al2O3 | Cr2O3 | FeO | MnO | MgO | CaO | Na2O | NiO | Total | fo |
| Strombolian_25 | 37.33 | 0.02 | 0.025 | 0.000 | 25.56 | 0.59 | 36.06 | 0.43 | 0.000 | 0.000 | 100.01 | 0.72 |
| Strombolian_26 | 37.63 | 0.02 | 0.045 | 0.000 | 25.22 | 0.55 | 35.85 | 0.44 | 0.020 | 0.000 | 99.80 | 0.72 |
| Strombolian_27 | 37.59 | 0.00 | 0.023 | 0.000 | 24.81 | 0.53 | 36.40 | 0.35 | 0.017 | 0.021 | 99.76 | 0.72 |
| Strombolian_28 | 37.93 | 0.03 | 0.030 | 0.000 | 24.77 | 0.55 | 36.30 | 0.40 | 0.038 | 0.007 | 100.06 | 0.72 |
| Strombolian_29 | 37.68 | 0.03 | 0.022 | 0.000 | 23.86 | 0.55 | 37.29 | 0.43 | 0.029 | 0.043 | 99.93 | 0.74 |
| Strombolian_30 | 37.97 | 0.02 | 0.040 | 0.000 | 23.31 | 0.51 | 37.53 | 0.42 | 0.000 | 0.000 | 99.80 | 0.74 |
| Strombolian_31 | 38.16 | 0.05 | 0.030 | 0.000 | 24.27 | 0.49 | 36.57 | 0.46 | 0.027 | 0.000 | 100.06 | 0.73 |
| Strombolian_32 | 37.49 | 0.04 | 0.047 | 0.006 | 26.91 | 0.63 | 34.01 | 0.46 | 0.005 | 0.000 | 99.60 | 0.69 |
| Strombolian_33 | 38.04 | 0.02 | 0.035 | 0.014 | 20.28 | 0.32 | 42.09 | 0.32 | 0.000 | 0.064 | 101.16 | 0.79 |
| Xenolith_1 | 38.12 | 0.03 | 0.017 | 0.000 | 25.05 | 0.55 | 36.26 | 0.37 | 0.042 | 0.000 | 100.44 | 0.72 |
| Xenolith_2 | 37.77 | 0.00 | 0.023 | 0.007 | 24.67 | 0.52 | 36.64 | 0.37 | 0.007 | 0.000 | 100.02 | 0.73 |
| Xenolith_3 | 38.35 | 0.03 | 0.027 | 0.000 | 21.70 | 0.43 | 39.04 | 0.43 | 0.000 | 0.000 | 100.00 | 0.76 |
| Xenolith_4 | 37.80 | 0.04 | 0.025 | 0.000 | 26.04 | 0.63 | 35.36 | 0.45 | 0.032 | 0.000 | 100.38 | 0.71 |
| Xenolith_5 | 38.33 | 0.04 | 0.023 | 0.020 | 22.72 | 0.48 | 38.01 | 0.39 | 0.006 | 0.000 | 100.01 | 0.75 |
| Xenolith_6 | 38.54 | 0.04 | 0.032 | 0.010 | 22.13 | 0.45 | 38.74 | 0.42 | 0.029 | 0.000 | 100.39 | 0.76 |
| Xenolith_7 | 37.69 | 0.04 | 0.060 | 0.000 | 26.35 | 0.60 | 34.94 | 0.45 | 0.020 | 0.000 | 100.16 | 0.70 |
| Xenolith_8 | 38.97 | 0.02 | 0.033 | 0.011 | 18.84 | 0.35 | 41.65 | 0.31 | 0.024 | 0.000 | 100.21 | 0.80 |
| Xenolith_9 | 39.12 | 0.00 | 0.008 | 0.009 | 17.96 | 0.30 | 42.50 | 0.23 | 0.010 | 0.000 | 100.15 | 0.81 |
| Xenolith_10 | 38.05 | 0.00 | 0.031 | 0.003 | 23.58 | 0.48 | 37.66 | 0.33 | 0.000 | 0.000 | 100.15 | 0.74 |
| Xenolith_11 | 38.56 | 0.01 | 0.033 | 0.021 | 23.88 | 0.51 | 37.39 | 0.36 | 0.001 | 0.000 | 100.78 | 0.74 |
| Xenolith_12 | 37.78 | 0.02 | 0.016 | 0.000 | 25.59 | 0.56 | 35.74 | 0.41 | 0.035 | 0.000 | 100.15 | 0.71 |
| Xenolith_13 | 37.92 | 0.01 | 0.032 | 0.020 | 24.38 | 0.49 | 36.76 | 0.42 | 0.000 | 0.000 | 100.06 | 0.73 |
| Xenolith_14 | 37.69 | 0.02 | 0.013 | 0.000 | 25.55 | 0.58 | 35.60 | 0.41 | 0.003 | 0.000 | 99.87 | 0.71 |
| Xenolith_15 | 38.22 | 0.03 | 0.028 | 0.000 | 22.64 | 0.50 | 38.51 | 0.28 | 0.000 | 0.000 | 100.21 | 0.75 |
| Xenolith_16 | 37.99 | 0.02 | 0.030 | 0.003 | 24.15 | 0.51 | 36.98 | 0.35 | 0.029 | 0.000 | 100.07 | 0.73 |

Table S2 - Olivine Elemental Data

| Analysis | SiO2 | TiO2 | Al2O3 | Cr2O3 | FeO | MnO | MgO | CaO | Na2O | NiO | Total | Wo | En | Fs |
| --- | --- | --- | --- | --- | --- | --- | --- | --- | --- | --- | --- | --- | --- | --- |
| Lava_1 | 50.45 | 1.21 | 3.63 | 0.01 | 7.77 | 0.20 | 13.86 | 21.46 | 0.46 | 0.01 | 99.06 | 45.85 | 41.20 | 12.96 |
| Lava_2 | 47.98 | 2.02 | 5.66 | - | 8.39 | 0.20 | 12.87 | 21.51 | 0.55 | - | 99.18 | 46.80 | 38.96 | 14.25 |
| Lava_3 | 48.7 | 1.74 | 5.07 | 0.01 | 8.32 | 0.21 | 13.01 | 21.56 | 0.53 | - | 99.15 | 46.71 | 39.22 | 14.07 |
| Lava_4 | 48.68 | 1.78 | 5.19 | 0.02 | 8.28 | 0.19 | 12.89 | 21.63 | 0.52 | 0.00 | 99.18 | 46.99 | 38.97 | 14.04 |
| Lava_5 | 48.66 | 1.73 | 5.2 | 0.00 | 8.04 | 0.19 | 13.21 | 21.81 | 0.48 | 0.01 | 99.33 | 46.94 | 39.56 | 13.51 |
| Lava_6 | 48.56 | 1.84 | 5.15 | 0.02 | 8.55 | 0.18 | 12.91 | 21.63 | 0.55 | 0.01 | 99.40 | 46.75 | 38.82 | 14.42 |
| Lava_7 | 48.46 | 1.90 | 5.35 | - | 8.58 | 0.19 | 12.82 | 21.54 | 0.55 | - | 99.39 | 46.75 | 38.71 | 14.54 |
| Lava_8 | 48.6 | 1.87 | 5.28 | 0.01 | 8.56 | 0.21 | 12.75 | 21.55 | 0.54 | 0.01 | 99.39 | 46.88 | 38.59 | 14.53 |
| Lava_9 | 48.71 | 1.75 | 5.21 | 0.02 | 8.17 | 0.19 | 13.11 | 21.63 | 0.55 | - | 99.34 | 46.77 | 39.44 | 13.79 |
| Lava_10 | 48.29 | 1.88 | 5.62 | 0.02 | 8.36 | 0.18 | 12.77 | 21.45 | 0.51 | 0.02 | 99.10 | 46.89 | 38.84 | 14.27 |
| Lava_11 | 47.83 | 2.07 | 5.72 | - | 8.77 | 0.19 | 12.66 | 21.54 | 0.51 | 0.00 | 99.29 | 46.83 | 38.29 | 14.88 |
| Lava_12 | 50.42 | 1.26 | 3.47 | 0.01 | 8.06 | 0.23 | 13.91 | 21.28 | 0.43 | - | 99.07 | 45.35 | 41.24 | 13.41 |
| Lava_13 | 50.63 | 1.38 | 3.4 | 0.01 | 8.16 | 0.26 | 13.87 | 21.11 | 0.45 | 0.02 | 99.29 | 45.13 | 41.26 | 13.62 |
| Lava_14 | 50.82 | 1.21 | 3.35 | 0.01 | 7.94 | 0.21 | 13.85 | 21.53 | 0.51 | 0.01 | 99.45 | 45.81 | 41.00 | 13.19 |
| Lava_15 | 50.44 | 1.23 | 3.65 | 0.01 | 8.06 | 0.21 | 13.79 | 21.29 | 0.53 | 0.01 | 99.21 | 45.52 | 41.03 | 13.45 |
| Lava_16 | 48.68 | 1.90 | 5.14 | 0.02 | 8.67 | 0.23 | 12.73 | 21.49 | 0.52 | - | 99.38 | 46.75 | 38.53 | 14.72 |
| Lava_17 | 48.93 | 1.85 | 4.81 | 0.01 | 8.69 | 0.21 | 12.87 | 21.52 | 0.57 | 0.00 | 99.46 | 46.57 | 38.75 | 14.68 |
| Lava_18 | 48.99 | 1.74 | 4.73 | 0.00 | 8.62 | 0.23 | 12.68 | 21.58 | 0.54 | - | 99.10 | 46.96 | 38.39 | 14.64 |
| Lava_19 | 48.78 | 1.79 | 5.03 | 0.00 | 8.68 | 0.21 | 12.82 | 21.63 | 0.54 | - | 99.48 | 46.77 | 38.57 | 14.65 |
| Lava_20 | 46.96 | 2.26 | 6.91 | 0.01 | 8.7 | 0.15 | 12.3 | 21.61 | 0.50 | 0.01 | 99.41 | 47.48 | 37.60 | 14.92 |
| Lava_21 | 48.75 | 1.77 | 5.09 | 0.00 | 8.86 | 0.26 | 13.07 | 21.45 | 0.52 | 0.00 | 99.76 | 46.08 | 39.07 | 14.86 |
| Lava_22 | 50.38 | 1.35 | 3.8 | 0.02 | 8.21 | 0.22 | 13.75 | 21.54 | 0.52 | - | 99.78 | 45.75 | 40.64 | 13.61 |
| Lava_23 | 50.66 | 1.26 | 3.54 | 0.00 | 8.16 | 0.22 | 13.7 | 21.56 | 0.57 | - | 99.66 | 45.88 | 40.57 | 13.55 |
| Lava_24 | 51.1 | 1.27 | 3.12 | 0.00 | 8.3 | 0.26 | 14.01 | 21.21 | 0.58 | 0.02 | 99.87 | 44.95 | 41.32 | 13.73 |
| Lava_25 | 50.48 | 1.47 | 3.47 | 0.00 | 8.96 | 0.30 | 13.39 | 20.97 | 0.60 | 0.02 | 99.66 | 45.01 | 39.98 | 15.01 |
| Lava_26 | 49.97 | 1.55 | 4.37 | 0.02 | 8.22 | 0.22 | 13.39 | 21.66 | 0.53 | - | 99.94 | 46.37 | 39.89 | 13.74 |
| Lava_27 | 50.32 | 1.12 | 4.18 | 0.04 | 6.98 | 0.16 | 14.34 | 22.63 | 0.33 | 0.00 | 100.10 | 47.12 | 41.54 | 11.34 |
| Lava_28 | 50.22 | 1.22 | 4.15 | 0.09 | 7.68 | 0.18 | 14.22 | 21.77 | 0.37 | - | 99.91 | 45.78 | 41.61 | 12.61 |
| Lava_29 | 49.58 | 1.61 | 4.54 | - | 8.37 | 0.22 | 13.16 | 21.65 | 0.52 | 0.01 | 99.66 | 46.57 | 39.38 | 14.05 |
| Analysis | SiO2 | TiO2 | Al2O3 | Cr2O3 | FeO | MnO | MgO | CaO | Na2O | NiO | Total | Wo | En | Fs |
| Lava_30 | 49.64 | 1.56 | 4.72 | - | 8.25 | 0.20 | 13.35 | 21.62 | 0.57 | 0.00 | 99.91 | 46.36 | 39.83 | 13.81 |
| Lava_31 | 47.41 | 2.33 | 6.31 | 0.01 | 8.96 | 0.23 | 12.39 | 21.4 | 0.56 | - | 99.61 | 46.90 | 37.78 | 15.33 |
| Lava_32 | 47.81 | 2.17 | 5.83 | 0.01 | 8.74 | 0.20 | 12.67 | 21.62 | 0.55 | 0.01 | 99.60 | 46.93 | 38.26 | 14.81 |
| Lava_33 | 49.05 | 1.57 | 4.73 | 0.00 | 8.07 | 0.20 | 13.32 | 21.58 | 0.55 | 0.00 | 99.07 | 46.50 | 39.93 | 13.57 |
| Lava_34 | 47.35 | 2.22 | 6.21 | - | 8.72 | 0.19 | 12.61 | 21.7 | 0.50 | 0.01 | 99.51 | 47.12 | 38.10 | 14.78 |
| Lava_35 | 47.87 | 1.99 | 5.83 | - | 8.55 | 0.18 | 12.72 | 21.5 | 0.57 | 0.00 | 99.22 | 46.87 | 38.58 | 14.55 |
| Lava_36 | 48.62 | 1.80 | 5.4 | 0.00 | 8.34 | 0.21 | 13.09 | 21.74 | 0.49 | 0.01 | 99.69 | 46.79 | 39.20 | 14.01 |
| Lava_37 | 47.78 | 2.06 | 5.77 | 0.01 | 8.72 | 0.19 | 12.6 | 21.54 | 0.55 | 0.02 | 99.24 | 46.95 | 38.21 | 14.84 |
| Lava_38 | 49.41 | 1.61 | 4.43 | 0.01 | 8.53 | 0.21 | 13.57 | 21.58 | 0.44 | 0.00 | 99.80 | 45.80 | 40.07 | 14.13 |
| Lava_39 | 50.5 | 1.31 | 3.55 | 0.02 | 8.05 | 0.23 | 14.06 | 21.44 | 0.44 | 0.00 | 99.60 | 45.34 | 41.37 | 13.29 |
| Lava_40 | 51 | 1.23 | 3.39 | 0.01 | 8.13 | 0.22 | 14.31 | 21.3 | 0.45 | 0.01 | 100.05 | 44.79 | 41.87 | 13.34 |
| Lava_41 | 50.96 | 1.23 | 3.3 | - | 7.99 | 0.20 | 14.21 | 21.53 | 0.44 | - | 99.85 | 45.29 | 41.59 | 13.12 |
| Lava_42 | 47.79 | 2.08 | 5.6 | - | 8.74 | 0.20 | 12.6 | 21.36 | 0.49 | 0.00 | 98.87 | 46.73 | 38.35 | 14.92 |
| Lava_43 | 48.42 | 1.96 | 5.49 | 0.01 | 8.46 | 0.21 | 12.8 | 21.58 | 0.51 | 0.00 | 99.45 | 46.92 | 38.72 | 14.36 |
| Lava_44 | 48.02 | 2.05 | 5.73 | - | 8.7 | 0.18 | 12.78 | 21.63 | 0.46 | 0.01 | 99.55 | 46.82 | 38.49 | 14.70 |
| Lava_45 | 47.94 | 2.12 | 5.71 | 0.01 | 8.69 | 0.20 | 12.66 | 21.65 | 0.48 | 0.00 | 99.47 | 47.02 | 38.25 | 14.73 |
| Lava_46 | 47.92 | 2.15 | 5.84 | 0.00 | 8.81 | 0.19 | 12.5 | 21.35 | 0.47 | 0.01 | 99.24 | 46.80 | 38.13 | 15.07 |
| Lava_47 | 48.54 | 1.84 | 5.49 | - | 8.68 | 0.19 | 12.85 | 21.57 | 0.52 | 0.01 | 99.69 | 46.66 | 38.68 | 14.66 |
| Lava_48 | 49.46 | 1.63 | 4.94 | 0.01 | 8.39 | 0.21 | 13.05 | 21.61 | 0.53 | - | 99.82 | 46.66 | 39.20 | 14.14 |
| Lava_49 | 49.32 | 1.65 | 4.87 | - | 8.45 | 0.21 | 13 | 21.67 | 0.51 | 0.01 | 99.68 | 46.75 | 39.02 | 14.23 |
| Lava_50 | 48.90 | 1.81 | 5.00 | 0.02 | 8.63 | 0.21 | 12.75 | 21.57 | 0.54 | 0.00 | 99.43 | 46.84 | 38.53 | 14.63 |
| Lava_51 | 48.88 | 1.77 | 5.02 | 0.00 | 8.33 | 0.22 | 13 | 21.83 | 0.50 | - | 99.55 | 47.03 | 38.97 | 14.01 |
| Lava_52 | 48.91 | 1.83 | 5.12 | 0.01 | 8.46 | 0.22 | 12.9 | 21.48 | 0.54 | 0.01 | 99.48 | 46.66 | 38.99 | 14.35 |
| Lava_53 | 49.45 | 1.65 | 4.69 | - | 7.94 | 0.26 | 13.28 | 21.7 | 0.59 | 0.00 | 99.56 | 46.79 | 39.84 | 13.36 |
| Lava_54 | 48.85 | 1.77 | 5.1 | - | 8.41 | 0.23 | 12.96 | 21.57 | 0.53 | 0.00 | 99.43 | 46.72 | 39.06 | 14.22 |
| Lava_55 | 49.25 | 1.68 | 4.8 | - | 8.33 | 0.21 | 13.13 | 21.65 | 0.50 | - | 99.55 | 46.64 | 39.36 | 14.01 |
| Lava_56 | 49.30 | 1.67 | 4.8 | - | 8.19 | 0.22 | 13.07 | 21.5 | 0.49 | - | 99.24 | 46.66 | 39.47 | 13.87 |
| Lava_57 | 48.96 | 1.80 | 5.05 | 0.02 | 8.56 | 0.24 | 12.81 | 21.43 | 0.54 | 0.00 | 99.42 | 46.65 | 38.80 | 14.55 |
| Lava_58 | 47.14 | 2.50 | 8.56 | 0.02 | 8.86 | 0.17 | 11.79 | 20.98 | 0.79 | - | 100.81 | 47.36 | 37.03 | 15.61 |
| Analysis | SiO2 | TiO2 | Al2O3 | Cr2O3 | FeO | MnO | MgO | CaO | Na2O | NiO | Total | Wo | En | Fs |
| Lava_59 | 44.28 | 2.58 | 9.07 | 0.11 | 8.85 | 0.11 | 11.47 | 23.19 | 0.30 | - | 99.97 | 50.35 | 34.65 | 15.00 |
| Lava_60 | 50.21 | 1.28 | 3.81 | 0.02 | 7.73 | 0.21 | 14.1 | 21.97 | 0.49 | 0.00 | 99.83 | 46.13 | 41.20 | 12.67 |
| Lava_61 | 49.88 | 1.31 | 4.13 | - | 7.76 | 0.18 | 13.91 | 21.87 | 0.49 | 0.02 | 99.56 | 46.26 | 40.93 | 12.81 |
| Lava_62 | 48.26 | 1.78 | 5.54 | 0.01 | 8.33 | 0.18 | 12.87 | 21.9 | 0.51 | - | 99.37 | 47.29 | 38.67 | 14.04 |
| Lava_63 | 47.48 | 2.25 | 6.28 | 0.01 | 8.6 | 0.20 | 12.51 | 21.58 | 0.50 | 0.02 | 99.43 | 47.22 | 38.09 | 14.69 |
| Lava_64 | 48.72 | 1.68 | 5.22 | 0.03 | 8.13 | 0.18 | 13.16 | 21.65 | 0.55 | 0.02 | 99.33 | 46.75 | 39.54 | 13.70 |
| Lava_65 | 50.30 | 1.29 | 3.9 | 0.01 | 7.77 | 0.22 | 13.79 | 21.61 | 0.50 | 0.00 | 99.39 | 46.11 | 40.94 | 12.94 |
| Lava_66 | 49.33 | 1.53 | 4.85 | 0.01 | 7.94 | 0.19 | 13.3 | 21.66 | 0.50 | 0.01 | 99.32 | 46.72 | 39.91 | 13.37 |
| Lava_67 | 50.08 | 1.27 | 3.89 | - | 7.51 | 0.20 | 13.88 | 21.88 | 0.45 | - | 99.16 | 46.50 | 41.04 | 12.46 |
| Lava_68 | 50.22 | 1.29 | 3.83 | 0.00 | 7.7 | 0.19 | 14.02 | 21.56 | 0.43 | - | 99.24 | 45.80 | 41.44 | 12.77 |
| Lava_69 | 49.83 | 1.38 | 4.16 | 0.01 | 7.62 | 0.19 | 13.78 | 21.61 | 0.43 | - | 99.01 | 46.24 | 41.03 | 12.73 |
| Lava_70 | 49.98 | 1.21 | 4.24 | 0.01 | 7.41 | 0.18 | 13.94 | 22.01 | 0.37 | - | 99.35 | 46.64 | 41.10 | 12.26 |
| Lava_71 | 50.26 | 1.17 | 3.99 | - | 7.56 | 0.18 | 14.03 | 21.78 | 0.38 | 0.02 | 99.37 | 46.14 | 41.36 | 12.50 |
| Lava_72 | 50.56 | 1.20 | 3.53 | 0.02 | 7.58 | 0.23 | 14.08 | 21.61 | 0.45 | 0.00 | 99.26 | 45.86 | 41.58 | 12.56 |
| Lava_73 | 50.32 | 1.30 | 3.62 | - | 7.74 | 0.21 | 13.94 | 21.76 | 0.45 | - | 99.33 | 46.10 | 41.10 | 12.80 |
| Lava_74 | 49.88 | 1.44 | 4.41 | 0.02 | 7.9 | 0.20 | 13.57 | 21.86 | 0.47 | 0.01 | 99.76 | 46.60 | 40.25 | 13.15 |
| Lava_75 | 49.73 | 1.36 | 4.4 | 0.03 | 7.91 | 0.19 | 13.57 | 21.69 | 0.46 | 0.01 | 99.35 | 46.40 | 40.39 | 13.21 |
| Lava_76 | 50.5 | 1.26 | 3.57 | 0.00 | 7.63 | 0.20 | 14.08 | 21.82 | 0.43 | 0.01 | 99.51 | 46.07 | 41.36 | 12.57 |
| Lava_77 | 50.04 | 1.30 | 3.99 | 0.02 | 7.72 | 0.19 | 13.82 | 21.91 | 0.45 | - | 99.45 | 46.45 | 40.77 | 12.78 |
| Lava_78 | 50.13 | 1.29 | 4.06 | 0.00 | 7.83 | 0.18 | 13.74 | 21.65 | 0.46 | - | 99.34 | 46.18 | 40.78 | 13.04 |
| Lava_79 | 50.29 | 1.27 | 3.81 | - | 7.8 | 0.19 | 13.85 | 21.6 | 0.43 | 0.00 | 99.24 | 46.00 | 41.04 | 12.97 |
| Lava_80 | 50.08 | 1.31 | 4.06 | 0.00 | 7.74 | 0.21 | 13.83 | 21.66 | 0.43 | 0.01 | 99.33 | 46.14 | 40.99 | 12.87 |
| Lava_81 | 49.97 | 1.34 | 4.17 | - | 7.9 | 0.20 | 13.74 | 21.75 | 0.44 | - | 99.51 | 46.24 | 40.65 | 13.11 |
| Lava_82 | 50.11 | 1.34 | 4.06 | 0.01 | 7.97 | 0.20 | 13.69 | 21.65 | 0.50 | 0.01 | 99.53 | 46.14 | 40.60 | 13.26 |
| Lava_83 | 49.36 | 1.49 | 4.67 | - | 8.16 | 0.20 | 13.2 | 21.65 | 0.50 | 0.02 | 99.25 | 46.67 | 39.60 | 13.73 |
| Lava_84 | 50.24 | 1.28 | 3.62 | 0.00 | 7.67 | 0.25 | 13.8 | 21.76 | 0.45 | - | 99.07 | 46.35 | 40.90 | 12.75 |
| Lava_85 | 49.64 | 1.44 | 4.55 | 0.01 | 7.95 | 0.20 | 13.39 | 21.76 | 0.55 | 0.03 | 99.53 | 46.70 | 39.98 | 13.32 |
| Lava_86 | 48.44 | 1.81 | 5.48 | 0.00 | 8.87 | 0.19 | 12.71 | 21.44 | 0.52 | - | 99.46 | 46.56 | 38.40 | 15.04 |
| Lava_87 | 49.88 | 1.31 | 4.17 | - | 8.38 | 0.22 | 13.58 | 21.34 | 0.52 | 0.00 | 99.40 | 45.62 | 40.39 | 13.98 |
| Analysis | SiO2 | TiO2 | Al2O3 | Cr2O3 | FeO | MnO | MgO | CaO | Na2O | NiO | Total | Wo | En | Fs |
| Lava_88 | 46.99 | 2.27 | 6.61 | 0.01 | 8.84 | 0.20 | 12.29 | 21.47 | 0.53 | 0.00 | 99.21 | 47.22 | 37.61 | 15.17 |
| Lava_89 | 49.49 | 1.47 | 4.41 | 0.02 | 8.55 | 0.20 | 13.39 | 21.38 | 0.51 | - | 99.42 | 45.80 | 39.91 | 14.30 |
| Lava_90 | 47.18 | 2.27 | 6.42 | 0.02 | 8.59 | 0.20 | 12.37 | 21.63 | 0.51 | 0.02 | 99.21 | 47.49 | 37.79 | 14.72 |
| Lava_91 | 46.78 | 2.13 | 6.78 | 0.02 | 8.33 | 0.15 | 12.41 | 21.77 | 0.42 | - | 98.79 | 47.80 | 37.92 | 14.28 |
| Lava_92 | 47.86 | 2.09 | 5.8 | - | 8.63 | 0.18 | 12.64 | 21.65 | 0.53 | 0.00 | 99.38 | 47.09 | 38.26 | 14.65 |
| Lava_93 | 48.44 | 1.68 | 5.46 | - | 8.37 | 0.20 | 12.66 | 21.38 | 0.60 | - | 98.80 | 46.96 | 38.69 | 14.35 |
| Lava_94 | 48.65 | 1.67 | 5.43 | 0.00 | 8.4 | 0.23 | 12.61 | 21.21 | 0.60 | 0.01 | 98.82 | 46.81 | 38.72 | 14.47 |
| Lava_95 | 48.67 | 1.66 | 5.46 | 0.00 | 8.5 | 0.19 | 12.61 | 21.1 | 0.62 | 0.01 | 98.82 | 46.60 | 38.75 | 14.65 |
| Lava_96 | 48.54 | 1.71 | 5.46 | 0.00 | 8.43 | 0.23 | 12.74 | 21.33 | 0.68 | 0.00 | 99.12 | 46.74 | 38.84 | 14.42 |
| Lava_97 | 48.55 | 1.71 | 5.4 | 0.02 | 8.33 | 0.21 | 12.9 | 21.25 | 0.64 | - | 99.01 | 46.50 | 39.27 | 14.23 |
| Lava_98 | 48.63 | 1.66 | 5.41 | - | 8.31 | 0.19 | 12.8 | 21.22 | 0.63 | - | 98.85 | 46.62 | 39.13 | 14.25 |
| Lava_99 | 48.59 | 1.66 | 5.4 | 0.01 | 8.42 | 0.20 | 12.86 | 21.32 | 0.65 | - | 99.12 | 46.56 | 39.08 | 14.35 |
| Lava_100 | 48.68 | 1.63 | 5.27 | 0.02 | 8.23 | 0.20 | 12.91 | 21.52 | 0.62 | 0.00 | 99.08 | 46.88 | 39.13 | 13.99 |
| Lava_101 | 48.71 | 1.61 | 5.05 | 0.00 | 8.16 | 0.19 | 12.98 | 21.71 | 0.50 | 0.03 | 98.95 | 47.05 | 39.14 | 13.80 |
| Lava_102 | 48.43 | 1.71 | 5.19 | - | 8.2 | 0.20 | 12.78 | 21.66 | 0.52 | - | 98.68 | 47.25 | 38.79 | 13.96 |
| Lava_103 | 48.65 | 1.68 | 5.11 | 0.01 | 8.25 | 0.19 | 12.91 | 21.53 | 0.52 | 0.02 | 98.88 | 46.87 | 39.11 | 14.02 |
| Lava_104 | 48.66 | 1.66 | 5.26 | 0.01 | 8.33 | 0.21 | 13.03 | 21.44 | 0.56 | 0.00 | 99.17 | 46.54 | 39.35 | 14.11 |
| Lava_105 | 48.7 | 1.60 | 5.37 | - | 8.29 | 0.18 | 12.87 | 21.41 | 0.58 | - | 99.01 | 46.76 | 39.11 | 14.13 |
| Lava_106 | 48.89 | 1.56 | 5.12 | - | 8.19 | 0.22 | 13.07 | 21.38 | 0.60 | 0.00 | 99.03 | 46.52 | 39.57 | 13.91 |
| Lava_107 | 48.95 | 1.54 | 5.11 | 0.02 | 8.26 | 0.19 | 12.93 | 21.63 | 0.57 | - | 99.20 | 46.95 | 39.05 | 14.00 |
| Lava_108 | 48.73 | 1.59 | 5.39 | - | 8.43 | 0.21 | 12.9 | 21.11 | 0.63 | - | 99.00 | 46.25 | 39.33 | 14.42 |
| Lava_109 | 48.85 | 1.65 | 5.42 | - | 8.32 | 0.23 | 12.9 | 21.16 | 0.66 | 0.00 | 99.18 | 46.40 | 39.36 | 14.24 |
| Lava_110 | 49 | 1.53 | 4.99 | 0.01 | 8.08 | 0.22 | 13.22 | 21.53 | 0.57 | 0.01 | 99.16 | 46.57 | 39.79 | 13.64 |
| Lava_111 | 48.53 | 1.71 | 5.29 | 0.02 | 8.21 | 0.20 | 12.92 | 21.69 | 0.55 | - | 99.11 | 47.08 | 39.02 | 13.91 |
| Lava_112 | 49.54 | 1.47 | 4.42 | 0.02 | 7.94 | 0.22 | 13.36 | 21.73 | 0.53 | 0.00 | 99.23 | 46.71 | 39.96 | 13.32 |
| Lava_113 | 49.37 | 1.58 | 4.67 | - | 7.83 | 0.16 | 13.35 | 21.66 | 0.50 | 0.01 | 99.13 | 46.73 | 40.08 | 13.19 |
| Lava_114 | 50.07 | 1.28 | 3.91 | 0.02 | 7.6 | 0.19 | 13.96 | 21.86 | 0.45 | - | 99.33 | 46.30 | 41.14 | 12.56 |
| Lava_115 | 49.06 | 1.52 | 4.72 | 0.01 | 7.9 | 0.21 | 13.21 | 21.82 | 0.49 | 0.01 | 98.95 | 47.06 | 39.64 | 13.30 |
| Lava_116 | 50.35 | 1.30 | 3.58 | 0.01 | 7.82 | 0.22 | 13.88 | 21.48 | 0.48 | 0.01 | 99.13 | 45.80 | 41.18 | 13.02 |
| Lava_117 | 50.23 | 1.33 | 3.89 | 0.02 | 7.79 | 0.19 | 13.68 | 21.64 | 0.51 | - | 99.28 | 46.28 | 40.71 | 13.00 |
| Lava_118 | 49.23 | 1.47 | 4.77 | 0.02 | 8.07 | 0.20 | 13.28 | 21.57 | 0.52 | - | 99.13 | 46.54 | 39.87 | 13.59 |
| Lava_119 | 49.37 | 1.35 | 4.6 | 0.02 | 7.74 | 0.19 | 13.58 | 21.75 | 0.47 | - | 99.06 | 46.59 | 40.47 | 12.94 |
| Lava_120 | 49.7 | 1.43 | 4.27 | 0.01 | 8.39 | 0.19 | 13.56 | 21.49 | 0.53 | - | 99.57 | 45.81 | 40.22 | 13.96 |
| Lava_121 | 50.3 | 1.07 | 3.62 | 0.04 | 6.9 | 0.16 | 14.31 | 21.79 | 0.29 | 0.01 | 98.48 | 46.28 | 42.29 | 11.44 |
| Lava_122 | 50.44 | 1.06 | 3.57 | - | 6.97 | 0.16 | 14.39 | 21.91 | 0.32 | 0.02 | 98.84 | 46.25 | 42.27 | 11.48 |
| Lava_123 | 49.94 | 1.12 | 3.89 | 0.01 | 6.96 | 0.18 | 14.18 | 21.95 | 0.31 | 0.02 | 98.57 | 46.59 | 41.88 | 11.53 |
| Lava_124 | 48.61 | 1.43 | 5.28 | - | 7.42 | 0.16 | 13.48 | 22.08 | 0.36 | - | 98.82 | 47.35 | 40.23 | 12.42 |
| Lava_125 | 47.86 | 1.69 | 5.92 | - | 7.7 | 0.19 | 13.07 | 21.96 | 0.38 | - | 98.76 | 47.58 | 39.40 | 13.02 |
| Lava_126 | 48.05 | 1.87 | 6.17 | - | 7.87 | 0.17 | 12.83 | 21.95 | 0.44 | 0.00 | 99.35 | 47.78 | 38.85 | 13.37 |
| Lava_127 | 48.72 | 1.59 | 5.34 | - | 7.5 | 0.13 | 13.34 | 22 | 0.43 | 0.01 | 99.06 | 47.40 | 39.99 | 12.61 |
| Lava_128 | 48.31 | 1.89 | 5.14 | - | 8.67 | 0.21 | 12.68 | 21.36 | 0.54 | 0.01 | 98.82 | 46.67 | 38.55 | 14.79 |
| Lava_129 | 48.74 | 1.84 | 4.93 | 0.01 | 8.46 | 0.20 | 12.75 | 21.6 | 0.53 | 0.00 | 99.07 | 47.01 | 38.61 | 14.37 |
| Lava_130 | 48.22 | 1.87 | 5.25 | 0.01 | 8.52 | 0.21 | 12.77 | 21.62 | 0.47 | - | 98.93 | 46.96 | 38.59 | 14.45 |
| Lava_131 | 48.08 | 1.77 | 5.67 | 0.01 | 8.05 | 0.18 | 12.84 | 21.59 | 0.46 | 0.00 | 98.66 | 47.20 | 39.06 | 13.74 |
| Lava_132 | 47.99 | 1.81 | 5.71 | - | 8.2 | 0.19 | 12.84 | 21.79 | 0.52 | 0.01 | 99.06 | 47.31 | 38.79 | 13.90 |
| Lava_133 | 47.74 | 1.87 | 5.85 | 0.02 | 8.11 | 0.17 | 12.86 | 21.87 | 0.46 | 0.02 | 98.97 | 47.45 | 38.82 | 13.73 |
| Lava_134 | 47.7 | 1.90 | 6.05 | 0.00 | 8.1 | 0.18 | 12.75 | 21.81 | 0.48 | 0.00 | 98.98 | 47.54 | 38.67 | 13.78 |
| Lava_135 | 48.03 | 2.07 | 5.63 | - | 8.64 | 0.19 | 12.54 | 21.47 | 0.48 | 0.01 | 99.07 | 47.02 | 38.21 | 14.77 |
| Lava_136 | 46.9 | 2.50 | 6.57 | 0.01 | 9.02 | 0.20 | 12.09 | 21.34 | 0.49 | 0.01 | 99.12 | 47.21 | 37.21 | 15.58 |
| Lava_137 | 50.19 | 1.09 | 3.64 | 0.01 | 6.82 | 0.16 | 14.31 | 21.95 | 0.31 | - | 98.49 | 46.52 | 42.20 | 11.28 |
| Lava_138 | 50.4 | 1.05 | 3.5 | 0.01 | 6.84 | 0.16 | 14.43 | 22.05 | 0.35 | - | 98.78 | 46.45 | 42.30 | 11.25 |
| Lava_139 | 50.06 | 1.07 | 3.66 | - | 6.87 | 0.17 | 14.28 | 21.89 | 0.34 | 0.00 | 98.34 | 46.45 | 42.17 | 11.38 |
| Lava_140 | 50.3 | 1.08 | 3.5 | - | 7.06 | 0.17 | 14.35 | 21.75 | 0.30 | 0.00 | 98.51 | 46.05 | 42.28 | 11.67 |
| Lava_141 | 50.24 | 1.09 | 3.57 | 0.00 | 7.05 | 0.17 | 14.35 | 21.92 | 0.32 | - | 98.71 | 46.26 | 42.13 | 11.61 |
| Lava_142 | 50.56 | 1.04 | 3.58 | - | 7.1 | 0.20 | 14.34 | 21.63 | 0.31 | - | 98.75 | 45.90 | 42.34 | 11.76 |
| Lava_143 | 50.4 | 1.10 | 3.72 | - | 7 | 0.18 | 14.22 | 21.92 | 0.34 | - | 98.89 | 46.47 | 41.95 | 11.58 |
| Lava_144 | 50.2 | 1.08 | 3.7 | 0.01 | 7 | 0.20 | 14.29 | 21.74 | 0.32 | 0.02 | 98.56 | 46.17 | 42.23 | 11.60 |
| Lava_145 | 50.29 | 1.10 | 3.69 | - | 6.93 | 0.16 | 14.33 | 21.79 | 0.35 | 0.02 | 98.65 | 46.23 | 42.30 | 11.48 |
| Lava_146 | 50.12 | 1.14 | 3.88 | - | 6.92 | 0.18 | 14.32 | 21.93 | 0.36 | 0.00 | 98.85 | 46.41 | 42.16 | 11.43 |
| Lava_147 | 49.38 | 1.26 | 4.48 | - | 7.11 | 0.15 | 13.68 | 21.99 | 0.38 | 0.00 | 98.43 | 47.22 | 40.87 | 11.92 |
| Lava_148 | 50.76 | 1.20 | 3.2 | - | 7.76 | 0.23 | 14.03 | 21.17 | 0.45 | - | 98.81 | 45.29 | 41.76 | 12.96 |
| Lava_149 | 50.55 | 1.20 | 3.32 | 0.01 | 8.08 | 0.23 | 13.69 | 21.14 | 0.45 | - | 98.68 | 45.47 | 40.97 | 13.56 |
| Lava_150 | 50.66 | 1.16 | 3.1 | - | 8.03 | 0.25 | 13.85 | 21.27 | 0.44 | 0.01 | 98.77 | 45.44 | 41.17 | 13.39 |
| Lava_151 | 50.59 | 1.26 | 3.38 | 0.01 | 7.97 | 0.19 | 13.55 | 21.28 | 0.53 | 0.02 | 98.78 | 45.91 | 40.67 | 13.42 |
| Lava_152 | 49.87 | 1.44 | 4.05 | - | 8.17 | 0.19 | 13.39 | 21.33 | 0.56 | 0.00 | 99.00 | 46.03 | 40.21 | 13.76 |
| Lava_153 | 48.71 | 1.59 | 4.71 | 0.03 | 8.3 | 0.21 | 12.84 | 21.36 | 0.52 | - | 98.28 | 46.74 | 39.09 | 14.17 |
| Lava_154 | 49.29 | 1.52 | 4.32 | - | 8.26 | 0.23 | 13.03 | 21.4 | 0.52 | - | 98.57 | 46.54 | 39.43 | 14.02 |
| Lava_155 | 50.16 | 1.32 | 3.32 | - | 7.93 | 0.25 | 13.68 | 21.28 | 0.44 | 0.02 | 98.40 | 45.76 | 40.93 | 13.31 |
| Lava_156 | 50.1 | 1.31 | 3.5 | - | 8.07 | 0.20 | 13.61 | 21.44 | 0.49 | 0.01 | 98.72 | 45.93 | 40.57 | 13.50 |
| Lava_157 | 49.79 | 1.29 | 3.9 | - | 7.74 | 0.21 | 13.6 | 21.54 | 0.50 | 0.02 | 98.59 | 46.32 | 40.69 | 12.99 |
| Lava_158 | 50.08 | 1.28 | 3.66 | 0.02 | 7.71 | 0.23 | 13.93 | 21.5 | 0.42 | - | 98.83 | 45.84 | 41.33 | 12.83 |
| Lava_159 | 50.17 | 1.23 | 3.55 | 0.01 | 7.66 | 0.20 | 13.9 | 21.57 | 0.39 | 0.01 | 98.69 | 46.00 | 41.25 | 12.75 |
| Lava_160 | 50.27 | 1.22 | 3.6 | 0.02 | 7.5 | 0.22 | 13.98 | 21.75 | 0.37 | 0.01 | 98.94 | 46.22 | 41.34 | 12.44 |
| Lava_161 | 49.01 | 1.55 | 4.57 | 0.03 | 7.97 | 0.17 | 13.41 | 21.4 | 0.40 | - | 98.52 | 46.24 | 40.32 | 13.44 |
| Lava_162 | 49.53 | 1.45 | 3.99 | - | 8.2 | 0.21 | 13.67 | 21.35 | 0.42 | - | 98.82 | 45.65 | 40.67 | 13.68 |
| Lava_163 | 50.41 | 1.22 | 3.66 | - | 7.39 | 0.19 | 14.05 | 21.57 | 0.39 | 0.00 | 98.88 | 46.00 | 41.69 | 12.30 |
| Lava_164 | 50.26 | 1.20 | 3.68 | - | 7.45 | 0.18 | 14.06 | 21.65 | 0.37 | 0.00 | 98.85 | 46.04 | 41.60 | 12.37 |
| Lava_165 | 50.16 | 1.20 | 3.86 | - | 7.31 | 0.19 | 13.79 | 21.74 | 0.43 | 0.02 | 98.71 | 46.62 | 41.15 | 12.24 |
| Lava_166 | 50.1 | 1.24 | 3.64 | 0.01 | 7.56 | 0.23 | 13.92 | 21.57 | 0.39 | - | 98.65 | 46.05 | 41.35 | 12.60 |
| Lava_167 | 50.11 | 1.42 | 3.69 | - | 8.32 | 0.24 | 13.6 | 20.91 | 0.47 | 0.02 | 98.78 | 45.14 | 40.85 | 14.02 |
| Lava_168 | 50.15 | 1.27 | 3.59 | 0.01 | 7.99 | 0.22 | 13.71 | 21.26 | 0.43 | 0.01 | 98.63 | 45.65 | 40.96 | 13.39 |
| Lava_169 | 49.66 | 1.34 | 4.14 | - | 7.82 | 0.18 | 12.91 | 21.86 | 0.49 | - | 98.41 | 47.60 | 39.11 | 13.29 |
| Lava_170 | 48.41 | 1.72 | 1.91 | 0.01 | 8.1 | 0.19 | 12.83 | 21.83 | 0.53 | - | 98.55 | 47.45 | 38.80 | 13.74 |
| Lava_171 | 48.67 | 1.43 | 4.67 | 0.01 | 7.8 | 0.17 | 13.35 | 22.19 | 0.45 | - | 98.75 | 47.36 | 39.65 | 12.99 |
| Lava_172 | 48.98 | 1.44 | 4.2 | - | 7.54 | 0.21 | 13.64 | 22.26 | 0.48 | - | 98.83 | 47.24 | 40.27 | 12.49 |
| Lava_173 | 50.12 | 1.19 | 3.6 | - | 7.29 | 0.17 | 14 | 22.07 | 0.39 | - | 98.84 | 46.72 | 41.24 | 12.05 |

| Analysis | SiO_2_ | TiO_2_ | Al_2_O3 | Cr_2_O_3_ | FeO | MnO | MgO | CaO | Na2O | NiO | Total | Wo | En | Fs |
| --- | --- | --- | --- | --- | --- | --- | --- | --- | --- | --- | --- | --- | --- | --- |
| Strombolian _1 | 50.91 | 1.31 | 3.57 | 0.02 | 8.01 | 0.20 | 14.05 | 22.95 | 0.53 | 0.00 | 101.54 | 47.08 | 40.10 | 12.82 |
| Strombolian _2 | 50.39 | 1.12 | 4.14 | 0.02 | 7.27 | 0.16 | 14.46 | 23.22 | 0.32 | 0.02 | 101.12 | 47.37 | 41.05 | 11.58 |
| Strombolian _3 | 51.35 | 1.23 | 3.33 | 0.00 | 8.51 | 0.25 | 13.89 | 22.61 | 0.53 | 0.00 | 101.69 | 46.54 | 39.78 | 13.67 |
| Strombolian _4 | 51.06 | 1.26 | 3.34 | 0.02 | 8.08 | 0.22 | 14.1 | 22.8 | 0.47 | 0.00 | 101.35 | 46.79 | 40.26 | 12.94 |
| Strombolian _5 | 51.11 | 1.20 | 3.17 | 0.01 | 8.44 | 0.24 | 14.09 | 22.78 | 0.47 | 0.00 | 101.52 | 46.52 | 40.03 | 13.45 |
| Strombolian _6 | 49.1 | 1.63 | 4.94 | 0.01 | 8.66 | 0.21 | 13.33 | 22.6 | 0.52 | 0.00 | 101.02 | 47.17 | 38.72 | 14.11 |
| Strombolian _7 | 47.99 | 2.13 | 5.8 | 0.00 | 9.2 | 0.20 | 12.6 | 22.68 | 0.56 | 0.00 | 101.17 | 47.86 | 36.99 | 15.15 |
| Strombolian _8 | 47.62 | 1.98 | 6.61 | 0.01 | 8.53 | 0.16 | 13.05 | 22.98 | 0.43 | 0.01 | 101.38 | 48.08 | 37.99 | 13.93 |
| Strombolian _9 | 48.08 | 1.95 | 5.42 | 0.01 | 9 | 0.20 | 13.16 | 22.55 | 0.56 | 0.00 | 100.93 | 47.09 | 38.24 | 14.67 |
| Strombolian _10 | 48.24 | 1.76 | 4.5 | 0.00 | 8.8 | 0.22 | 13.26 | 22.72 | 0.51 | 0.00 | 100.00 | 47.30 | 38.41 | 14.30 |
| Strombolian _11 | 46.7 | 2.21 | 6.47 | 0.01 | 8.96 | 0.20 | 12.77 | 22.82 | 0.49 | 0.01 | 100.65 | 47.96 | 37.34 | 14.70 |
| Strombolian _12 | 46.97 | 2.10 | 5.8 | 0.00 | 9.04 | 0.17 | 12.69 | 22.62 | 0.50 | 0.00 | 99.90 | 47.79 | 37.30 | 14.91 |
| Strombolian _13 | 46.89 | 2.25 | 5.88 | 0.00 | 9.29 | 0.20 | 12.74 | 22.62 | 0.52 | 0.01 | 100.40 | 47.52 | 37.24 | 15.23 |
| Strombolian _14 | 47.18 | 1.89 | 6.39 | 0.00 | 8.25 | 0.16 | 13.19 | 23.12 | 0.39 | 0.01 | 100.58 | 48.26 | 38.30 | 13.44 |
| Strombolian _15 | 49.65 | 1.23 | 3.72 | 0.01 | 7.72 | 0.19 | 14.63 | 22.79 | 0.38 | 0.02 | 100.34 | 46.35 | 41.40 | 12.25 |
| Strombolian _16 | 49.28 | 1.26 | 4.22 | 0.00 | 7.63 | 0.17 | 14.31 | 22.79 | 0.43 | 0.00 | 100.09 | 46.84 | 40.92 | 12.24 |
| Strombolian _17 | 48.34 | 1.68 | 5.83 | 0.00 | 8.18 | 0.14 | 13.11 | 23.17 | 0.44 | 0.00 | 100.90 | 48.48 | 38.16 | 13.36 |
| Strombolian _18 | 48.08 | 1.81 | 5.77 | 0.00 | 8.71 | 0.19 | 13.17 | 22.71 | 0.52 | 0.00 | 100.96 | 47.48 | 38.31 | 14.21 |
| Strombolian _19 | 50.08 | 1.30 | 4.41 | 0.00 | 7.78 | 0.18 | 14.11 | 23 | 0.44 | 0.03 | 101.33 | 47.22 | 40.31 | 12.47 |
| Strombolian _20 | 47.92 | 2.04 | 5.82 | 0.02 | 8.72 | 0.17 | 12.9 | 22.79 | 0.48 | 0.00 | 100.86 | 47.93 | 37.75 | 14.32 |
| Strombolian_21 | 48.86 | 1.67 | 4.22 | 0.00 | 8.63 | 0.24 | 12.06 | 21.82 | 0.68 | 0.00 | 98.18 | 46.82 | 40.59 | 12.59 |
| Strombolian_22 | 48.48 | 1.63 | 4.91 | 0.00 | 8.15 | 0.17 | 12.32 | 22.31 | 0.52 | 0.02 | 98.46 | 48.70 | 37.42 | 13.89 |
| Strombolian_23 | 49.38 | 1.45 | 4.27 | 0.00 | 8.03 | 0.24 | 12.62 | 22.13 | 0.52 | 0.01 | 98.65 | 48.15 | 38.21 | 13.64 |

| Analysis | SiO_2_ | TiO2 | Al_2_O_3_ | Cr_2_O_3_ | FeO | MnO | MgO | CaO | Na2O | NiO | Total | Wo | En | Fs |
| --- | --- | --- | --- | --- | --- | --- | --- | --- | --- | --- | --- | --- | --- | --- |
| Tephra_1 | 46.64 | 1.63 | 6.67 | 0.10 | 7.74 | 0.13 | 13.18 | 23.58 | 0.33 | 0.00 | 100.00 | 49.17 | 38.24 | 12.60 |
| Tephra_2 | 47.43 | 1.76 | 5.38 | 0.00 | 8.69 | 0.19 | 13.26 | 22.51 | 0.48 | 0.03 | 99.74 | 47.15 | 38.64 | 14.21 |
| Tephra_3 | 45.96 | 2.01 | 7.44 | 0.23 | 7.87 | 0.11 | 12.97 | 23.37 | 0.32 | 0.01 | 100.29 | 49.14 | 37.95 | 12.92 |
| Tephra_4 | 46.89 | 1.75 | 6.69 | 0.22 | 7.56 | 0.12 | 13.34 | 23.77 | 0.31 | 0.04 | 100.70 | 49.28 | 38.48 | 12.23 |
| Tephra_5 | 49.54 | 1.14 | 3.98 | 0.16 | 6.92 | 0.13 | 15.05 | 23.05 | 0.26 | 0.00 | 100.23 | 46.67 | 42.40 | 10.94 |
| Tephra_6 | 49.46 | 1.36 | 3.95 | 0.00 | 7.96 | 0.25 | 14.43 | 22.54 | 0.58 | 0.00 | 100.53 | 46.16 | 41.12 | 12.72 |
| Tephra_7 | 49.94 | 1.24 | 3.53 | 0.01 | 8.19 | 0.20 | 14.47 | 22.48 | 0.48 | 0.00 | 100.55 | 45.87 | 41.08 | 13.04 |
| Tephra_8 | 49.17 | 1.23 | 3.57 | 0.00 | 8.43 | 0.20 | 14.17 | 22.48 | 0.47 | 0.00 | 99.72 | 46.09 | 40.42 | 13.49 |
| Tephra_9 | 48.99 | 1.37 | 3.7 | 0.00 | 8.54 | 0.24 | 14.05 | 22.17 | 0.46 | 0.02 | 99.54 | 45.82 | 40.40 | 13.78 |
| Tephra_10 | 47.16 | 1.95 | 5.28 | 0.01 | 8.94 | 0.18 | 13.13 | 22.44 | 0.51 | 0.00 | 99.60 | 47.06 | 38.31 | 14.63 |
| Tephra_11 | 48.92 | 1.43 | 4.05 | 0.00 | 8.31 | 0.22 | 13.93 | 22.74 | 0.53 | 0.04 | 100.158 | 46.78 | 39.87 | 13.34 |
| Tephra_12 | 49.02 | 1.49 | 4.47 | 0.02 | 8.08 | 0.19 | 12.76 | 22.31 | 0.51 | 0.00 | 98.38 | 48.11 | 38.29 | 13.60 |
| Tephra_13 | 48.06 | 1.64 | 5.04 | 0.00 | 8.42 | 0.22 | 12.33 | 22.08 | 0.57 | 0.00 | 98.73 | 48.20 | 37.45 | 14.35 |
| Tephra_14 | 49.12 | 1.45 | 4.2 | 0.01 | 8.03 | 0.22 | 12.90 | 22.31 | 0.49 | 0.00 | 98.75 | 47.95 | 38.58 | 13.47 |
| Tephra_15 | 49.21 | 1.48 | 3.9 | 0.00 | 8.25 | 0.21 | 12.88 | 22.25 | 0.56 | 0.00 | 98.61 | 47.74 | 38.45 | 13.82 |
| Tephra_16 | 48.46 | 1.50 | 4.56 | 0.00 | 8.02 | 0.19 | 12.93 | 22.46 | 0.47 | 0.00 | 98.33 | 48.08 | 38.52 | 13.40 |
| Tephra_17 | 48.15 | 1.59 | 4.77 | 0.00 | 8.16 | 0.20 | 12.72 | 22.17 | 0.55 | 0.00 | 98.42 | 47.95 | 38.28 | 13.77 |
| Tephra_18 | 50.45 | 1.16 | 3.5 | 0.01 | 7.72 | 0.22 | 13.96 | 22.41 | 0.46 | 0.00 | 99.89 | 46.82 | 40.59 | 12.59 |

Table S3. Clinopyroxene Elemental Data.

| Analysis | SiO2 | TiO2 | Al2O3 | FeO | MnO | MgO | CaO | Na2O | K2O | P2O5 | S ppm | Cl ppm | Total | Type | Host Min. | Kd | Host # |
| --- | --- | --- | --- | --- | --- | --- | --- | --- | --- | --- | --- | --- | --- | --- | --- | --- | --- |
| Tephra_47 | 48.69 | 1.78 | 16.82 | 7.70 | 0.18 | 2.62 | 6.25 | 5.26 | 3.46 |  | 164 | 1631 | 92.97 | inc | PLG | 0.14 | Tephra_157 |
| Tephra_48 | 51.35 | 0.98 | 17.51 | 9.80 |  | 2.72 | 5.39 | 4.80 | 4.52 | 1.33 | 442 | 4530 | 98.96 | inc | OLI | 0.27 | Tephra_44 |
| Tephra_49 | 49.82 | 1.92 | 16.99 | 10.11 |  | 3.52 | 7.84 | 3.72 | 2.96 | 0.88 | 483 | 1755 | 98.06 | inc | CPX | 0.20 | Tephra_1 |
| Tephra_50 | 49.16 | 1.44 | 19.25 | 7.02 | 0.18 | 2.62 | 8.99 | 5.21 | 2.73 |  | 79 | 1440 | 96.76 | inc | PLG | 0.17 | Tephra_148 |
| Tephra_51 | 50.11 | 2.04 | 15.47 | 9.75 | 0.23 | 3.38 | 7.10 | 4.34 | 3.15 |  | 151 | 1508 | 95.76 | inc | CPX | 0.22 | Tephra_12 |
| Tephra_52 | 49.88 | 1.98 | 15.60 | 9.74 | 0.20 | 3.32 | 7.05 | 4.64 | 3.22 |  | 168 | 1737 | 95.85 | inc | CPX | 0.23 | Tephra_13 |
| Tephra_53 | 49.84 | 2.00 | 15.59 | 9.76 | 0.22 | 3.29 | 7.35 | 4.85 | 3.15 |  | 206 | 1648 | 96.27 | inc | CPX | 0.21 | Tephra_14 |
| Tephra_54 | 49.65 | 1.93 | 16.30 | 10.18 | 0.23 | 3.02 | 6.71 | 5.12 | 3.45 |  | 0 | 1767 | 96.87 | inc | CPX | 0.19 | Tephra_15 |
| Tephra_55 | 49.51 | 2.01 | 15.88 | 9.53 | 0.21 | 3.27 | 7.26 | 4.92 | 3.18 |  | 203 | 1633 | 95.99 | inc | CPX | 0.21 | Tephra_16 |
| Tephra_56 | 49.72 | 2.11 | 15.87 | 9.70 | 0.23 | 3.30 | 7.24 | 4.96 | 3.17 |  | 256 | 1745 | 96.53 | inc | CPX | 0.22 | Tephra_17 |
| Tephra_57 | 49.65 | 2.00 | 15.95 | 9.98 | 0.22 | 3.07 | 7.09 | 5.16 | 3.34 |  | 235 | 2553 | 96.77 | inc | OLI | 0.25 | Tephra_19 |
| Tephra_58 | 49.92 | 1.82 | 16.34 | 10.05 | 0.24 | 2.52 | 7.96 | 4.84 | 3.12 |  | 331 | 1684 | 97.07 | inc | CPX | 0.14 | Tephra_21 |
| Tephra_59 | 49.08 | 1.92 | 16.61 | 9.66 | 0.23 | 3.12 | 7.32 | 4.90 | 3.46 |  | 386 | 1785 | 96.57 | inc | PLG | 0.10 | Tephra_165 |
| Tephra_60 | 49.02 | 1.95 | 16.41 | 9.75 | 0.22 | 3.11 | 7.34 | 4.85 | 3.24 |  | 369 | 1902 | 96.18 | inc | PLG | 0.22 | Tephra_166 |
| Tephra_61 | 49.96 | 1.86 | 16.13 | 9.33 | 0.21 | 2.82 | 6.25 | 4.23 | 3.96 | 1.22 | 256 | 1961 | 96.23 | inc | PLG | 0.10 | Tephra_131 |
| Tephra_62 | 49.78 | 1.87 | 16.18 | 9.82 | 0.21 | 3.11 | 6.74 | 4.35 | 3.43 | 1.06 | 162 | 1902 | 96.79 | inc | PLG | 0.07 | Tephra_132 |
| Tephra_63 | 50.64 | 1.66 | 17.19 | 7.34 | 0.17 | 2.45 | 6.16 | 5.51 | 3.83 | 1.12 | 278 | 1714 | 96.31 | inc | PLG | 0.07 | Tephra_142 |
| Tephra_64 | 48.97 | 1.88 | 15.67 | 9.97 | 0.22 | 3.31 | 7.75 | 4.77 | 3.02 | 1.13 | 242 | 1622 | 96.91 | inc | OLI | 0.30 | Tephra_23 |
| Tephra_65 | 49.44 | 1.83 | 15.79 | 9.94 | 0.18 | 3.28 | 7.82 | 3.89 | 3.02 | 0.99 | 331 | 1731 | 96.44 | inc | OLI | 0.30 | Tephra_24 |
| Tephra_66 | 50.06 | 1.86 | 15.78 | 9.65 | 0.20 | 2.85 | 6.40 | 5.43 | 3.60 | 1.05 | 198 | 1622 | 97.09 | inc | OLI | 0.27 | Tephra_25 |
| Tephra_67 | 49.88 | 1.92 | 15.75 | 9.70 | 0.19 | 2.91 | 6.54 | 5.46 | 3.62 | 1.08 | 65 | 1647 | 97.23 | inc | OLI | 0.27 | Tephra_26 |
| Tephra_68 | 49.51 | 1.31 | 15.86 | 10.44 | 0.26 | 3.21 | 7.27 | 5.35 | 2.69 | 0.80 | 678 | 2355 | 97.11 | inc | OLI | 0.28 | Tephra_27 |
| Tephra_69 | 48.98 | 1.98 | 16.57 | 9.41 | 0.23 | 3.35 | 7.96 | 4.94 | 3.02 | 0.97 | 414 | 1695 | 97.69 | emb |  |  |  |
| Tephra_70 | 47.57 | 1.85 | 16.66 | 9.78 | 0.20 | 3.17 | 8.01 | 5.17 | 2.98 | 0.90 | 602 | 1905 | 96.63 | emb |  |  |  |
| Tephra_71 | 47.35 | 1.77 | 16.40 | 9.69 | 0.17 | 3.28 | 8.03 | 5.14 | 2.88 | 0.82 | 536 | 1803 | 95.84 | emb |  |  |  |
| Tephra_72 | 47.21 | 1.86 | 16.40 | 9.84 | 0.23 | 3.13 | 8.32 | 5.05 | 2.89 | 0.89 | 642 | 1748 | 96.15 | emb |  |  |  |
| Tephra_73 | 48.34 | 1.81 | 16.49 | 9.52 | 0.20 | 3.43 | 8.01 | 4.63 | 2.83 |  | 740 | 1739 | 95.61 | emb |  |  |  |
| Tephra_74 | 48.63 | 1.86 | 15.97 | 9.54 | 0.22 | 3.48 | 8.01 | 4.92 | 2.88 |  | 573 | 1769 | 95.83 | emb |  |  |  |
| Tephra_75 | 48.85 | 1.80 | 16.60 | 9.96 | 0.19 | 3.29 | 8.11 | 4.75 | 2.99 |  | 628 | 1768 | 96.88 | emb |  |  |  |
| Analysis | SiO2 | TiO2 | Al2O3 | FeO | MnO | MgO | CaO | Na2O | K2O | P2O5 | S ppm | Cl ppm | Total | Type | Host Min. | Kd | Host # |
| Tephra_76 | 49.19 | 1.83 | 17.01 | 9.61 | 0.21 | 3.08 | 7.89 | 5.05 | 3.13 |  | 490 | 1771 | 97.30 | emb |  |  |  |
| Strombolian_17 | 48.74 | 2.60 | 16.28 | 11.03 |  | 3.49 | 8.14 | 3.91 | 3.25 | 1.02 | 617 | 1951 | 98.81 | emb |  |  |  |
| Strombolian_18 | 49.57 | 2.15 | 16.58 | 10.38 |  | 3.30 | 7.27 | 3.92 | 3.54 | 1.17 | 139 | 1702 | 98.08 | emb |  |  |  |
| Strombolian_19 | 48.89 | 2.10 | 16.79 | 10.45 |  | 3.38 | 7.40 | 3.74 | 3.31 | 1.09 | 92 | 1285 | 97.30 | inc | CPX | 0.20 | Strombolian_17 |
| Strombolian_20 | 50.90 | 1.85 | 17.48 | 8.28 |  | 3.21 | 7.63 | 3.50 | 3.47 | 1.18 | 107 | 1405 | 97.67 | inc | CPX | 0.26 | Strombolian_20 |
| Strombolian_21 | 45.55 | 1.41 | 18.69 | 9.67 |  | 3.95 | 10.99 | 4.15 | 2.23 | 0.83 | 1331 | 2059 | 98.01 | inc | OLI | 0.23 | Strombolian_33 |
| Strombolian_22 | 48.35 | 1.83 | 16.75 | 9.72 | 0.24 | 3.00 | 6.38 | 6.01 | 4.40 |  | 290 | 1816 | 96.94 | inc |  |  |  |
| Strombolian_23 | 48.88 | 1.93 | 16.55 | 9.72 | 0.25 | 3.12 | 7.08 | 5.47 | 3.94 |  | 109 | 1868 | 97.15 | inc |  |  |  |
| Strombolian_24 | 49.21 | 1.96 | 16.08 | 9.43 | 0.25 | 3.05 | 6.93 | 5.05 | 4.03 |  | 230 | 1811 | 96.23 | inc |  |  |  |
| Strombolian_25 | 49.68 | 1.88 | 16.69 | 8.94 | 0.21 | 2.95 | 7.13 | 5.44 | 4.00 |  | 160 | 1738 | 97.13 | inc |  |  |  |
| Strombolian_26 | 49.28 | 1.95 | 15.82 | 9.75 | 0.22 | 3.14 | 6.76 | 5.45 | 3.99 |  | 212 | 2056 | 96.62 | inc |  |  |  |
| Strombolian_27 | 47.01 | 1.95 | 15.37 | 11.33 | 0.30 | 4.10 | 8.16 | 5.22 | 3.31 |  | 800 | 2042 | 97.15 | inc | PLG | 0.03 | Strombolian_91 |
| Strombolian_28 | 49.36 | 2.00 | 15.91 | 9.64 | 0.21 | 3.12 | 7.16 | 5.30 | 3.67 |  | 112 | 2080 | 96.60 | emb |  |  |  |
| Strombolian_29 | 51.34 | 1.68 | 16.43 | 8.19 | 0.29 | 2.69 | 6.77 | 5.55 | 3.71 |  | 222 | 1845 | 96.89 | inc | CPX | 0.24 | Strombolian_21 |
| Strombolian_30 | 50.18 | 1.86 | 16.63 | 9.09 | 0.20 | 2.82 | 6.72 | 5.11 | 3.72 |  | 94 | 1871 | 96.54 | inc | CPX | 0.21 | Strombolian_22 |
| Strombolian_31 | 51.18 | 2.23 | 16.81 | 8.20 | 0.20 | 2.42 | 6.85 | 5.49 | 3.59 |  | 253 | 1700 | 97.21 | inc | CPX | 0.19 | Strombolian_23 |
| Strombolian_32 | 50.23 | 2.00 | 17.47 | 8.38 | 0.20 | 2.13 | 6.57 | 5.38 | 4.83 | 1.05 | 240 | 2481 | 98.55 | inc | OLI | 0.21 | Strombolian_24 |
| Strombolian_33 | 50.75 | 1.95 | 17.81 | 8.43 | 0.16 | 2.46 | 6.01 | 5.40 | 4.73 | 1.14 | 178 | 2332 | 99.11 | inc | OLI | 0.24 | Strombolian_25 |
| Lava_8 | 50.91 | 1.74 | 17.90 | 9.71 | 0.23 | 2.26 | 5.44 | 6.08 | 4.18 |  | 341 | 2246 | 98.76 | inc | CPX | 0.14 | Lava_169 |
| Lava_9 | 50.57 | 1.64 | 17.63 | 9.43 | 0.24 | 2.31 | 5.61 | 5.94 | 4.09 |  | 278 | 2095 | 97.74 | inc | CPX | 0.15 | Lava_170 |
| Lava_10 | 54.20 | 1.87 | 17.71 | 6.16 | 0.20 | 2.32 | 5.23 | 5.82 | 4.20 |  | 1298 | 2943 | 98.33 | inc | CPX | 0.21 | Lava_171 |
| Lava_11 | 54.73 | 1.87 | 17.32 | 5.68 | 0.18 | 2.20 | 4.96 | 5.78 | 4.49 |  | 1215 | 3283 | 97.84 | inc | CPX | 0.23 | Lava_172 |
| Lava_12 | 53.52 | 2.16 | 17.48 | 6.62 | 0.22 | 1.92 | 4.71 | 5.83 | 4.78 |  | 1439 | 3715 | 97.97 | inc | CPX | 0.15 | Lava_173 |
| Lava_13 | 50.92 | 1.87 | 17.88 | 9.59 | 0.21 | 2.09 | 5.09 | 6.47 | 4.29 |  | 209 | 2383 | 98.70 | emb |  |  |  |
| Xenolith_10 | 50.49 | 1.82 | 16.57 | 9.48 | 0.22 | 2.56 | 7.35 | 5.21 | 3.85 |  | 205 | 2181 | 97.82 | inc | OLI | 0.23 | Xenolith_14 |
| Xenolith_11 | 49.83 | 1.85 | 16.94 | 8.83 | 0.21 | 2.36 | 7.08 | 5.83 | 4.02 |  | 270 | 2292 | 97.25 | inc | OLI | 0.20 | Xenolith_11 |
| Xenolith_12 | 50.15 | 1.74 | 16.94 | 9.18 | 0.21 | 2.19 | 7.67 | 5.56 | 3.67 |  | 232 | 2085 | 97.58 | inc | OLI | 0.20 | Xenolith_12 |

Table S4. Melt Inclusions and Glass Elemental Data. Inclusion data is only considered when the Fe-Mg exchange coefficient (Kd) is within 0.27 ± 0.03.
